# Modeling Myeloma Dissemination *In Vitro* with hMSC-interacting Subpopulations of INA-6 Cells and Their Aggregation/Detachment Dynamics

**DOI:** 10.1158/2767-9764.CRC-23-0411

**Published:** 2024-04-29

**Authors:** Martin Kuric, Susanne Beck, Doris Schneider, Wyonna Rindt, Marietheres Evers, Jutta Meißner-Weigl, Sabine Zeck, Melanie Krug, Marietta Herrmann, Tanja Nicole Hartmann, Ellen Leich, Maximilian Rudert, Denitsa Docheva, Anja Seckinger, Dirk Hose, Franziska Jundt, Regina Ebert

**Affiliations:** 1 Department of Musculoskeletal Tissue Regeneration, University of Würzburg, Würzburg, Germany.; 2 University Hospital Heidelberg, Institute of Pathology, Heidelberg, Germany.; 3 Department of Internal Medicine II, University Hospital Würzburg, Würzburg, Germany.; 4 University of Würzburg, Institute of Pathology, Comprehensive Cancer Center Mainfranken, Würzburg, Germany.; 5 University Hospital Würzburg, IZKF Research Group Tissue Regeneration in Musculoskeletal Diseases, Würzburg, Germany.; 6 Department of Internal Medicine I, Faculty of Medicine and Medical Center, University of Freiburg, Freiburg, Germany.; 7 Orthopedic Department, Clinic König-Ludwig-Haus, University of Würzburg, Würzburg, Germany.; 8 Department of Hematology and Immunology, Vrije Universiteit Brussel, Jette, Belgium.

## Abstract

Multiple myeloma involves early dissemination of malignant plasma cells across the bone marrow; however, the initial steps of dissemination remain unclear. Human bone marrow–derived mesenchymal stromal cells (hMSC) stimulate myeloma cell expansion (e.g., IL6) and simultaneously retain myeloma cells via chemokines (e.g., CXCL12) and adhesion factors. Hence, we hypothesized that the imbalance between cell division and retention drives dissemination. We present an *in vitro* model using primary hMSCs cocultured with INA-6 myeloma cells. Time-lapse microscopy revealed proliferation and attachment/detachment dynamics. Separation techniques (V-well adhesion assay and well plate sandwich centrifugation) were established to isolate MSC-interacting myeloma subpopulations that were characterized by RNA sequencing, cell viability, and apoptosis. Results were correlated with gene expression data (*n* = 837) and survival of patients with myeloma (*n* = 536). On dispersed hMSCs, INA-6 saturate hMSC surface before proliferating into large homotypic aggregates, from which single cells detached completely. On confluent hMSCs, aggregates were replaced by strong heterotypic hMSC–INA-6 interactions, which modulated apoptosis time dependently. Only INA-6 daughter cells (nMA-INA6) detached from hMSCs by cell division but sustained adherence to hMSC-adhering mother cells (MA-INA6). Isolated nMA-INA6 indicated hMSC autonomy through superior viability after IL6 withdrawal and upregulation of proliferation-related genes. MA-INA6 upregulated adhesion and retention factors (*CXCL12*), that, intriguingly, were highly expressed in myeloma samples from patients with longer overall and progression-free survival, but their expression decreased in relapsed myeloma samples. Altogether, *in vitro* dissemination of INA-6 is driven by detaching daughter cells after a cycle of hMSC-(re)attachment and proliferation, involving adhesion factors that represent a bone marrow–retentive phenotype with potential clinical relevance.

**Significance:**

Novel methods describe *in vitro* dissemination of myeloma cells as detachment of daughter cells after cell division. Myeloma adhesion genes were identified that counteract *in vitro* detachment with potential clinical relevance.

## Introduction

Multiple myeloma arises from clonal expansion of malignant plasma cells in the bone marrow (BM). At diagnosis, myeloma cells have disseminated to multiple sites in the skeleton and, in some cases, to “virtually any tissue” (1, 2). However, the mechanism through which myeloma cells initially disseminate remains unclear.

Dissemination is a multistep process involving invasion, intravasation, intravascular arrest, extravasation, and colonization (3). To initiate dissemination, myeloma cells overcome adhesion, retention, and dependency on the BM microenvironment, which could involve the loss of adhesion factors such as CD138 (4, 5). BM retention is mediated by multiple factors: First, chemokines (CXCL12 and CXCL8) produced by mesenchymal stromal cells (MSC), which attract plasma cells and prime their cytoskeleton and integrins for adhesion (6, 7). Second, myeloma cells must overcome the anchorage and physical boundaries of the extracellular matrix (ECM), consisting of, for example, fibronectin, collagens, and proteoglycans such as decorin (8–11). Simultaneously, ECM provides signals inducing myeloma cell cycle arrest or progression the cell cycle (8, 10). ECM is also prone to degradation, which is common in several osteotropic cancers, and is the cause of osteolytic bone disease. This is driven by a “vicious cycle” that maximizes bone destruction by extracting growth factors (EGF and TGF-β) that are stored in calcified tissues (12). Third, direct contact with MSCs physically anchors myeloma cells to the BM (3, 13). Fourth, to disseminate to distant sites, myeloma cells require, at least partially, independence from essential growth and survival signals provided by MSCs in the form of soluble factors or cell adhesion signaling (5, 14, 15). For example, the VLA4 (Myeloma)–VCAM1 (MSC)-interface activates NFκB in both myeloma and MSCs, inducing IL6 expression in MSCs. The independence from MSCs is then acquired through autocrine survival signaling (16, 17). In short, anchorage of myeloma cells to MSCs or ECM is a “double-edged sword”: adhesion counteracts dissemination, but also presents signaling cues for growth, survival, and drug resistance (18).

To address this ambiguity, we developed an *in vitro* coculture system modeling diverse adhesion modalities to study dissemination, growth, and survival of myeloma cells and human MSCs (hMSC). Cocultures of hMSCs and the myeloma cell line INA-6 replicated tight interactions and aggregate growth, akin to “microtumors” in Ghobrial metastasis concept (19). We characterized the growth conformations of hMSCs and INA-6 as homotypic aggregation versus heterotypic hMSC adherence and their effects on myeloma cell survival. We tracked INA-6 detachments from aggregates and hMSCs, thereby identifying a potential “disseminated” subpopulation lacking strong adhesion. We developed innovative techniques [V-well adhesion assay and well plate sandwich centrifugation (WPSC)] to separate weakly and strongly adherent subpopulations for the subsequent analysis of differential gene expression and cell survival. Notably, our strategy resolves the differences in gene expression and growth behavior between cells of one cell population in “direct” contact with MSCs. In contrast, previous methods differentiated between “direct” and “indirect” cell-cell contact using transwell inserts (20). To evaluate whether genes mediating adhesion and growth characteristics of INA-6 were associated with patient survival, we analyzed publicly available datasets (21, 22).

## Materials and Methods

See Supplementary Data for a complete method list and description.

### Ethics Statement

Primary hMSCs were collected with the written informed consent of all patients. The procedure was conducted in accordance with recognized ethical guidelines (Helsinki Declaration) and approved by the local Ethics Committee of the University of Würzburg (Würzburg, Germany; 186/18).

### Cultivation and Coculturing of Primary hMSCs and INA-6

Primary hMSCs were obtained from the femoral head of 34 patients with non-myeloma (Supplementary Table S1: 21 male and 13 female, mean age 68.9 ± 10.6) undergoing elective hip arthroplasty. The INA-6 cell line (DSMZ, catalog no. ACC-862, RRID:CVCL_5209, https://www.cellosaurus.org/CVCL_5209) was initially isolated from a pleural effusion sample obtained from an 80-year-old male with multiple myeloma (23, 24). hMSCs were not tested for *Mycoplasma*, whereas stocks of INA-6 were tested in this study (Supplementary Table S1) using the Venor GeM OneStep kit (Minerva Biolabs).

For each coculture, hMSCs were seeded 24 hours before INA-6 addition to generate the MSC-conditioned medium (CM). INA-6 cells were washed with PBS, resuspended in MSC medium, and added to hMSCs so that the coculture comprised 33% (v/v) of CM gathered directly from the respective hMSC donor. The cocultures were not substituted for IL6 (14).

### Cell Viability and Apoptosis Assay

Cell viability and apoptosis rates were measured using CellTiter-Glo Luminescent Cell Viability Assay and Caspase-Glo 3/7 Assay, respectively (Promega GmbH).

### Automated Fluorescence Microscopy

Microscopic images were acquired using an Axio Observer 7 (Zeiss) with a COLIBRI LED light source and motorized stage top using 5x and 10x magnification. The tiled images had an automatic 8%–10% overlap and were not stitched.

### Live Cell Imaging

hMSCs (stained with PKH26) were placed into an ibidi Stage Top Incubation System and equilibrated to 80% humidity and 5% CO_2_. INA-6 (2 × 10^3^ cells/cm^2^) were added directly before the start of acquisition. Brightfield and fluorescence images of up to 13 mm^2^ of the coculture area were acquired every 15 minutes for 63 hours. Each event of interest was manually analyzed and categorized into defined event parameters.

### V-well Adhesion Assay

INA-6 cells were arrested during mitosis by two treatments with thymidine, followed by nocodazole. Arrested INA-6 were released and added to 96 V-well plates (10^4^ cells/cm^2^) on top of confluent hMSCs and adhered for 1–3 hours. The coculture was stained with Calcein AM (Thermo Fisher Scientific) before nonadherent INA-6 were pelleted into the tip of the V-well (555 × *g*, 5–10 minutes). MSC-adhering INA-6 cells were manually detached by rapid pipetting. The pellet brightness was measured microscopically and the pellet was isolated by pipetting.

### Cell Cycle Profiling by Image Cytometry

Isolated INA-6 cells were fixed in 70% ice-cold ethanol, washed, resuspended in PBS, distributed in 96-well plates, and stained with Hoechst 33342. The plates were scanned at 5x magnification. A pre-trained convolutional neural network (Intellesis, Zeiss) was fine-tuned to segment the scans into single nuclei and exclude fragmented nuclei. Nuclei were filtered to exclude extremes of size roundness. The G_0_/G_1_ frequency was determined by Gaussian curve fitting.

### Well Plate Sandwich Centrifugation (WPSC)

hMSCs were grown to confluence in 96-well plates coated with collagen I (rat tail; Corning). INA-6 were added and the cells were allowed to adhere for 24 hours. A second plate (“catching plate”) was attached upside down to the top of the coculture plate. That “well plate sandwich” was turned around and the content of the coculture plate was centrifuged into the catching plate three times (40 seconds at 110 × *g*) while gently adding 30 µL of medium in between centrifugation steps. Non–MSC-adhering INA-6 cells were collected from the catching plate, whereas MSC-adhering INA-6 cells were isolated by digesting the coculture with accutase. For RNA sequencing (RNA-seq), all samples were purified using anti-CD45 magnetic-assisted cell sorting (Miltenyi Biotec B.V. & Co. KG).

### RNA Isolation

RNA was isolated using the NucleoSpin RNA II Purification Kit (Macherey-Nagel) according to the manufacturer's instructions. RNA was isolated from INA-6 cells cocultured with a unique hMSC donor (*n* = 5 for RNA sequencing, *n* = 11 for qPCR).

### RNA-seq, Differential Expression, and Functional Enrichment Analysis

RNA-seq was performed at the Core Unit Systems Medicine, University of Würzburg (Würzburg, Germany). mRNA was enriched with polyA beads. Fastq files were aligned to the GRCh38 reference genome using STAR (RRID:SCR_004463, https://scicrunch.org/resolver/RRID:SCR_004463) and raw read counts were generated using HTseq (RRID:SCR_005514, https://scicrunch.org/resolver/SCR_005514; refs. 25–27). Differential gene expression was analyzed using edgeR in R (version 3.6.3; RRID:SCR_012802, https://scicrunch.org/resolver/SCR_012802). Functional enrichment analysis was performed using Metascape (RRID:SCR_016620, https://scicrunch.org/resolver/SCR_016620; ref. 28).

### qRT-PCR

RNA (1 µg) was reverse transcribed using SuperScript IV reverse transcriptase (Thermo Fisher Scientific). qPCR was performed using 10 µL GoTaq qPCR Master Mix (Promega), 1:10 diluted cDNA, and 5 pmol of primers obtained from Biomers.net or Qiagen (Supplementary Table S3).

### Statistical Analysis

Inferential statistics were performed using Python (IPython, RRID:SCR_001658, https://scicrunch.org/resolver/SCR_001658; 3.10) packages pingouin (0.5.1) and statsmodels (0.14.0; refs. 29, 30). The figures were plotted using plotastic (0.0.1; ref. 31). Normality (for *n* ≥ 4) and sphericity were ensured using Mauchly's and Shapiro–Wilk tests, respectively. Datapoints were log_10_ transformed to convert the scale from multiplicative to additive or to fulfill sphericity requirements. *P*-value = 0.05 > * > 0.01 > ** > 10^−3^ > *** 10^−4^ > ****. *P* values were either adjusted (*P*-adj) or not adjusted (*P*-unc) for familywise error rate. Power calculations were not performed to determine the sample size.

### Patient Cohort, Analysis of Survival, and Expression

Survival and gene expression data were obtained as described previously (21, 22) and are available at the European Nucleotide Archive under accession numbers PRJEB36223 and PRJEB37100. The expression level was categorized into “high” and “low” using maxstat (maximally selected rank statistics) thresholds (32).

### Data Availability Statement

A detailed description of the methods is provided in the Supplementary Materials and Methods section. Raw tabular data and examples of analyses and videos are available in the github repository, https://github.com/markur4/Supplemental-INA-6-Subpopulations-and-Aggregation-Detachment-Dynamics. Raw RNA-seq data are available from the NCBI Gene Expression Omnibus (RRID:SCR_005012, https://www.ncbi.nlm.nih.gov/geo/query/acc.cgi?acc=GSE261423; GSE261423). Microscopy data are available at BioStudies (EMBL-EBI; RRID:SCR_004727, https://www.ebi.ac.uk/biostudies/bioimages/studies/S-BIAD1092?key=69bafe9c-74ff-492b-9e68-bd42655c4d1b; S-BIAD1092).

## Results

### INA-6 Cells Saturate hMSC interaction to Proliferate into Aggregates

**FIGURE 1:**
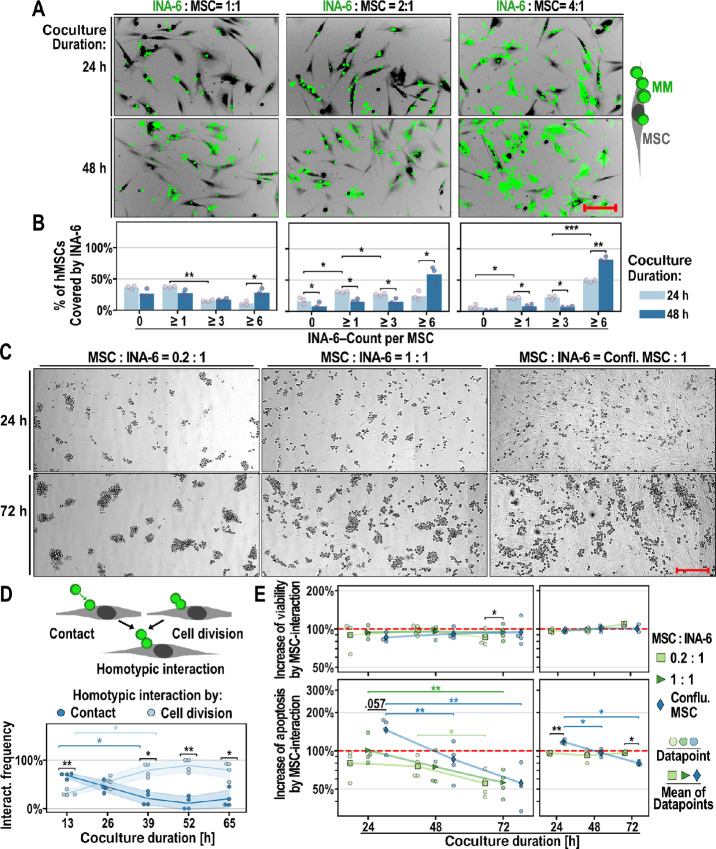
INA-6 growth conformations and survival on hMSCs. **A,** Interaction of INA-6 (green) with hMSCs (black, negative staining) at different INA-6 densities (constant hMSC densities). Scale bar = 200 µm. **B,** Frequency of single hMSCs (same as B) that are covered by INA-6 of varying group sizes. Technical replicates = three per datapoint; 100 single hMSCs were evaluated per technical replicate. **C,** Interaction of INA-6 with hMSCs at different hMSC densities (constant INA-6 densities). Scale bar = 300 µm. **D,** Two types of homotypic interaction: Attachment after cell contact and sustained attachment of daughter cells after cell division. Datapoints represent one of four independent time-lapse recordings, each evaluating 116 interaction events. **E,** Effects of hMSC-density on the viability (ATP, top) and apoptosis (Caspase3/7 activity, bottom). INA-6:MSC ratio = 4:1; Technical replicates = four per datapoint; **E, Left:** Signals were measured in INA-6 washed off from hMSCs and normalized by INA-6 cultured in MSC-CM (= red line; *n* = 4). **E, Right:** Signals were measured in cocultures and normalized by the sum of the signals measured in hMSC and INA-6 cultured separately ( = red line; *n* = 3). **Statistics:** Paired *t* test, two-factor RM-ANOVA. Datapoints represent independent cocultures with hMSCs from 3 (A, B, D, E right), 4 (E left) unique donors. Confl. = Confluent.

hMSCs are isolated as a heterogeneous cell population. To analyze whether INA-6 cells could adhere to every hMSC, we saturated hMSCs with INA-6. A seeding ratio of 1:4 (hMSC:INA-6) resulted in the occupation of 93% ± 6% of single hMSCs by one or more INA-6 cells within 24 hours after INA-6 addition, escalating to 98% after 48 hours (Fig. 1A and B). Therefore, most hMSCs provide an interaction surface for INA-6 cells.

INA-6 exhibits homotypic aggregation when cultured alone, a phenomenon observed in some freshly isolated myeloma samples (up to 100 cells after 6 hours; refs. 33, 34). Adding hMSCs at a 1:1 ratio led to smaller aggregates after 24 hours (size 1–5 cells), all of which were distributed over 52% ± 2% of all hMSCs (Fig. 1A and B). Intriguingly, INA-6 aggregation was notably absent when grown on confluent hMSCs, and occurred only when heterotypic interactions were limited to 0.2 hMSCs per INA-6 cell (Fig. 1C). We concluded that INA-6 cells prioritize heterotypic over homotypic interactions.

To monitor the formation of such aggregates, we conducted live cell imaging of hMSC/INA-6 cocultures for 63 hours. We observed that INA-6 cells adhered long after cytokinesis, constituting 55% ± 12% of all homotypic interactions between 13 and 26 hours, increasing to >75% for the remainder of the coculture (Fig. 1D). Therefore, homotypic INA-6 aggregates were mostly formed by cell division.

### Apoptosis of INA-6 Depends on Ratio Between Heterotypic and Homotypic Interaction

Although direct interaction with hMSCs has been shown to enhance myeloma cell survival through NFκB signaling (15), the impact of aggregation on myeloma cell viability during hMSC interaction remains unclear. To address this, we measured the cell viability (ATP) and apoptosis rates of INA-6 cells growing as homotypic aggregates compared with those in heterotypic interactions with hMSCs by modulating hMSC density (Fig. 1E). To equalize the background signaling caused by soluble MSC-derived factors, all cultures were incubated in hMSC-conditioned medium and the results were normalized to INA-6 cells cultured without direct hMSC contact (Fig. 1E, left).

INA-6 viability (ATP) was not affected by the direct adhesion of hMSCs at any density. However, apoptosis rates decreased over time [F(2, 6) = 23.29, *P*-unc = 1.49e-03, two-factor Repeated Measures (RM) ANOVA], interacting significantly with MSC density [F(4, 12) = 6.98, *P*-unc = 3.83e-3]. For example, 24 hours of adhesion to confluent MSCs increased apoptosis rates by 1.46 ± 0.37 fold, while culturing INA-6 cells on dispersed hMSCs (ratio 1:1) did not change the apoptosis rate (1.01 ± 0.26).

We presumed that sensitive apoptotic cells might have been lost when harvesting INA-6 cells from hMSCs. Hence, we measured survival parameters in the coculture and in hMSC and INA-6 cells cultured separately (Fig. 1E, right). We defined MSC interaction effects when the survival measured in the coculture differed from the sum of the signals measured from INA-6 and hMSCs alone. RM-ANOVA confirmed that adherence to confluent MSCs increased apoptosis rates of INA-6 cells 24 hours after adhesion and decreased after 72 hours [interaction between MSC density and time: F(2, 4) = 26.86, *P*-unc = 4.80e-03, two-factor RM-ANOVA], whereas INA-6 cells were unaffected when grown on dispersed hMSCs.

In summary, the growth conformation of INA-6 cells, measured as the ratio between homotypic aggregation and heterotypic MSC interactions, affected apoptosis rates of INA-6 cells.

### Single INA-6 Cells Detach Spontaneously from Aggregates of Critical Size

**FIGURE 2:**
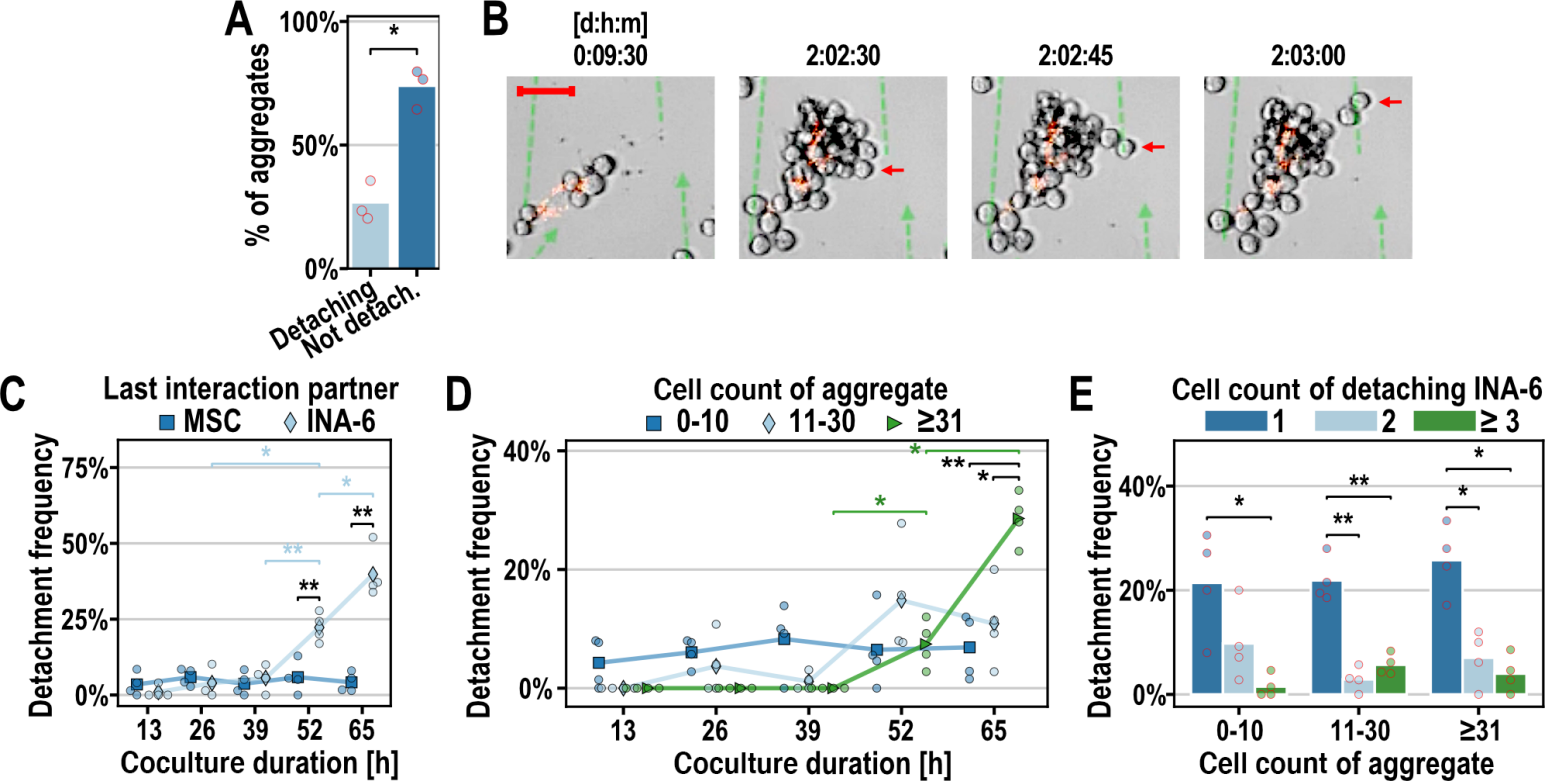
Time-lapse analysis of INA-6 detachment from INA-6 aggregates and hMSCs. **A,** Frequency of observed INA-6 aggregates that did or did not lose INA-6 cell(s). A total of 87 aggregates were evaluated per datapoint. **B,** Example of a “disseminating” INA-6 aggregate growing on fluorescently (PKH26) stained hMSC (from A–D). Dashed green lines are trajectories of detached INA-6 cells. Scale bar = 50 µm. **C**–**E**, Quantitative assessment of INA-6 detachments. A total of 45 detachment events were evaluated per datapoint. Seeding ratio INA-6:MSC = 4:1. **C,** Most INA-6 cells dissociated from another INA-6 cell and not from an hMSC [F(1, 3) = 298, *P*-unc = 4.2e-4]. **D,** Detachment frequency of aggregate size categories. **E,** Detachment frequency of INA-6 cells detaching as single, pairs or more than three cells. **Statistics:** (A): Paired *t* test; (C--E): Paired *t* test, two-factor RM-ANOVA; Datapoints represent three (A) or four (C–E) independent time-lapse recordings of cocultures with hMSCs from 2 (A) or 3 (C–E) unique donors.

Using time-lapse microscopy, we observed that 26% ± 8% of INA-6 aggregates growing on single hMSCs spontaneously shed INA-6 cells (Fig. 2A and B; Supplementary Video S1). Notably, all detached cells exhibited similar directional movements, suggesting entrainment in convective streams generated by temperature gradients within the incubation chamber. INA-6 predominantly detached from other INA-6 cells or aggregates (Fig. 2C), indicating weaker adhesive forces in homotypic interactions than in heterotypic interactions. The detachment frequency increased after 52 hours, when most aggregates that shed INA-6 cells were categorized as large (>30 cells; Fig. 2D). Because approximately 10–20 INA-6 cells already fully covered a single hMSC, we suggest that myeloma cell detachment depended not only on hMSC saturation, but also required a minimum aggregate size. Interestingly, INA-6 detached mostly as single cells, independent of aggregate size categories [F(2, 6) = 4.68, *P*‑unc = 0.059, two-factor RM-ANOVA] (Fig. 2E), showing that aggregates remained mostly stable despite losing cells.

### Cell Division Generates a Daughter Cell Detached from hMSC

**FIGURE 3:**
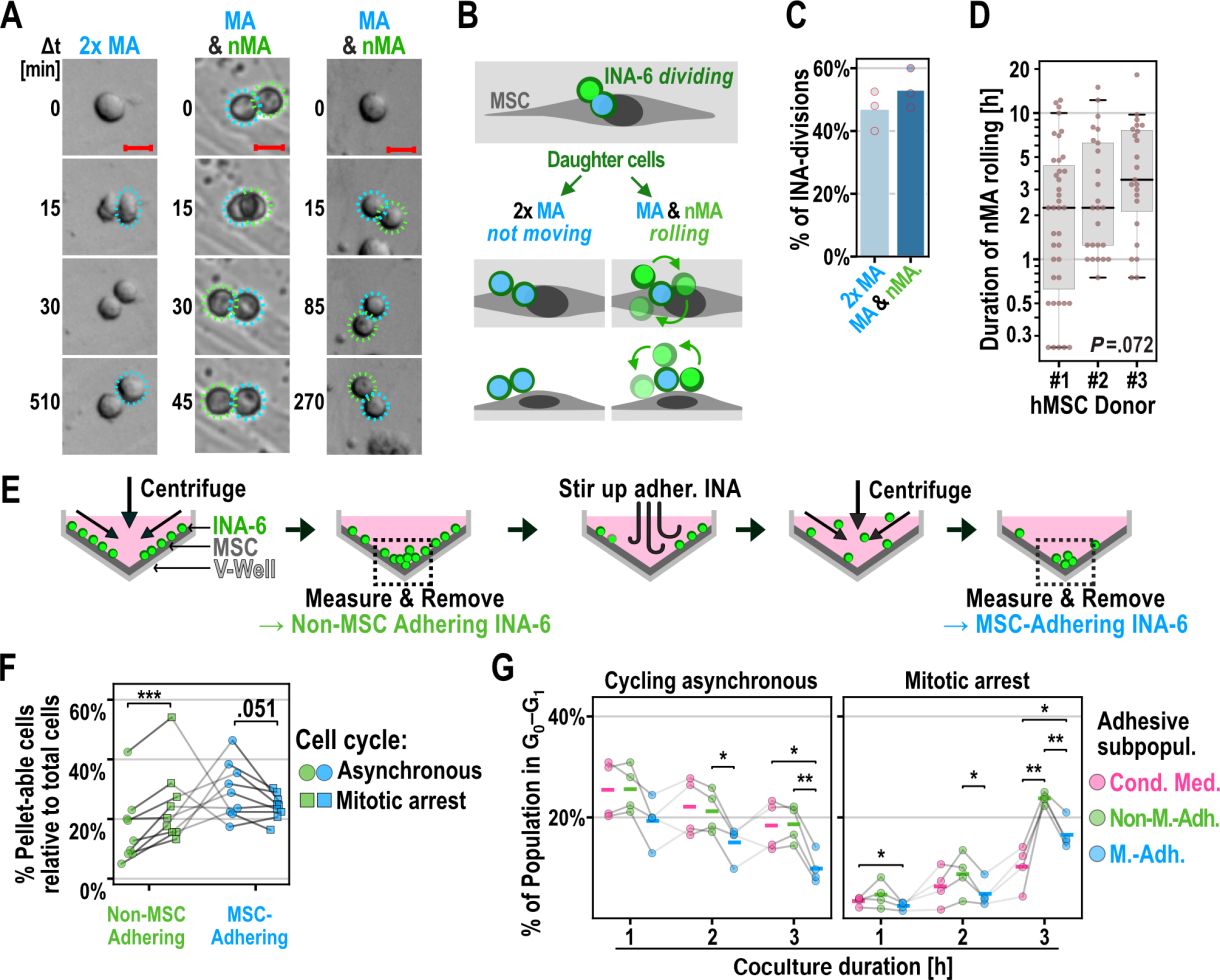
Detachment of INA-6 daughter cells after cell division. **A–D,** INA-6 divisions in interaction with confluent hMSCs. Seeding ratio INA-6:MSC = 4:20. **A,** Three examples of dividing INA-6 cells generating either two MA, or one MA and one nMA daughter cells as described in G. Dashed circles mark mother cells (white), MA cell (blue), and first position of nMA cell (green). Scale bar: 20 µm. **B,** Cell division of MSC-adhering (MA) mother cell can yield one mobile non–MSC-adhering (nMA) daughter cell. **C,** Frequencies of INA-6 pairs defined in A and B per observed cell division. A total of 65 divisions were evaluated for each of three independent time-lapse recordings. **D,** Rolling duration of nMA cells after division did not depend on hMSC donor [H(2) = 5.250, *P*-unc = 0y.072]. Datapoints represent single nMA cells after division. **E**–**G**, Adhesive and cell cycle assessment of MSC-interacting INA-6 subpopulations using the V-Well assay. **E,** Schematic of V-Well Assay (see Supplementary Fig. S1 for detailed analysis). MSC-interacting subpopulations were separated by subsequent centrifugation and removal of the pellet. The pellet size was quantified by its total fluorescence brightness. Adhering subpopulations were resuspended by rough pipetting. **F,** Relative cell pellet sizes of adhesive INA-6 subpopulations that cycle either asynchronously or were synchronized at mitosis. Gray lines in-between points connect dependent measurements of cocultures (*n* = 9) that shared the same hMSC-donor and INA-6 culture. Cocultures were incubated for three different durations (1, 2, and 3 hours after INA-6 addition). Timepoints were pooled, since time did not show an effect on cell adhesion [F(2,4) = 1.414, *P*-unc = 0.343]. Factorial RM-ANOVA shows an interaction between cell cycle and the kind of adhesive subpopulation [F(1, 8) = 42.67, *P*-unc = 1.82e-04]. Technical replicates = 4 per datapoint. **G,** Cell cycles were profiled in cells gathered from the pellets of four independent cocultures (*n* = 4) and the frequency of G_0_–G_1_ cells are displayed depending on coculture duration (see Supplementary Fig. S3 for cell cycle profiles). Four technical replicates were pooled after pelleting. **Statistics:** D: Kruskal–Wallis H-test. F: Paired *t* test. G: Paired *t* test, two-factor RM-ANOVA. Datapoints represent INA-6 from independent cocultures with hMSCs from 3 unique donors.

We suspected that cell division drives detachment because we observed that MSC-adhering INA-6 cells could generate daughter cells that “roll over” the mother cell (Fig. 3A; Supplementary Video S2). We recorded and categorized the movement of INA-6 daughter cells in confluent hMSCs after cell division. Half of all INA-6 divisions yielded two daughter cells that remained stationary, indicating hMSC adherence (Fig. 3B and C; Supplementary Video S3). The other half of division events generated one hMSC-adhering (MA) cell and one non–hMSC-adhering (nMA) cell, which rolled around the MA cell for a median time of 2.5 hours post division (Q_1_ = 1.00 hours, Q_3_ = 6.25 hours) until it stopped and readhered to the hMSC monolayer (Fig. 3D; Supplementary Video S2; Supplementary Video S4). Thus, cell division establishes a time window in which one daughter cell can detach.

To validate that cell division reduced adhesion, we measured both the size and cell cycle profile of the nMA and MA populations using an enhanced V-well assay (method described in Fig. 3E; Supplementary Fig. S1 and S2). For comparison, we fully synchronized and arrested INA-6 cells at mitosis and released their cell cycle immediately before addition to the hMSC monolayer, rendering them more likely to divide while adhering. Mitotic arrest significantly increased the number of nMA cells and decreased the number of MA cells (Fig. 3F). Furthermore, the nMA population contained significantly more cells cycling in the G_0_/G_1_-phase than the MA population, both in synchronously and asynchronously cycling INA-6 (Fig. 3G; Supplementary Fig. S3 and S4). The number of nMA INA-6 cells increased because of a higher cell division frequency. Taken together, we showed that INA-6 detach from aggregates by generating one temporarily detached daughter cell after cell division, a process that potentially contributes to the initiation of dissemination.

### WPSC Separates hMSC-interacting INA-6 Subpopulations

**FIGURE 4:**
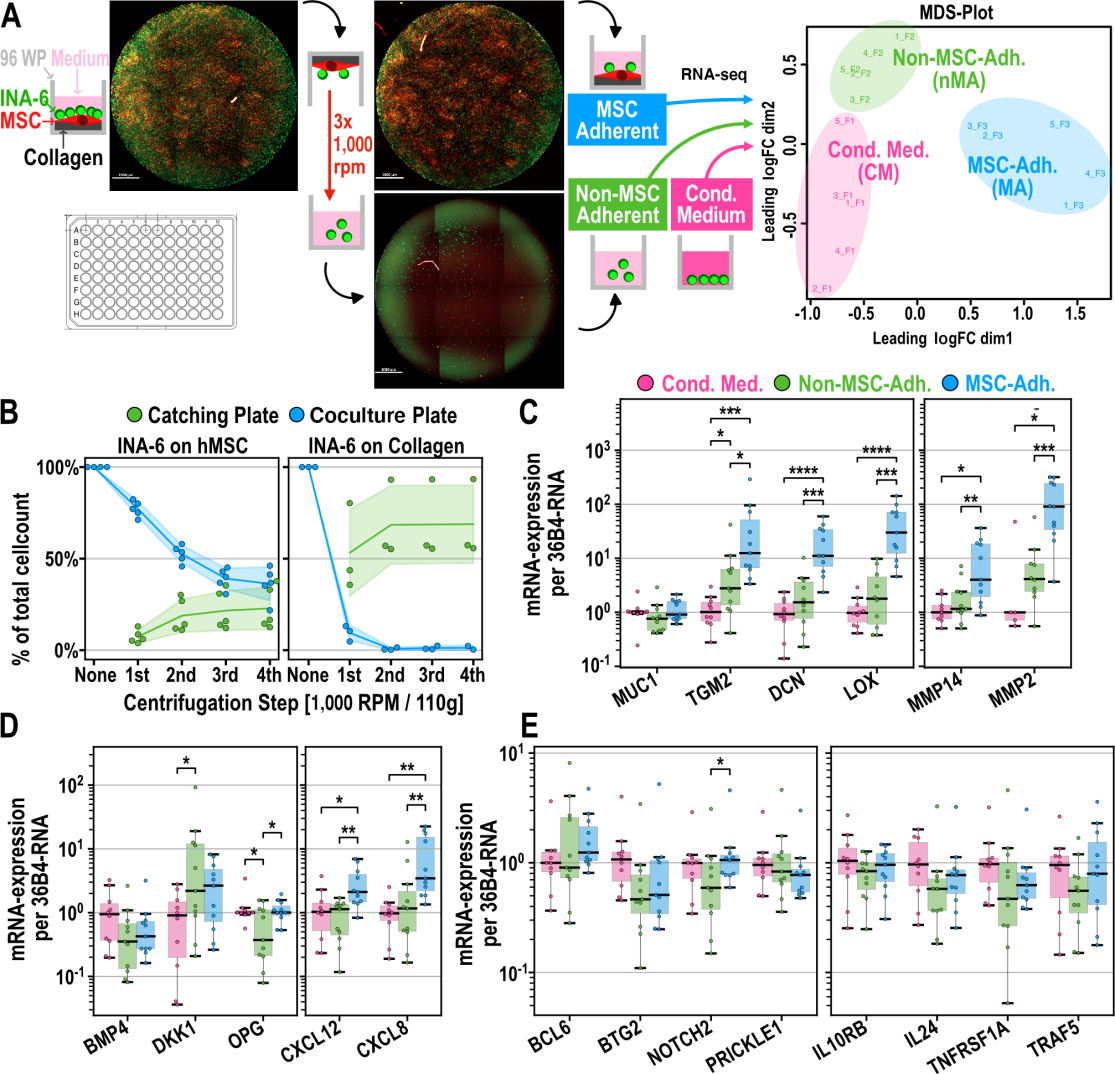
Separation and gene expression of INA-6 subpopulations. **A,** Schematic of WPSC separating nMA-INA6 from MA-INA6. A coculture 96-well plate is turned upside down and attached on top of a “catching plate,” forming a “well-plate sandwich.” nMA-INA6 cells are collected in the catching plate by subsequent rounds of centrifugation and gentle washing. MA-INA6 are enzymatically dissociated from hMSCs or by rough pipetting. Subsequent RNA-seq of MSC-interacting subpopulations reveals distinct expression clusters [right, multidimensional scaling plot (*n* = 5)]. **B,** Separation was microscopically tracked after each centrifugation step. **C–E,** qRT-PCR of genes derived from RNA-seq results. Expression was normalized to the median of CM-INA6. Samples include those used for RNA-seq and six further cocultures (*n* = 11; non-detects were discarded). **C,** Adhesion factors, ECM proteins and matrix metalloproteinases. **D,** Factors involved in bone remodeling and bone homing chemokines. **E,** Factors involved in (immune) signaling. **Statistics**: (C–E): Paired *t*-test. Datapoints represent the mean of three (B–E) technical replicates. INA-6 were isolated from independent cocultures with hMSCs from 5 (A, B), 9 (C--E) unique donors.

To separate nMA and MA cells for further analysis, we developed the method WPSC, outlined in Fig. 4A. To equalize the background signaling caused by MSC-derived factors and to focus on differences within directly MSC-interacting INA-6 subpopulations, all cultures were incubated in hMSC-CM from the respective donors and compared with INA-6 incubation in CM without hMSCs.

Microscopic tracking of nMA and MA INA-6 cell numbers during each WPSC separation step revealed successful separation after the third centrifugation step, whereas CM-treated INA-6 cells required only one centrifugation step (Fig. 4B). Thus, WPSC generated cell numbers that were suitable for subsequent analyses.

### RNA-seq of Non–MSC-adhering and MSC-adhering Subpopulations

To characterize the subpopulations separated by WPSC, we conducted RNA-seq, revealing 1,291 differentially expressed genes between nMA *vs* CM, 484 between MA *vs* CM, and 195 between MA *vs* nMA. We validated RNA-seq and found that the differential expression of 18 genes correlated with those measured with qPCR for each pairwise comparison (Fig. 4C–E; Supplementary Fig. S5): nMA *vs* CM [ρ (16) = 0.803, *P* = 6.09e-5], MA *vs* CM [ρ (16) = 0.827, *P* = 2.30e-5], and MA *vs* nMA cells [ρ (16) = 0.746, *P* = 3.74e-4] (Spearman rank correlation). One of the 18 genes (*MUC1*) measured by qPCR showed a mean expression opposite to that obtained by RNA-seq (nMA *vs* CM), although the difference was insignificant (Fig. 4C). For nMA *vs* CM, the difference in expression measured by qPCR was significant for only two of the 11 genes (*DKK1* and *OPG*), whereas the other genes (*BCL6*, *BMP4*, *BTG2*, *IL10RB*, *IL24*, *NOTCH2*, *TNFRSF1A*, *TRAF5*) only confirmed the tendency measured by RNA-seq (Fig. 4C–E). For MA *vs* CM, qPCR validated the significant upregulation of seven genes (*TGM2*, *DCN*, *LOX*, *MMP14*, *MMP2*, *CXCL12*, *CXCL8*), whereas the downregulation of *BMP4* was insignificant.

### Non–MSC-adhering INA-6 and MSC-adhering INA-6 Have Distinct Expression Patterns of Proliferation or Adhesion, Respectively

**FIGURE 5:**
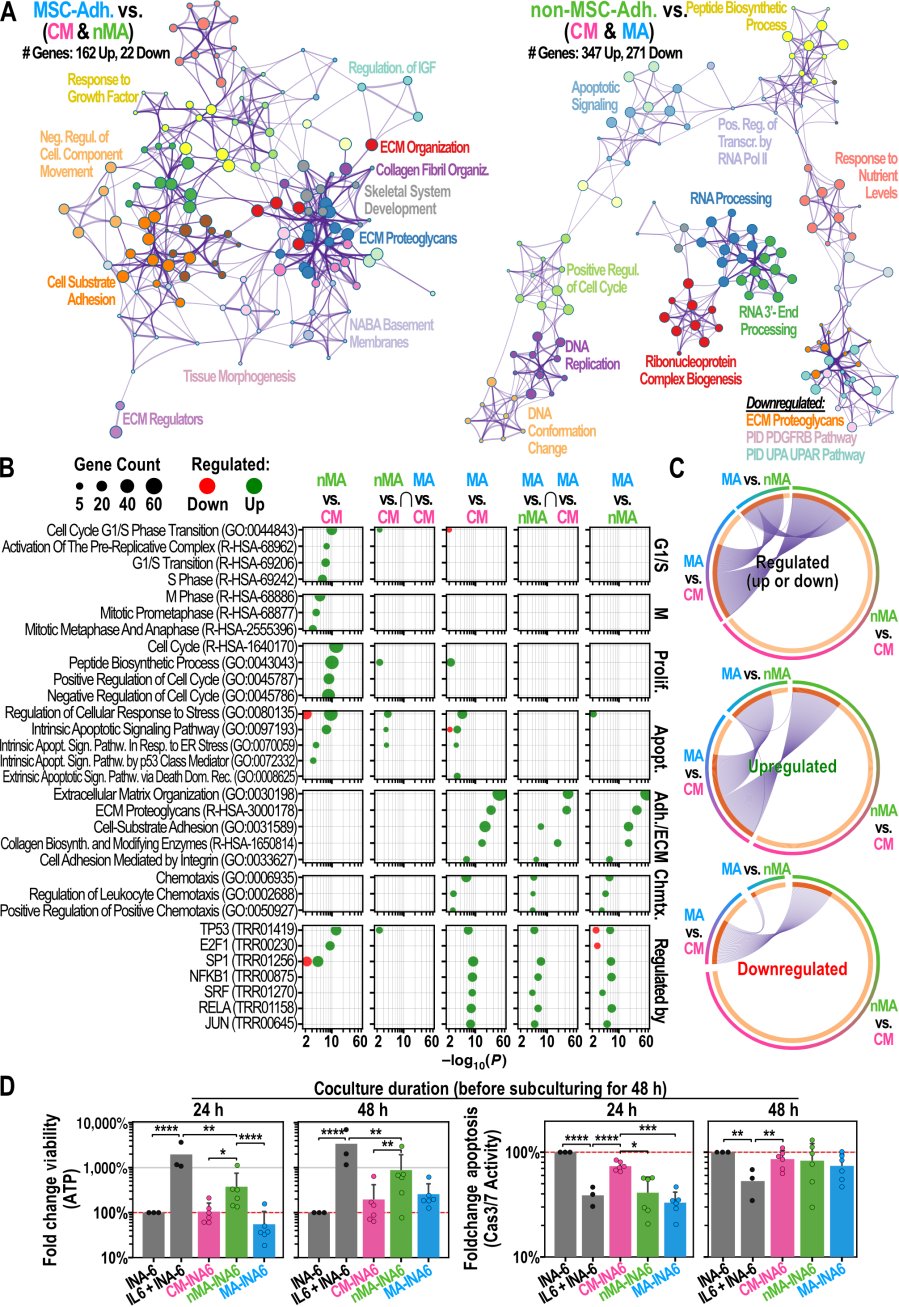
Functional analysis of MSC-interacting subpopulations (**A**–**C**): Functional enrichment analysis of differentially expressed genes (from RNA-seq) using Metascape. **A,** Gene ontology (GO) cluster analysis of gene lists that are unique for MA (left) or nMA (right) INA-6. Circle nodes represent subsets of input genes falling into similar GO term. Node size grows with the number of input genes. Node color defines a shared parent GO term. Two nodes with a similarity score > 0.3 are linked. **B,** Enrichment analysis of pairwise comparisons between MA subpopulations and their overlaps (arranged in columns). GO terms were manually picked and categorized (arranged in rows). Raw Metascape results are shown in Supplementary Fig. S6. For each GO term, the *P* values (*x* axis) and the counts of matching input genes (circle size) were plotted. The lowest row shows enrichment of gene lists from the TRRUST database. **C,** Circos plots by Metascape. Sections of a circle represent lists of differentially expressed genes. Purple lines connect same genes appearing in two gene lists. ∩: Overlapping groups, MA: MSC-adhering, nMA: non–MSC-adhering, CM: MSC-conditioned medium. **D,** INA-6 were cocultured on confluent hMSC for 24 or 48 hours, separated by WPSC and subcultured for 48 hours under IL6 withdrawal (*n* = 6), except the control (IL6 + INA-6; *n* = 3). Signals were normalized (red line) to INA-6 cells grown without hMSCs and IL6 (*n* = 3). Statistics (D): Paired *t* test, two-factor RM-ANOVA. Datapoints represent the mean of four technical replicates. INA-6 were isolated from independent cocultures with hMSCs from 6 unique donors.

To functionally characterize the unique transcriptional patterns in nMA-INA6 and MA-INA6, we generated lists of genes that were differentially expressed versus the other two subpopulations [termed nMA *vs* (MA & CM) and MA *vs* (nMA & CM)]. Functional enrichment analysis was performed, and the enriched terms were displayed as ontology clusters (Fig. 5A). nMA-INA6 upregulated genes enriched with loosely connected term clusters associated with proliferation (e.g., “positive regulation of cell cycle”). MA-INA6 upregulated genes enriched with tightly connected term clusters related to cell adhesion and the production of ECM factors (e.g., “cell-substrate adhesion”). Similar ontology terms were enriched in the gene lists obtained from pairwise comparisons (nMA vs. CM, MA vs. CM, and MA vs. nMA; Fig. 5B). In particular, nMA *vs* CM (but not MA vs. CM) upregulated genes that were enriched with “G_1_–S transition,” showing that WPSC isolated nMA daughter cells after cell division.

To check for similarities between lists of differentially expressed genes from hMSC-interacting subpopulations, we performed enrichment analysis on gene lists from the overlaps (“∩”) between all pairwise comparisons (Fig. 5B; Supplementary Fig. S6), and showed the extent of these overlaps in circos plots (Fig. 5C). The overlap between MA *vs* CM and nMA *vs* CM showed neither enrichment with proliferation- nor adhesion-related terms but with apoptosis-related terms. A direct comparison of MSC-interacting subpopulations (MA vs. nMA) showed a major overlap with MA vs. CM (Fig. 5C, middle). This overlap was enriched with terms related to adhesion but not proliferation. Hence, MA-INA6 and nMA-INA6 mostly differed in their expression of adhesion genes.

To assess whether nMA-INA6 and MA-INA6 were regulated by separate transcription factors, we examined the enrichment of curated regulatory networks from the Transcriptional Regulatory Relationships Unravelled by Sentence-based Text-mining (TRRUST) database (Fig. 5B, bottom). All the lists were enriched for p53 regulation. E2F1 regulation was observed only in genes upregulated in nMA *vs* CM and downregulated in MA *vs* nMA. Gene lists involving MA-INA6 were enriched in regulation by subunits of NFκB (NFKB1/p105 and RELA/p65) and factors of immediate early response (SRF, JUN). Correspondingly, NFκB and JUN are known to regulate the expression of adhesion factors in multiple myeloma and B-cell lymphoma, respectively (35, 36).

Taken together, MSC-interacting subpopulations showed unique regulatory patterns, focusing on either proliferation or adhesion.

### nMA-INA6 and MA-INA6 Show Increased Apoptosis Signaling Mediated by ER Stress, p53, and Death Domain Receptors

As stated previously, apoptosis rates increased in INA-6 cells grown on confluent hMSCs compared with CM-INA6 cells after 24 hours of coculture (Fig. 1D). Because this setup was similar to that used to separate hMSC-interacting subpopulations using WPSC, we looked for enrichment of apoptosis-related terms (Fig. 5B). “Regulation of cellular response to stress” and “intrinsic apoptotic signaling pathway (in response to ER stress)” are terms that were enriched in nMA *vs* CM, MA *vs* CM, and their overlap. We also found specific stressors for either nMA-INA6 (“intrinsic apoptotic signaling pathway by p53 class mediator”) or MA-INA6 (“extrinsic apoptotic signaling pathway via death domain receptor”). Therefore, apoptosis may be driven by ER stress in both nMA-INA6 and MA-INA6, but also by individual pathways such as p53 and death domain receptors, respectively.

### nMA-INA6 and MA-INA6 Regulate Genes Associated with Bone Loss

Myeloma cells cause bone loss by degradation and dysregulation of bone turnover via DKK1 and OPG (37–39). RNA-seq of hMSC-interacting subpopulations showed enrichment with functional terms “skeletal system development” and “ossification” (Fig. 5A; Supplementary Fig. S6), as well as the regulation of *MMP2*, *MMP14*, *DKK1,* and *OPG*. Validation by qPCR (Fig. 4C and D) showed that MA-INA6 significantly upregulated both *MMP14* and *MMP2* compared with either nMA-INA6 or CM-INA6. The expression of *DKK1*, however, was upregulated significantly in nMA-INA6 (and not significantly upregulated in MA-INA6), while *OPG* was significantly downregulated only in nMA-INA6.

Together, hMSC-interacting subpopulations might contribute to bone loss through different mechanisms: MA-INA6 expression of matrix metalloproteinases and nMA-INA6 via paracrine signaling.

### MA-INA6 Upregulate Collagen and Chemokines Associated with BM Retention

Retention of myeloma cells within the BM is mediated by adhesion to the ECM (e.g., collagen VI) and the secretion of chemokines (CXCL8 and CXCL12; refs. 7, 11), potentially counteracting dissemination. RNA-seq of hMSC-interacting subpopulations showed that genes upregulated in MA-INA6 were enriched with collagen biosynthesis and modifying enzymes, as well as chemotaxis and chemotaxis-related terms (Fig. 5B). Using qPCR, we validated the upregulation of collagen cross-linkers (*LOX* and *TGM2*), collagen-binding *DCN* and chemokines (*CXCL8* and *CXCL12*) in MA-INA6 compared with both nMA-INA6 and CM-INA6 (Fig. 4D). Therefore, MA-INA6 can provide both an adhesive surface and soluble signals for the retention of malignant plasma cells in the BM.

### nMA-INA6 Show Highest Viability During IL6 Withdrawal

Although RNA-seq did not reveal *IL6* induction in any WPSC-isolated subpopulation, nMA-INA6 upregulated *IGF-1* 1.35-fold [RNA-seq, nMA vs. (MA & CM)], which was shown to stimulate growth in CD45^+^ and IL6-dependent myeloma cell lines such as INA-6, implying increased autonomy for nMA-INA6 (40).

To test the autonomy of hMSC-interacting INA-6 subpopulations, we isolated them using WPSC after 24 and 48 hours of coculture, subcultured them for 48 hours under IL6 withdrawal, and measured both viability and apoptosis (Fig. 5D). Among the subpopulations, nMA-INA6 was the most viable. Compared with MA-INA6, nMA-INA6 increased cell viability by 8- or 4-fold when cocultured for 24 or 48 hours, respectively [Hedges g of log_10_(Fold Change) = 2.31 or 0.82]. However, the difference was no longer significant after 48 hours of coculture, probably because nMA-INA6 adhered to the hMSC layer (turning into MA-INA6) during prolonged coculture, which could also explain why the viability of MA-INA6 cell subcultures increased with prolonged coculture. Nevertheless, nMA-INA6 did not achieve the same viability as that of INA-6 cells cultured with IL6. Despite the differences in viability, subcultures of hMSC-interacting subpopulations did not show any differences in caspase 3/7 activity when cocultured for 48 hours (Fig. 5D, right).

Overall, among the hMSC-interacting subpopulations, nMA-INA6 had the highest chance of surviving IL6 withdrawal.

### Genes Upregulated by MA-INA6 are Associated with an Improved Disease Prognosis

**FIGURE 6:**
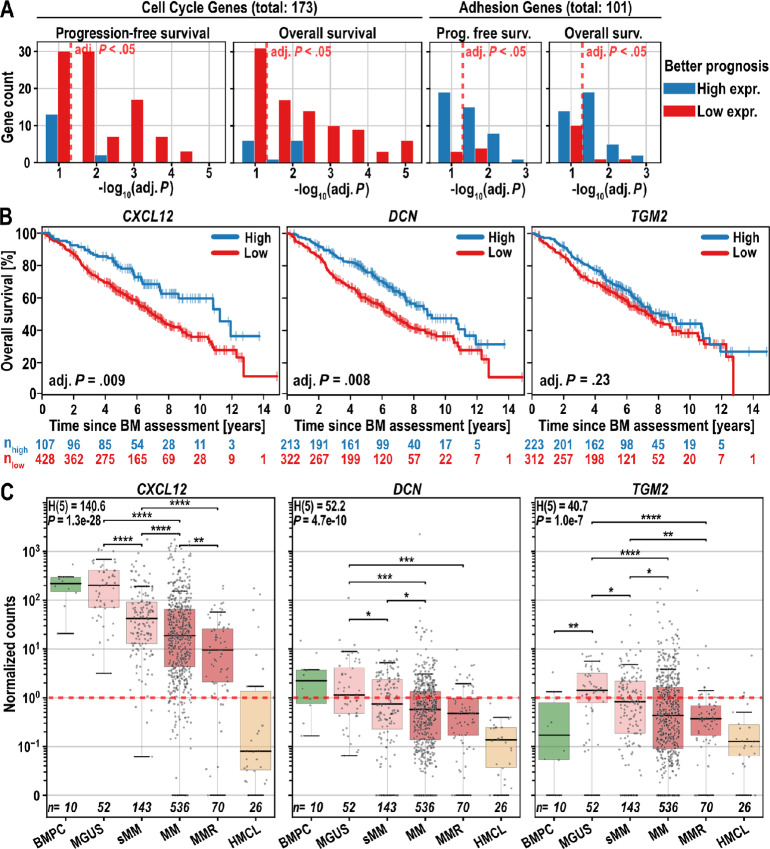
Survival of patients with multiple myeloma regarding the expression levels of adhesion and bone retention genes. **A,***P*-value distribution of genes associated with patient survival (*n* = 535) depending on high or low expression levels. Red dashed line marks the significance threshold of *P*-adj = 0.05. Histogram of *P* values was plotted using a bin width of −log_10_(0.05)/2. Patients with high and low gene expression were delineated using maximally selected rank statistics (maxstat). **B,** Survival curves for three genes taken from the list of adhesion genes shown in A, maxstat thresholds defining high and low expression were: *CXCL12*: 81.08; *DCN*: 0.75; *TGM2*: 0.66 normalized counts. **C,** Gene expression (RNA-seq, *n* = 873) measured in normalized counts (edgeR) of *CXCL12*, *DCN* in BMPC, MGUS, smoldering multiple myeloma (sMM), multiple myeloma (MM), multiple myeloma relapse (MMR), human myeloma cell lines (HMCL). The red dashed line marks one normalized read count. **Statistics** (A, B): log-rank test; (C): Kruskal–Wallis, Mann–Whitney U test. All *P* values were corrected using the Benjamini–Hochberg procedure.

To relate the adhesion of MA-INA6 observed *in vitro* to the progression of multiple myeloma, we assessed patient survival [*n* = 535, Seckinger and colleagues 2018 (21, 22)] depending on the expression level of 101 genes, which were upregulated in MA *vs* (nMA & CM) and are part of the ontology terms “extracellular matrix organization,” “ECM proteoglycans,” “cell-substrate adhesion,” and “negative regulation of cell-substrate adhesion” (Fig. 6A; Supplementary Table S2). As a reference, we generated a list of 173 cell cycle–related genes that were upregulated by nMA-INA6 versus (MA-INA6 & CM-INA6).

As expected, longer patient survival was associated with low expression of the majority of cell cycle genes [71 or 68 genes for progression-free survival (PFS) or overall survival (OS)]. Only a few cell cycle genes (two for PFS and seven for OS) were associated with survival when highly expressed. Intriguingly, adhesion genes showed an inverse pattern: a large group of adhesion genes (24 for PFS and 26 for OS) was significantly associated with improved survival when highly expressed, whereas only a few genes (two for PFS and four for OS) improved survival when expressed at low levels (Table 1). We concluded that the myeloma-dependent expression of adhesion factors determined in our *in vitro* study correlates with improved patient survival.

**TABLE 1:** Adhesion and ECM genes (shown in Fig. 6A) were filtered by their association with patient survival (*P*-adj. < 0.01) and were categorized as continuously downregulated during disease progression

					Association of expression with survival	
Regulation during disease progression	*Gene*	Ensemble ID	Progression-free/Overall survival	Better prognosis with high/low expression	(*P*-unc)	(*P*-adj)
**Not Downregulated (or overall low expression)**	***CCNE2***	**ENSG00000175305**	Overall	Low	5.34E-04	8.64E-03
	***MMP2***	**ENSG00000087245**	Prog. Free	High	2.29E-05	2.32E-03
	***OSMR***	**ENSG00000145623**	Prog. Free	High	5.67E-04	7.15E-03
**Continuously Downregulated (BMPC > MGUS > sMM > MM > MMR)**	***AXL***	**ENSG00000167601**	Overall	High	3.64E-05	1.84E-03
	***COL1A1***	**ENSG00000108821**	Prog. Free	High	3.03E-04	4.37E-03
			Overall	High	5.93E-04	8.64E-03
	***CXCL12***	**ENSG00000107562**	Prog. Free	High	1.16E-04	2.93E-03
			Overall	High	6.48E-04	8.64E-03
	***CYP1B1***	**ENSG00000138061**	Overall	High	6.84E-04	8.64E-03
	***DCN***	**ENSG00000011465**	Overall	High	2.47E-04	8.33E-03
	***LRP1***	**ENSG00000123384**	Overall	High	4.34E-04	8.64E-03
	***LTBP2***	**ENSG00000119681**	Prog. Free	High	9.03E-05	2.93E-03
	***MFAP5***	**ENSG00000197614**	Prog. Free	High	2.43E-04	4.09E-03
	***MMP14***	**ENSG00000157227**	Prog. Free	High	6.93E-05	2.93E-03
	***MYL9***	**ENSG00000101335**	Prog. Free	High	1.46E-04	2.95E-03
			Overall	High	1.56E-05	1.57E-03

NOTE: The complete list is presented in Supplementary Table S2.

Abbreviations: BMPC, bone marrow plasma cells; MGUS, monoclonal gammopathy of undetermined significance; sMM, smoldering multiple meloma; MM< multiple myeloma; and MMR, multiple myeloma relapse; *P*-unc, unadjusted *P* values; *P*-adj, *P* values adjusted using the Benjamini–Hochberg method with 101 genes.

### Expression of Adhesion- or Retention-related Genes (CXCL12, DCN, and TGM2) is Decreased During Progression of Multiple Myeloma

To examine how the disease stage affects the adhesion and BM retention of myeloma cells *in vitro*, we analyzed the expression of *CXCL12* in healthy plasma cell [bone marrow plasma cell (BMPC)] cohorts of patients at different disease stages and in myeloma cell lines (HMCL) [described in Seckinger and colleagues 2018 (22)] (Fig. 6C). We also included *DCN* and *TGM2* because both are suggested to inhibit metastasis in different cancers by promoting cell–matrix interactions (8, 41). In accordance with independent reports (9, 42), high expression of *CXCL12* and *DCN* by myeloma cells was associated with improved OS (adj. *P* = 0.009 and 0.008, respectively; Fig. 6B).

*CXCL12* is expressed by BMPCs (median = 219 normalized counts), but its expression levels are significantly lower from monoclonal gammopathy of undetermined significance (MGUS) to relapsed multiple myeloma (MMR; median = 9 normalized counts in MMR and absent expression in most HMCL). *DCN* (but not *TGM2*) was weakly expressed in BMPCs (Q_1_ = 0.7, Q_3_ = 3.7, normalized counts), whereas *TGM2* was weakly expressed only in patients with MGUS (Q_1_ = 0.4, Q_3_ = 4.1 normalized counts). The median and upper quartiles of both *DCN* and *TGM2* decreased continuously after each stage, ending at Q_3_ = 0.9 and Q_3_ = 0.6, respectively, in MMR. A total of 49 of the 101 adhesion genes (Fig. 6A) followed a similar pattern of continuous downregulation in the advanced stages of multiple myeloma (Supplementary Fig. S7 and S8), of which 19 genes were associated with longer PFS when they were highly expressed. The other 52 (out of 101) adhesion genes that were not downregulated across disease progression (or were expressed at a level too low to make that categorization) contained only five genes that were associated with longer PFS at high expression (Table 1; Supplementary Table S2).

Together, the expression of adhesion or BM retention–related markers (*CXCL12*, *DCN,* and *TGM2)* is reduced or lost at advanced stages of multiple myeloma, which could enhance dissemination and reduce retention in the BM microenvironment.

## Discussion

**FIGURE 7:**
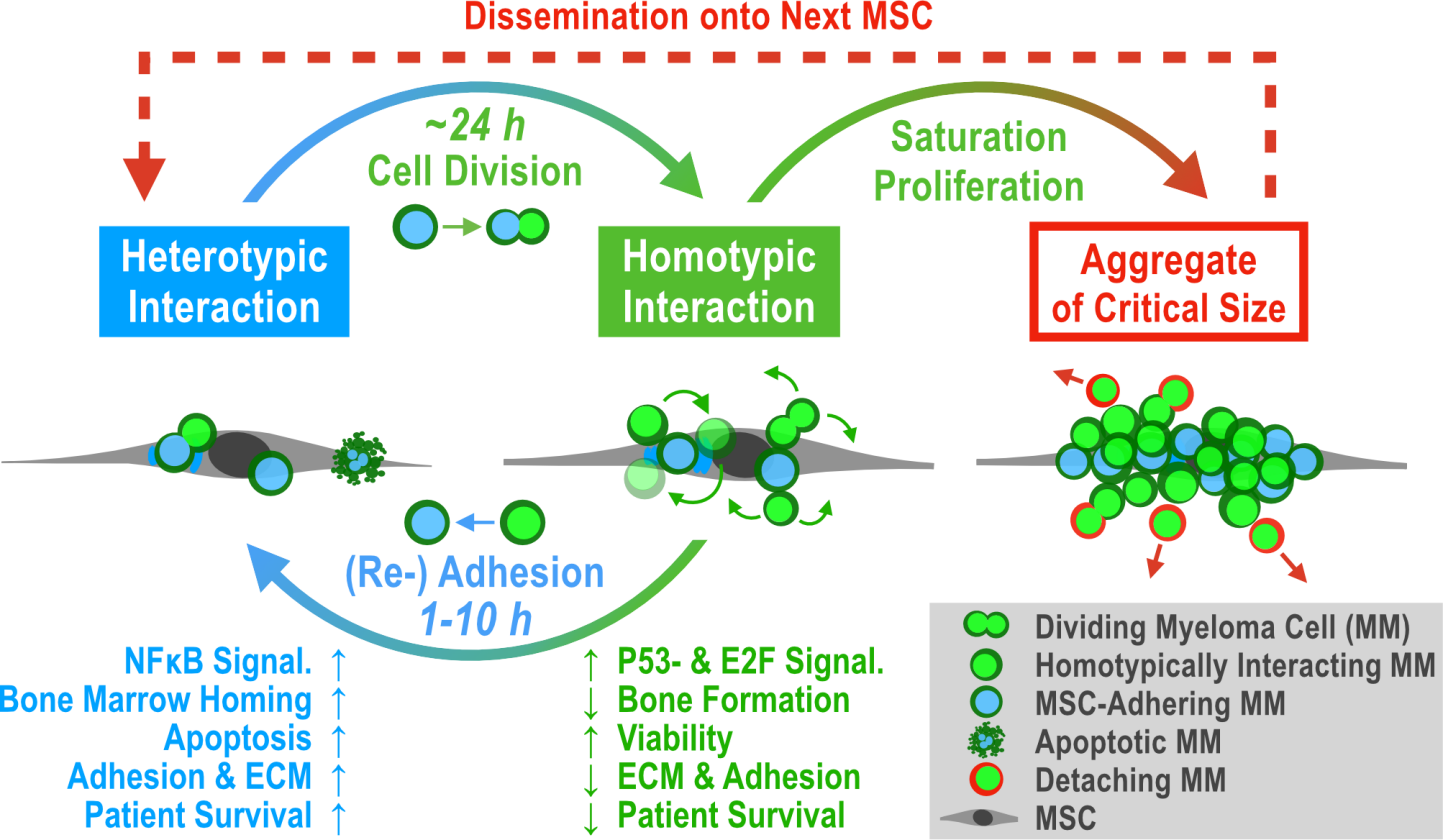
Proposed model of “Detached Daughter Driven Dissemination” in aggregating multiple myeloma. **Heterotypic Interaction:** Malignant plasma cells colonize the BM microenvironment by adhering to an MSC (or osteoblast, ECM, etc.) to maximize growth and survival through paracrine and adhesion-mediated signaling, even if contact may trigger initial apoptosis. Gene expression will focus on establishing a strong anchor within the BM, but also on attracting other myeloma cells (via secretion of ECM factors and CXCL12/CXCL8, respectively). **Cell Division:** Cell fission can generate one daughter cell that no longer adheres to the MSC (nMA). **Homotypic Interaction:** If myeloma cells have the capacity to grow as aggregates, the daughter cell stays attached to their MSC-adhering mother cell (MA). **Re-Adhesion:** The daughter cell “rolls around” the mother cell until it readheres to the MSC. Our model estimates the rolling duration to be 1–10 hours long. **Proliferation and Saturation:** We estimate that a single myeloma cell covers one MSC completely after roughly four population doublings. When heterotypic adhesion is saturated, subsequent daughter cells benefit from a homotypic interaction, because they stay close to growth factor–secreting MSCs and focus gene expression on proliferation (e.g., driven by E2F) and not adhesion (driven by NFκB). **Critical Size:** Homotypic interaction is weaker than heterotypic interaction, and each cell fission destabilizes the aggregate. Hence, detachment of myeloma cells may depend mostly on aggregate size. **Dissemination:** After myeloma cells have detached, they gained a viability advantage through IL6 independence (with unknown duration), which enhances their survival outside of the BM and allows them to spread throughout the body.

In this study, we developed an *in vitro* model to investigate the attachment/detachment dynamics of INA-6 cells to/from hMSCs and established methods to isolate the attached and detached intermediates nMA-INA6 and MA-INA6. Second, we characterized a cycle of (re)attachment, division, and detachment, linking cell division to the switch that causes myeloma cells to detach from hMSC adhesion (Fig. 7). Third, we identified clinically relevant genes associated with patient survival, in which better or worse survival was based on the adherence status of INA-6 to hMSCs.

INA-6 cells emerged as a robust choice for studying myeloma dissemination *in vitro*, showing rapid and strong adherence, as well as aggregation exceeding MSC saturation. The IL6 dependency of INA-6 enhanced the resemblance of myeloma cell lines to patient samples, with INA-6 ranking 13th among 66 cell lines (43). Despite variations in BM MSCs between multiple myeloma and healthy states, we anticipated the robustness of our results, given the persistent strong adherence and growth signaling from MSCs to INA-6 during cocultures (44).

We acknowledge that INA-6 cells alone cannot fully represent the complexity of myeloma aggregation and detachment dynamics. However, the diverse adhesive properties of myeloma cell lines pose a challenge. We reasoned that attempting to capture this complexity within a single publication would not be possible. Our focus on INA-6 interactions with hMSCs allowed for a detailed exploration of the observed phenomena, such as the unique aggregation capabilities that facilitate the easy detection of detaching cells *in vitro*. The validity of our data was demonstrated by matching the *in vitro* findings with the gene expression and survival data of the patients (e.g., *CXCL12*, *DCN*, and *TGM2* expression, *n* = 873), ensuring biological consistency and generalizability regardless of the cell line used.

The protocols presented in this study offer a cost-efficient and convenient solution, making them potentially valuable for a broader study of cell interactions. We encourage optimizations to meet the varied adhesive properties of the samples, such as decreasing the number of washing steps if the adhesive strength is low. We caution against strategies that average over multiple cell lines without prior understanding their diverse attachment/detachment dynamics, such as homotypic aggregation. Such detailed insights may prove instrumental when considering the diversity of myeloma patient samples across different disease stages (33, 34).

The intermediates, nMA-INA6 and MA-INA6, were distinct but shared similarities in response to cell stress, intrinsic apoptosis, and regulation by p53. Unique regulatory patterns were related to central transcription factors: E2F1 for nMA-INA6; and NFκB, SRF, and JUN for MA-INA6. This distinction may have been established through antagonism between p53 and the NFκB subunit RELA/p65 (45, 46). Similar regulatory patterns were found in transwell experiments with RPMI1-8226 myeloma cells, where direct contact with the MSC cell line HS5 led to NFκB signaling and soluble factors to E2F signaling (20).

The first subpopulation, nMA-INA6, represented proliferative and disseminative cells: They drove detachment through cell division, which was regulated by E2F, p53, and likely their cross-talk (47). nMA-INA6 upregulate cell cycle progression genes associated with worse prognosis, because proliferation is a general risk factor for an aggressive disease course (48). In addition, nMA-INA6 survived IL6 withdrawal better than CM-INA6 and MA-INA6, implying their ability to proliferate independently of the BM (1). Indeed, xenografted INA-6 cells developed autocrine IL6 signaling but remained IL6-dependent after explantation (23). The increased autonomy of nMA-INA6 cells can be explained by the upregulation of *IGF-1*, being the major growth factor for myeloma cell lines (40). Other reports characterized disseminating cells differently: Unlike nMA-INA6, circulating myeloma tumor cells were reported to be nonproliferative and BM retentive (49). In contrast to circulating myeloma tumor cells, nMA-INA6 were isolated shortly after detachment and therefore these cells are not representative of further steps of dissemination, such as intravasation, circulation, or intravascular arrest (3). Furthermore, Brandl and colleagues described proliferative and disseminative myeloma cells as separate entities, depending on the surface expression of CD138 or JAM-C (4, 50). Although CD138 was not differentially regulated in nMA-INA6 or MA-INA6, both subpopulations upregulated JAM-C, indicating disease progression (50).

Furthermore, nMA-INA6 showed that cell division directly contributed to dissemination. This was because INA-6 daughter cells emerged from the mother cell with distance to the hMSC plane in the two-dimensional setup. A similar mechanism was described in an intravasation model in which tumor cells disrupt the vessel endothelium through cell division and detach into blood circulation (51). Overall, cell division offers key mechanistic insights into dissemination and metastasis.

The other subpopulation, MA-INA6, represented cells retained in the BM; MA-INA6 strongly adhered to MSCs, showed NFκB signaling, and upregulated several retention, adhesion, and ECM factors. The production of ECM-associated factors has recently been described in MM.1S and RPMI-8226 myeloma cells (52). Another report did not identify the upregulation of such factors after direct contact with the MSC cell line HS5; hence, primary hMSCs may be crucial for studying myeloma–MSC interactions (20). Moreover, MA-INA6 upregulated adhesion genes associated with prolonged patient survival and showed decreased expression in relapsed myeloma. As myeloma progression implies the independence of myeloma cells from the BM (1, 43), we interpreted these adhesion genes as mediators of BM retention, decreasing the risk for dissemination and thereby potentially prolonging patient survival. However, the overall impact of cell adhesion and ECM on patient survival remains unclear. Several adhesion factors have been proposed as potential therapeutic targets (50, 53). Recent studies have described the prognostic value of multiple ECM genes, such as those driven by NOTCH (52). Another study focused on ECM gene families, of which only six of the 26 genes overlapped with our gene set (Supplementary Table S2; ref. 54). The expression of only one gene (*COL4A1*) showed a different association with OS than that in our cohort. The lack of overlap and differences can be explained by dissimilar definitions of gene sets (homology *vs* gene ontology), methodologic discrepancies, and cohort composition.

In summary, our *in vitro* model provides a starting point for understanding the initiation of dissemination and its implications for patient survival, providing innovative methods, mechanistic insights into attachment/detachment, and a set of clinically relevant genes that play a role in BM retention. These results and methods might prove useful when facing the heterogeneity of disseminative behaviors among myeloma cell lines and primary materials.

## Supplementary Material

Supplementary Methods Supplementary Methods

Supplementary Table 1 Supplementary Table 1

Supplementary Table 2 Supplementary Table 2

Supplementary Table 3 Supplementary Table 3

Supplementary Video 1 Supplementary Video 1

Supplementary Video 2 Supplementary Video 2

Supplementary Video 3 Supplementary Video 3

Supplementary Video 4 Supplementary Video 4

Supplementary Figures 1-8 Supplementary Figure 1. Principle and quantification of the V-well adhesion assay of fluorescently labeled myeloma cells adapted by Weetall et al. 2001.
Supplementary Figure 2. Validation of image cytometric analysis of cell cycle in four INA-6 cultures.
Supplementary Figure 3. Cell cycle analysis of INA-6 pellets gained from V-Well Adhesion assay (Fig. 3).
Supplementary Figure 4. Representative (one of the four independent sample sets as seen in Supplementary Figure 3) curve fitting analysis of cell cycle profiles generated by Image Cytometry.
Supplementary Figure 5. Correlation of RNAseq with qPCR Left: Validation of RNAseq results (Fig. 4) with qPCR showing the log2(foldchange expression) of 18 genes.
Supplementary Figure 6. Functional enrichment analysis by Metascape using genes that are differentially expressed between MSC-interacting subpopulations.
Supplementary Figure 7. Expression levels of adhesion genes that are downregulated and associated with survival (p < 0.01). Bone Marrow Plasma Cell (BMPC), Monoclonal Gammopathy of Undetermined Significance (MGUS), Smoldering Multiple Myeloma (sMM), Multiple Myeloma (MM), Multiple Myeloma Relapse (MMR).
Supplementary Figure 8. Expression levels of adhesion genes that are not downregulated and associated with survival (p < 0.01).
