# Supplementary material for: Modeling Myeloma Dissemination In Vitro with hMSC-interacting Subpopulations of INA-6 Cells and Their Aggregation/Detachment Dynamics
Source: Cancer Res Commun. 2024 Apr 29;4(4):1150–64. doi: 10.1158/2767-9764.CRC-23-0411 (PMC11057410; doi:10.1158/2767-9764.CRC-23-0411)
Supplement: Supplementary Methods [file crc-23-0411-s01.docx]

Supplementary Methods

Modelling Myeloma Dissemination *in vitro* with hMSC-Interacting Subpopulations of INA-6 Cells and their Aggregation/Detachment Dynamics

Martin Kuric^1^, Susanne Beck^2^, Doris Schneider^1^, Wyonna Rindt^3^, Marietheres Evers^4^, Jutta Meißner-Weigl^1^, Sabine Zeck^1^, Melanie Krug^1^, Marietta Herrmann^5^, Tanja Nicole Hartmann^6^, Ellen Leich^4^, Maximilian Rudert^7^, Denitsa Docheva^1^, Anja Seckinger^8^, Dirk Hose^8^, Franziska Jundt^3^, Regina Ebert^1^

^1^University of Würzburg, Department of Musculoskeletal Tissue Regeneration, Würzburg, Germany

^2^University Hospital Heidelberg, Institute of Pathology, Heidelberg, Germany

^3^University Hospital Würzburg, Department of Internal Medicine II, Würzburg, Germany

^4^University of Würzburg, Institute of Pathology, Comprehensive Cancer Center Mainfranken, Würzburg, Germany

^5^University Hospital Würzburg, IZKF Research Group Tissue Regeneration in Musculoskeletal Diseases, Würzburg, Germany

^6^University of Freiburg, Department of Internal Medicine I, Faculty of Medicine and Medical Center, Freiburg, Germany

^7^University of Würzburg, Orthopedic Department, Clinic König-Ludwig-Haus, Würzburg, Germany

^8^Vrije Universiteit Brussel, Department of Hematology and Immunology, Jette, Belgium

### Isolation and Culturing of Primary Human Bone Marrow-Derived Mesenchymal Stromal Cells

Primary human Mesenchymal Stromal Cells (MSCs) were obtained from the femoral head of patients (Supplementary Table 1) undergoing elective hip arthroplasty. Material was collected with written informed consent of all patients and the procedure was approved by the local Ethics Committee of the University of Würzburg (186/18). In brief, bone marrow was washed with MSC-medium (Dulbecco’s modified Eagle’s medium (DMEM/F12, Thermo Fisher Scientific, Darmstadt, Germany) supplemented with 10% Fetal Calf Serum (FCS, Bio&Sell GmbH, Feucht, Germany, Fernandez-Rebollo et al., 2017), 100 U/ml penicillin, 0.1 mg/ml streptomycin (Thermo Fisher Scientific), 50 µg/ml ascorbate and 100 nmol/l sodium selenite (both Sigma-Aldrich GmbH, Munich, Germany) and centrifuged at 250 *g* for 5 min. The pellet was washed four times with MSC-medium and resulting supernatants containing released cells were collected. Cells were pelleted and cultured at a density of 1 x 10^9^ cells per 175 cm^2^ culture flask. After two days non-attached cells were washed away and adherent ones were cultivated in MSC-medium until confluence. Then, they were either frozen in liquid nitrogen or directly utilized for experiments. hMSC cultures were sustained for a maximum of two passages. All cells were cultured at 37 °C and at 5% CO_2_.

### Culturing of Myeloma Cell Lines

The plasmacytoma cell line INA-6 [RRID:CVCL_5209; DSMZ, Braunschweig, Germany, authenticated by DSMZ in 2014 (Burger et al., 2001; Gramatzki et al., 1994) was cultivated in RPMI1640 medium (Thermo Fisher Scientific) supplemented with 20 % (v/v) FCS, 100 µg/ml gentamicin, 2 mmol/l L-glutamine (both Thermo Fisher Scientific), 1 mmol/l sodium pyruvate, 100 nmol/l sodium selenite (both Sigma Aldrich GmbH) and 2 ng/ml recombinant human interleukin-6 (IL-6; Miltenyi Biotec, Bergisch Gladbach, Germany). INA-6 were passaged three times per week by diluting them to 1 x 10^5^, 2 x 10^5^, or 4 x 10^5^ cells/mL for 3, 2 and 1 days of culturing, respectively. MM.1S (RRID:CVCL_8792) (Greenstein et al., 2003), and U266 cells (CVCL_0566), (Nilsson et al., 1970) were propagated and cultivated in RPMI1640 medium comprising 10 % (v/v) FCS, 100 U/ml penicillin, 100 µg/ml streptomycin, 2 mmol/l L-glutamine, and 1 mmol/l sodium pyruvate. All cells were cultured at 37 °C and at 5% CO_2_.

### Co-Culturing of Primary hMSCs and INA-6 and MSC-Conditioning of Medium

For each co-culture, hMSCs were seeded out 24 h prior to INA-6 addition to generate MSC-conditioned medium (CM). CM from different donors was collected separately and used immediately when adding INA-6. To ensure that CM was free of hMSCs, it was strained (40 µm) and centrifuged for 15 minutes at 250 *g*. INA-6 cells were washed with PBS (5 min, 1200 rpm), resuspended in MSC-medium and added to hMSCs such that co-culture comprised 33% (v/v) of CM gathered directly from the respective hMSC-donor. Co-cultures did not contain IL-6 (Chatterjee et al., 2002).

### Collagen I Coating

Collagen I solution (isolated from rat tail, Corning, NY, USA) was diluted 1:2 (75 ng/mL) in acetic acid (0.02 N), applied to 96-well plates (30 µL in each well) and incubated for 2 h at room temperature. Acetic acid was removed and wells were washed once with 100 µL of PBS. Coated plates were stored dry at 4 °C.

### Fluorescent Staining of Cells

For each live staining, cells were strained (70 µm) to remove clumps and washed (5 min, 250 *g*) once with the respective media (without FCS) and then resuspended in staining reagents.

For CellTracker™ Green CMFDA Dye and CellTracker™ Deep Red Dye (Thermo Fisher Scientific) staining, 1 mL staining solution for a maximum of 1 x 10^6^ cells was prepared. Staining was done at room temperature (RT) for 15 min using 5 µM CMFDA (5-Chlormethyl-fluoresceindiacetat) and 5 min of 1-2 µM DeepRed. To reduce background, stained cells were pelleted, resuspended in cell medium (containing FCS), incubated for 30 min (37 °C, 5% CO_2_), washed in cell medium, resuspended in 100 µL - 1 mL and counted.

For PKH26 staining (Sigma Aldrich GmbH), a maximum of 1 x 10^4^ cells was resuspended in 500 µL diluent C before swiftly adding 500 µL of staining solution (1 µL diluted in 500 µL diluent C) and incubating cells for 5 min at RT. The staining reaction was stopped by adding 1 mL of FCS-containing medium and adding 3 mL of FCS-free medium. Cells were washed with 10 mL of FCS-containing medium, resuspended in 100 µL - 1 mL cell medium, and counted.

For Calcein-AM (Calcein-*O*,*O*′-diacetat-tetrakis-(acetoxymethyl)-ester) (Thermo Fisher Scientific) staining, end concentrations of 0.5 µM were used. 12.5 µL of diluted stock solution (2.5 µM) was carefully added to 50 µL of the co-culture and incubated for 10 minutes at 37 °C.

For Hoechst 33342 staining, cells were washed once with PBS, resuspended in a maximum of 500 µL of PBS, and fixed with 5 mL of ice-cold ethanol (70% v/v) by vigorously pipetting up and down to dissociate aggregates. Cells were washed once with PBS and stained with 2.5 µg/mL Hoechst 33342 (Thermo Fisher Scientific) diluted in PBS for 1 h at 37 °C.

### Automated Fluorescence Microscopy

To remove clumps for microscopic applications, we cultured cells in 40 µm strained medium containing FCS. To reduce background fluorescence and phototoxicity, we used phenol-red free versions of the respective medium, if available.

All microscopy equipment was acquired from ZEISS. The microscope was an Axio Observer 7 with confocal ApoTome.2 equipped with a motorized reflector revolver and motorized scanning table (130x100 mm). The microscope was mounted on an Antivibrations-Set (Axio Observer (D)) with two antivibration carrier plates, each equipped with two vibration dampening feet. The light source was a microLED 2 for transmission light and (for fluorescence) Colibri 7 (R[G/Y]B-UV) for five channels of incident light (385, 475, 555, 590, 630 nm). For excitation (EX) and emission (EM) light filtering and beam splitting (BS) we used the following reflectors: 96 HE BFP shift free (E) (EX: 390/40, BS: 420, EM: 450/40), 43 HE Cy 3 shift free (E) (EX: 550/25, BS: 570, EM: 605/70), 38 HE eGFP shift free (E) (EX: 470/40, BS: 495, EM: 525/50) and 90 HE LED (E) (EX: 385, 475, 555 und 630 nm, BS: 405 + 493 + 575 + 653, EM: 425/30 + 514/30 + 592/30 + 709/100). We used the black and white camera Axiocam 506 mono (D) and if not stated otherwise, 2x2 binning was used for fluorescence imaging. For mosaic acquisitions (“tiles”) we used a tiling overlap of 8-10% and image tiles were not stitched. Images were magnified 5x and 10x (Fluar 5x/0.25 M27 and EC Plan-Neofluar 10x/0.3 Ph1 M27).

### Cell Viability and Apoptosis Assay

To examine cell viability and apoptosis, cells were seeded in a 96-well plate (1 x 10^4^ cells per well) to be measured inside culture well after respective incubation time immediately. ATP-amount and Caspase 3/7 activity were used as a proxy for viability and apoptosis rates, respectively. They were assessed using the CellTiter-Glo Luminescent Cell Viability Assay and the Caspase-Glo 3/7 Assay, respectively (Promega GmbH, Mannheim, Germany), according to the manufacturer’s instructions. Luminescence was measured with an Orion II Luminometer (Berthold Detection Systems, Pforzheim, Germany).

### Microscopic Characterization of hMSC Saturation

For saturating hMSC with INA-6, hMSCs were stained with CellTracker Green, plated out on 384-well plates (Greiner Bio-One GmbH, Frickenhausen, Germany) at 5 x 10^3^ hMSC/cm² and cultured for 24 h. INA-6 cells were stained with CellTracker DeepRed, resuspended in MSC-medium, added to adhering hMSCs in different amounts (5 x 10^3^, 1 x 10^3^, 2 x 10^3^ INA-6/cm²) and co-cultured for 24 h and 48 h. The complete co-culture was scanned and the number of INA-6 cells adhering on one hMSC was counted manually for 100 MSCs for each technical replicate. Fluorescent images were digitally re-stained (INA-6 green, hMSC inverse black).

**Analysis INA-6 Survival and Aggregation Depending on hMSCs Confluence**

To describe aggregate growth and survival of INA-6 depending on hMSC density, unstained hMSCs were seeded out into 96-well plates (white, clear bottom, Greiner) at different densities (see Seeding Table). To ensure nutrient supply, we used lower cell densities for longer co-culturing durations while maintaining constant ratios of INA-6 to adhesion surface provided by hMSCs. Those plates that were to be assessed after 72 h of co-culturing received further 100 µl of fresh MSC-medium after 24 h of co-culturing (total volume of 300 µL), and after 48 h of co-culturing, 100 µL was removed gently from the co-culture and (carefully not to stir up co-culture on bottom) replaced with fresh MSC-medium after 48 h of co-culturing.

To describe aggregate growth, complete wells were scanned using 10x magnification, phase contrast, 2x2 binning, and autofocus focusing on each tile both before and after harvesting. Afterwards, INA-6 cells were harvested for measuring viability and apoptosis.

##### **Seeding Table:** Seeding densities for describing growth and survival of INA-6 depending on hMSC density. Co-cult. dur. = Co culturing duration; MSC-adh. surface = adhesion surface provided by hMSCs; vol. = volume.

| **Co- cult. dur. [h]** | **hMSC density  [1000 hMSC/cm²]** | | | **INA-6 density [1000 INA-6/cm²]** | **Ratios INA : MSC (adh. surface)** | | | **Seeding vol. [µL]** | **End  vol. [µL]** |
| --- | --- | --- | --- | --- | --- | --- | --- | --- | --- |
| **24** | 2 | 10 | 40 | 10 | 1 : 0.2 | 1 : 1 | 1 : confluent | 200 | 200 |
| **48** | 1 | 5 | 40 | 5 | 1 : 0.2 | 1 : 1 | 1 : confluent | 200 | 200 |
| **72** | 1 | 5 | 40 | 5 | 1 : 0.2 | 1 : 1 | 1 : confluent | 200 | 300  [after 24 h: + 100]  [after 48 h: exchange 100] |

For luminescent assessment of cell survival, INA-6 were harvested by removing co-culture medium, adding 150 µL of MSC-medium, and then stirred by strongly pipetting up and down twice while aiming the pipette tip at the upper corner, lower left and lower right of the well bottom (‘Mercedes star”). Washing and stirring was repeated once before washing wells again with 150 mL MSC-medium. Harvested INA-6 cells were strained (40 µm filter), pelleted, and resuspended in 200 µL MSC-medium. Cells were counted using Neubauer chambers, re-distributed into 96-well plates (white, clear bottom) with 1 x 10^5^ INA-6 cells per well, and then subjected to viability and apoptosis assays.

To minimize the loss of sensitive apoptotic cells, another approach was used to measure viability and apoptosis without harvesting INA-6 cells. hMSCs and INA-6 were seeded out individually in parallel to the co-cultures (Supplementary Table 2). Prior to measuring viability and apoptosis, culture volume was adjusted to 150 µL by removing 50 µL or 150 µL for the timepoints 48 h or 72 h, respectively (carefully not to stir up culture on bottom). 100 µL of luminescent reagents were then added directly to 150 µL of co-culture. The fold change of viability or apoptosis that is due to MSC interaction ($FC_{MSC interaction}$) was then calculated using the following formula, with $L$ being the mean of four technical replicates measured in relative luminescent units per seconds [RLU/s], $L_{Co Culture}L_{MSC}$, $L_{INA 6}$ the luminescence measured in the co-culture, hMSCs alone and INA 6 alone, respectively.

$FC_{MSC Interaction}=\frac{L_{Co Culture}}{L_{MSC} + L_{INA ­6}}$

### Time-Lapse Characterization of INA-6 Aggregation, Detachment and Division

In order to record the aggregation and detachment of INA-6 in contact with hMSCs, hMSCs (5 x10^3^ cells/cm²) were fluorescently stained with PKH26 and plated onto 8-well µ-Slides (ibidi, Gräfelfing, Germany). hMSCs were incubated for 24 h before being placed into an ibidi Stage Top Incubation System and were equilibrated to the incubation system for a minimum of 3 h (80% humidity and 5% CO_2_). INA-6 cells (2 x 10^4^ cells/cm²) were washed and resuspended in 33% (v/v) MSC-conditioned medium before adding them directly before acquisition start in a small volume (10 µL). Brightfield and fluorescence images of 13 mm² of co-culture were acquired every 15 minutes for 63 h. Movement speed of the motorized table was adjusted to the lowest setting that allows acquisition of the complete region within 15 minutes.

Respective events of interest were analyzed manually and categorized into defined event parameters. Events were binned across the time axis using these boundaries: [0.0, 12.85, 25.7, 38.55, 51.4, 64.25]. We collected a minimum of events per recording and analysis so that each time bin contained at least 5 values, except when analyzing detachment events, since these did not appear before 20 h of incubation for some replicates. For each recording and event parameter, the event count was normalized by dividing by the total number of events per time bin.

We determined the frequency and the cause of aggregation by looking for two interacting INA-6 cells and went backward in time to see if they were two daughter cells or if two independent INA-6 cells had collided.

We determined the frequency of aggregates with detaching cells by tracing their growth across the complete time-lapse and looking for detachment events. We picked random 100 aggregates by including aggregates from both the border and center of the well.

We characterized detachment events by noting multiple parameters manually: Time point of detachment, aggregate size (at the time of detachment), the last interaction partner, and the number of detaching INA-6 cells.

For characterizing cell division events, we recorded a new set of time-lapse videos using unstained hMSCs that were grown to confluence for 24 h (4 x 10^4^ hMSCs/cm²) to provide for unlimited adhesion surface. We categorized daughter cells in terms of their mobility (mobility being the speed of putative movements or “rolling”). The mobility criteria were met if one INA-6 daughter cell moved farther than half a cell radius within one frame (15 min) relative to the MSC-adherent INA-6 cell which was required to stand still in-between respective frames. We measured the “rolling” duration by subtracting the time point of the last perceived movement from the time point of division. We excluded those division events from the measurement of rolling duration, if INA-6 cells underwent apoptosis shortly after division.

### Cell Cycle Synchronization at M-Phase

INA-6 cells were arrested at mitosis by double thymidine (2 mM) treatments followed by 5 h of nocodazole (500 ng/mL) incubation. In detail: 3 x 10^5^/mL INA-6 in 4 mL were treated with 2 mM thymidine (Sigma Aldrich GmbH) for 16.5 h. Cells were released by washing them in INA-6 medium once and allowed to cycle for 9 h before treating them with 2 mM thymidine for 18 h a second time. Afterwards, cells were released and allowed to cycle for 2 h before treating them with 100 ng/ mL nocodazole (Sigma Aldrich GmbH) for 5 h. Arrested INA-6 were released by washing them once and resuspending them in MSC-medium with 33% MSC-conditioned medium. Cell cycle profile was checked using image cytometry (Supplementary Figure 2).

### V-Well Adhesion Assay

This assay was modified from (Weetall et al., 2001). 96 v-well plates were coated with collagen I (rat tail, Corning). Collagen coating ensures that confluent hMSCs withstand centrifugation even after hMSCs in the well tip were removed. hMSCs (4 x 10^4^ cells/cm^2^) were seeded out and grown to confluence for 24 h in collagen-coated v-well plates. To ensure that only INA-6 are pelleted in the v-well tip, hMSCs were removed from the well-tip by touching the well-ground with a 10 µL pipette and roughly pipetting hMSCs away.

Arrested INA-6 (1 x 10^4^ cells/cm²) were released by washing them once in PBS and resuspending them in 33% (v/v) MSC-conditioned medium before adding them on top of confluent hMSCs. INA-6 adhered for 1, 2, 3 and 24 h before the complete co-culture was stained with 0.5 µM Calcein-AM (10 min at 37 °C).

Non-adherent INA-6 were pelleted by centrifugation using a Hettich 1460 rotor (r = 124 mm) at 2000 rpm (555 *g*) for 10 min.

The well tip was imaged by fluorescence microscopy with 5x magnification, 96 HE emission filter, autofocus configured for maximum signal intensity, 2x2 binning and 14 bit grayscale depth. Pellet brightness was analyzed in ZEN 2.6 (Zeiss) by summing up pixel brightnesses across the complete pellet image. Background brightness was acquired from a cell culture with only hMSCs. Reference brightness was acquired from a cell culture with only INA-6, defining 100% pellet brightness without adhesion. Background intensity was subtracted before normalizing by reference. Outliers were removed from technical replicates (n=4) if their z-score was larger than 1.5 *σ* technical variation.

After measuring pellet brightnesses, the cell pellet was removed by pipetting 10 µL from the well tip. Pellets of the same technical replicates were pooled, washed in PBS, resuspended in 200 µL PBS, added to 1.8 mL ice-cold 70% ethanol and stored at -20 °C.

Remaining non-MSC-adhering INA-6 cells were removed by replacing culture medium with 100 µL of medium. MSC-adherent INA-6 were manually detached by rapid pipetting and equally pelleted, analyzed, and isolated.

### Cell Cycle Profiling

INA-6 cells were fixed in 70% ice-cold ethanol, washed, resuspended in PBS, distributed in 96-well plates and stained with Hoechst 33342 (2.5 µg/mL in PBS) for 1 h at 37 °C.

For image cytometric cell cycle profiling, plates were scanned completely using automated fluorescence microscopy with 5x magnification, 96 HE emission filter, 1x1 binning, 14 bit depth and an illumination time that fills 70% of grayscale range. The autofocus was configured to re-adjust every second tile. A pre-trained convolutional neural network (“DeepFeatures 2 reduced”, Intellesis, Zeiss) was fine-tuned to segment scans into background, single nuclei and fragmented nuclei. Nuclei were filtered to exclude fragmented nuclei and those nuclei with extreme size (within the range of 50-500 µm^2^) and roundness (within the range of 0.4-1.0). Cell cycle profiles were normalized by the mode of the nucleus intensities within the G0/G1 peak. To retrieve frequencies of cells cycling in G0/G1, S, and G2 phase, the brightness distribution of all single nuclei was fitted to the sum of three Gaussian curves (“Skewed Gaussian Model” for G0G1 and G2 phase, and “Rectangle Model” for S phase) using the python package LMFIT (Newville et al., 2014) (Supplementary Figure 4). The Gaussian curves were used to calculate the cell frequencies for each cell cycle phase by integration using the composite trapezoidal rule implemented by numpy.trapz (Harris et al., 2020).

For validation of image cytometry, 5 mL of INA-6 stock culture was removed and ethanol fixed as described above. Flow cytometry analyses were performed using an Attune Nxt Flow Cytometer (Thermo Fisher Scientific). Data analyses were performed using FlowJo V10 software (TreeStar, USA).

### Protocol: Well Plate Sandwich Centrifugation (WPSC)

96-well plates (flat bottom, clear) were coated with collagen I (rat tail, Corning). Collagen coating ensures that confluent hMSCs withstand centrifugation and repeated washing. hMSCs (2 x 10^4^ cells/cm^2^) were seeded out and grown to confluence for 72 h in collagen-coated 96-well plates.

To remove aggregates from the medium and prevent clogging of magnetic columns, we strained any FCS-containing fluid with a 40 µm cell strainer.

Collect MSC-conditioned medium and add INA-6:

1. Collect hMSC-conditioned medium (CM) from the well plates and replace it with 100 µL of fresh hMSC medium. Collect CM from different donors separately.
2. Strain CM (40 µm) and centrifuge it for 15 minutes at 250 *g* to ensure that CM does not contain hMSCs.
3. Dilute CM by mixing 2 parts of CM with 1 part of MSC-medium (dilute 1.5 fold).
4. Count INA-6 cells and retrieve enough cells to fill all 96 wells with 2 x 10^4^ INA-6/cm^2^ (6.8 x 10^4^ cells per well, covering ~65% of the well bottom).
5. Centrifuge INA-6 (5 min, 250 *g*) and resuspend them in a volume of diluted CM to reach a concentration of 6.8 x 10^5^ INA-6/mL.
6. Add 100 µL INA-6 suspension to hMSCs (end volume: 200 µL; end concentration: 33% (v/v) hMSC-conditioned medium).
7. Incubate for 24 h at 37 °C and 5% CO_2_.

Prepare CM-INA6 reference:

1. Add 100 µL of fresh MSC-medium into each well of an empty 96-well plate (not coated).
2. Add 100 µL of INA-6 suspension (6.8 x 10^5^ INA-6/mL in diluted CM).
3. Incubate for 24 h at 37 °C and 5% CO_2_.

Collect CM-INA6 and nMA-INA6

1. Pre-warm well plate centrifuge to 37 °C.
2. Prepare a counter-weight by filling 200 µL of water into all wells of an empty 96-well plate.
3. Prepare well-plate sandwiches:
   1. Turn an empty 96-well plate (“catching plate”) upside down and place one on top of the co-culture-plate, the CM-INA6 reference plate, and the counter-weight so that all well openings align.
   2. Fix well plates using tape with reusable adhesive (e.g. Leukofix).
4. Turn both plates around. Medium will spill from the co-culture plate into the catching plate
5. Centrifuge plate for 40 seconds at 1000 rpm with the catching plate facing the ground.
6. Remove the adhesive tape and the co-culture plate.
7. Turn the co-culture plate around and add 30 µL of washing medium (MSC-medium 0% FCS, 3 mM EDTA) gently by touching the wall of each well and pressing the pipette slowly.
   1. *Work quickly to ensure that co-culture does not dry. We recommend using a multipette (Eppendorf).*
   2. *Many nMA-INA6 are removed by physical force applied by adding 30 µL of medium and not just by centrifugation. Hence, it is critical to apply the same dispensing technique across all replicates. We recommend using a multipette (Eppendorf) that can apply 30 µL with controllable pressure, since its push-button retains a long pushing path even for dispensing small volumes, unlike push-buttons from the usual 100 µL pipettes that reduce the pushing-path for smaller volumes.*
   3. *Centrifugation minimizes technical variability by replacing one step of manual pipetting. Also, it ensures that confluent MSCs remain unharmed. Manual pipetting on the other hand would require touching the well-bottom to remove all fluids which damages the adhesive hMSC layer.*
8. Turn the co-culture plate upside down, place it onto the catching plate and re-apply adhesive tape to fix the well plate sandwich.
9. Repeat steps 14-18 two more times until the catching plate contains 290 µL of medium in each well.
10. Pool CM-INA6 from the catching plate that was fixed to the reference plate.
11. Pool nMA-INA6 from the catching plate that was fixed to the co-culture plate.
12. Collect remaining INA-6 by adding 100 µL of PBS into each well of the catching plates, collect and pool with CM-INA6 or nMA-INA6.
13. Strain CM-INA6 and nMA-INA6 using 40 µm cell strainer.
14. Isolate MA-INA6 by continue with either accutase dissociation or rough pipetting.

Collect MA-INA6 by accutase dissociation followed by MAC sorting

1. Block 2 mL tubes with sorting buffer (PBS, 2 mM EDTA, 1% BSA) for 1 h at 4 °C.
2. Dilute accutase (Sigma Aldrich GmbH) (400-600 units/mL) 4-fold in cold PBS. Always keep accutase on ice, since accutase loses activity at room temperature.
3. Add 50 µL of cold accutase (directly after the last centrifugation step) and incubate co-culture plate for 5 minutes at 37 °C.
4. Place a co-culture plate onto a shaker and shake for 1 minute at 300 rpm.
5. Collect cell suspension from wells and stop the reaction by adding 500 µL of FCS to pooled cell suspension.
6. Evaluate presence of adherent INA-6 cells and the integrity of confluent hMSCs under the microscope.
7. Repeat steps 24-27 until all INA-6 cells have dissociated or until confluent hMSCs start to tear.
8. Strain cell suspension (30 µm). This yields MA-MSC.
9. Pellet MA-INA6, nMA-INA6 and CM-INA6 (1200 rpm, 10 min).
10. Resuspend MA-INA6 in 86 µL sorting buffer (PBS, 2 mM EDTA, 1% BSA)
11. Resuspend CM-INA6 and nMA-INA6 in 300 µL cold diluted accutase and incubate for 3 min at 37 °C to ensure equal treatment for all samples.
12. Stop accutase by adding 200 µL of FCS (100%).
13. Pellet CM-INA6 and nMA-INA6 (1200 rpm, 10 min) and resuspend in 86 µL sorting buffer (PBS, 2 mM EDTA, 1% BSA).
14. Transfer samples into 2 mL tubes that were blocked with sorting buffer
15. Add 10 µL of CD45 coated magnetic beads (Miltenyi Biotec B.V. & Co. KG, Bergisch Gladbach)
16. Place tubes into rotator and incubate for 15 minutes at 4 °C
17. Continue with MAC sorting according to the manual. Use an MS column and wash 3 times.
18. Improve purity of eluted MA-INA6 by straining eluate (30 µm) (wash strainer using 1 mL of sorting buffer) and applying it onto an MS column a second time. Wash three times.
19. Collect 20 µL per eluate and apply it onto a 96-well plate to evaluate purity.
    1. Incubate plate for 24 h.
    2. Count the number of adherent cells (hMSCs) per INA-6 using phase contrast microscopy.
    3. *We reached a mean purity of 3.2 x 10^-4^ (±2.2 x 10^-4^) hMSCs per MA-INA6.*
    4. *hMSC contamination did not have an impact on RNAseq, since those genes that are highly expressed in hMSCs (VCAM1, ALPL, FGF5, FGFR2), did not appear as differentially expressed in MA-INA6 (data not shown). RNAseq detected 0.44 ±0.16 CPM-normalized counts of VCAM1 transcripts in MA-INA6, however, it was excluded like all genes with less than 1 count in at least 2 of 5 replicates.*
20. Count cells using a Neubauer chamber.
21. Pellet samples (250 *g* for 5 min).
22. Resuspend in respective medium or lysis buffer (e.g. RA1 for RNA extraction).

Collect MA-INA6 by rough pipetting (no MAC sorting)

1. After the last centrifugation step, add hMSC-medium to each well of the co-culture plate to reach a volume of 150 µL.
   1. *Since the yield of MA-INA6 was large, we dissociated MA-INA-6 cells from hMSCs by vigorous pipetting (for further samples after RNAseq, see Supplementary Table 1). Since no enzymatic digestion is used, we reckoned that there would be no need for MAC sorting. Confluent hMSCs withstand this procedure and don’t dissociate as single cells, which can be removed by straining cells (30 µm). We reached similar purities as for MAC-sorting (data not shown).*
2. Using a multi-channel pipette (100 µL), gently raise 90 µL into the tips.
3. Lean pipette tip on the upper well-border and roughly pipette up and down once.
4. Repeat step 48 at the lower right and lower left well border (Total of 3 pipetting steps “Mercedes Star”).
5. Attach a catching plate onto the co-culture and centrifuge for 40 seconds at 500 rpm (28 *g*).
6. Repeat steps 46-50 until a sufficient amount of MA-INA6 is removed.
7. Control purity of MA-INA-6 by placing out aliquot onto an empty 96-well plate.
8. Collect MA-INA6 from catching plate.
9. Remove hMSCs by straining cell suspension (30 µm).
10. Count cells using a Neubauer chamber.
11. Pellet MA-INA6 (250 *g* for 5 min).
12. Resuspend in respective medium or lysis buffer.

Centrifugal force: We used a Hettich 1460 rotor (r = 124 mm) (Hettich GmbH & Co. KG, Tuttlingen, Germany). For calculating the centrifugal force that acts onto the co-culture within well plate sandwiches, we subtracted the height of the catching plate (14.4 mm, Greiner 96-well plate) and the depth of each well (10.9 mm). This yields a radius of 98.7 mm, which translates to the following centrifugal forces: 500 rpm: 28 *g*; 1000 rpm: 110 *g*; 2000 rpm: 441 *g*.

Washing medium containing EDTA: EDTA removes calcium from integrins which are required for adhesion. It is not strong enough to dissociate INA-6 from hMSCs, but could help with removing INA-6 from other INA-6. For generating samples for RNAseq, we added 3 mM of EDTA to washing medium. For further samples, we did not add EDTA to the washing medium, since we found that it does not increase yield for all biological replicates consistently (data not shown). We suspect that integrin-mediated adhesion depends on hMSC donor or internal variance of INA-6. We recommend using 3 mM of EDTA, however, this requires further optimizations like including an incubation time at 37 °C after the addition of washing medium to account for biological variance. However, this could take long incubation times of up to 60 minutes (Lai et al., 2022).

### Track Cell Number During WPSC

To track the cell count during WPSC, INA-6 were stained with CellTracker green and both in co-culturing- and catching plates were scanned after each centrifugation step. For each round of centrifugation, an empty catching plate was used. A pre-trained convolutional neural network (Intellesis, Zeiss) was fine-tuned to segment the scans into background, cells, and cell borders. Single cells were counted and the cumulative sum for each catching plate was calculated.

### Sub-Culturing After WPSC of MSC-Interacting INA-6 Subpopulations

After CM-INA6, nMA-INA6, and MA-INA6 were isolated, they were counted with a Neubauer chamber using all nine quadrants and diluted to 10^5^ cells/mL in MSC-medium (10% FCS, no IL-6 except for control). 100 µL of cell suspension was applied to 96-well plates, incubated for 48 h at 37 °C and 5% CO_2_ and then subjected to viability and apoptosis assays.

### RNA Isolation

Total RNA was isolated from INA-6 cells by using the NucleoSpin RNA II Purification Kit (Macherey-Nagel, Düren, Germany) according to the manufacturer’s instructions.

### RNAseq, Differential Expression and Functional Enrichment Analysis of INA-6 cells

FASTQ files were merged to the respective sample. The quality of FASTQ files was assessed with FastQC (Andrews, 2010) tool, and a joint report was created with MultiQC (Ewels et al., 2016) tool.

Fastq files were aligned with STAR (Dobin et al., 2013) to the GRCh38 reference genome build (Zerbino et al., 2018). Quality and alignment statistics of final BAM files were assessed with samtools stats (Li et al., 2009), and a joint report with FastQC reports by MultiQC was generated.

Raw read counts were generated with HTSeq (Anders et al., 2015) with the union method. HTSeq runs internally in STAR. Differential gene expression analysis was done with edgeR (Robinson et al., 2010) in R 3.6.3 (R Core Team, 2018), according to the edgeR manual.

Counts were merged and genes with zero counts in all samples were removed (number of genes: 36380).

The whole count table was annotated with R Bioconductor (Gentleman, n.d.) (Gentleman et al. 2004) human annotation data package org.Hs.eg.db (Carlson, 2016).

A DGEList Element was created with the raw counts, gene information, i.e. Ensembl GeneIDs, HUGO Symbol, Genename, and ENTREZ GeneIDs and a sample grouping meta data table.

y <- DGEList(counts=ct2[,-1:4], group=meta.data$group, genes=ct2[,1:4])

Counts were filtered to keep only those genes which have at least 1 read per million in at least 2 samples (number of genes: 14136). Afterwards normalization factors were recalculated.

keep <- rowSums(cpm(y)>1) >=2

y <- y[keep, , keep.lib.size = FALSE]

y1 <- calcNormFactors(y)

A design matrix was created with grouping factor by treatment condition (group=F1, F2, F3, which are abbreviations for CM-INA6, nMA-INA6, MA-INA6, respectively)

design = model.matrix(~0+group)

Dispersion was estimated, the resulting coefficient of biological variation (BCV) is 0.135, i.e. BCV expression values vary up and down by 13.5% between samples.

y1.1 <- estimateDisp(y1, design)

BCV <- sqrt(model.F$y1.1$common.dispersion)

A generalized linear (glmQLFit function) model was fitted.

fit <- glmQLFit(y1.1, design)

and pairwise comparisons were made, e.g.

F1vsF2 <- glmQLFTest(fit, contrast = makeContrasts(groupF1 - groupF2, levels = design))

top significant differential expressed genes were written to a table

DE.F1vsF2 <- topTags(F1vsF2, n=nrow(F1vsF2), p.value = 0.05)

Afterwards, gene list of differentially expressed genes were used for functional enrichment analysis with metascape (Zhou et al., 2019).

### RT-qPCR

For cDNA synthesis 1 µg of total RNA was reverse transcribed with Oligo(dT)15 primers and Random Primers (both Promega GmbH) and Superscript IV reverse transcriptase (Thermo Fisher Scientific) according to the manufacturer’s instructions. For quantitative PCR the cDNA was diluted 1:10 and qPCR was performed in 20 µl by using 2 µl of cDNA and 10 µl of GoTaq qPCR Master Mix (Promega GmbH) and 5 pmol of sequence-specific primers obtained from biomers.net GmbH (Ulm, Germany) or Qiagen GmbH (Hilden, Germany) (see Supplementary Table 3 for primer sequences and PCR conditions). qPCR conditions were as follows: 95°C for 3 min; 40 cycles: 95°C for 10 s; respective annealing temperature for 10 s; 72°C for 10 s; followed by melting curve analysis for the specificity of qPCR products by using an TOptical Gradient 96 PCR Thermal Cycler (Analytik Jena AG, Jena, Germany). Samples that showed unspecific byproducts were discarded. Ct values were measured in three technical replicates (triplicates). Non-detects were discarded. One of three technical replicates was treated as an outlier and excluded if its z-score crossed 1.5 *σ* technical variation. We normalized expression by the housekeeping gene *36B4*. Efficiencies were determined in each reaction by linear regression of log transformed amplification curve (Ramakers et al., 2003). Differential expression was calculated based on a modified ΔΔCt formula that separated exponents to apply individual efficiencies to each Ct value:

$$Fold Change = \frac{E_{tar}^{\Delta{Ct}_{tar}(co-treated)}}{E_{ref}^{\Delta{Ct}_{ref}(co-treated)}} = \frac{E_{tar, co}^{{Ct}_{tar, co}} : E_{tar, treated}^{{Ct}_{tar, treated}}}{E_{ref, co}^{{Ct}_{ref, co}} : E_{ref, treated}^{{Ct}_{ref, treated}}}$$

$E_{tar, co}$ = Efficiency of the target gene measured in the control sample
 ${Ct}_{tar, co}$ = Ct value of the target gene measured in the control sample

$tar$ = Target Gene; $ref$ = Reference Gene

$treated$ = Treated sample; $co$ = Control Sample

Fold change expression was normalized by the median of CM-INA6 (and not samplewise, as commonly used in ΔΔCt) since some genes were not expressed without direct hMSC contact (e.g. *MMP2*), and also in order to display variation of CM-INA6 next to nMA-INA6 and MA-INA6.

**Statistics**

For molecular analyses, each data point represents one biological replicate, which we define as the mean of all technical replicates of co-cultures that were seeded out from the same batch of hMSCs and/or INA-6 cells on the same day. For analyses of time-lapse recordings, each datapoint represents the normalized event count from a recording of one co-culture. We prioritized unique hMSCs for each biological replicate or recording (Supplementary Table 1). Bars and lines represent the mean and error bars represent the standard deviation of all hMSC donors or recordings (= all biological replicates).

Metric, normal distributed, dependent data was analyzed using factorial RM-ANOVA and paired Student’s t-test. Results of RM-ANOVA are reported as such: [F($df1$, $df2$) = $F$; p = *p-value*], with $df1$ being the degrees of freedom of the observed effect, $df2$ being the degrees of freedom of the error and $F$ being the F-statistic (Vallat, 2018). If sphericity was met, p-values were not corrected with the Greenhouse-Geisser method (p-unc).

$df1 = k-1$ $k$ = The number of groups (of a factor, if factorial RM-ANOVA)

$df2 = (k-1)(n-1)$ $n$ = The number of samples in each group

$F = \frac{{SS}_{Effect} \div df1}{{SS}_{Error} \div df2}$ $SS$ = Sums of squares for effect or error

If datapoints within dependent sample pairs were missing, such pairs were excluded from paired t-test while other pairs of the same subject remained.

Metric non-normal distributed, independent data was analyzed using Kruskal-Wallis H-test and Mann–Whitney U tests. Results of Kruskal-Wallis H-test was reported as such: [H($df$) = $H$], with $df$ being the degrees of freedom and $H$ being the Kruskal-Wallis H statistic, corrected for ties (Vallat, 2018).

$df = k-1$ $k$ = The number of groups

Metric bivariate non-normal distributed data was correlated using spearman’s rank correlation and reported as such: [$\rho$($df$) = $\rho$, p = *p-value*], with $\rho$being Spearman’s rank correlation coefficient. $df$ is calculated as such:

$df = n-2$ $n$ = The number of observations

These test were applied using the python (3.10) -packages pingouin (0.5.1). For three-factor RM-ANOVA we used statsmodels (0.14.0) (Seabold & Perktold, 2010; Vallat, 2018). Data was plotted using seaborn (Waskom, 2021) and plotastic (Kuric & Ebert, 2024). Sphericity was ensured by Mauchly’s test. Normality was checked with the Shapiro-Wilk test for n > 3.

Datapoints were log10 transformed to convert the scale from multiplicative (“foldchange”) to additive, or in order to fulfill sphericity requirements.

P-values derived from patient survival data were corrected using the Benjamini-Hochberg procedure. For other post-hoc analyses, p-values were not adjusted for family-wise error rate in order to minimize type I errors. To prevent type II errors, the same conclusions were validated by different experimental setups and through varying hMSCs donors across experiments (Supplementary Table 1).

Significant p-values from pairwise tests were annotated as stars between data groups (p-value: 0.05 > * > 0.01 > ** > 10^-3^ > *** 10^-4^ > ****). If too many significant pairs were detected, we annotated only those pairs of interest.

No power calculation was performed to determine sample size since samples were limited by availability of primary hMSC donors. Experiments were repeated until a minimum of three biological replicates were gathered.

### Patient Cohort, Analysis of Survival and Expression

Patient samples (n=873) were collected at the University Hospital Heidelberg and processed as described (Seckinger et al., 2017, 2018), and are available at the European Nucleotide Archive (ENA) via accession numbers PRJEB36223 and PRJEB37100. Consecutive patients with monoclonal gammopathy of unknown significance (MGUS) (n = 62), asymptomatic (n = 259), symptomatic, therapy-requiring (n = 764), and relapsed/refractory myeloma (n = 90), as well as healthy donors (n = 19) as comparators were included in the study approved by the ethics committee (#229/2003, #S-152/2010) after written informed consent.

Gene expression was measured by RNA sequencing as previously described (Seckinger et al., 2018). Gene expression is defined as the log2 transformed value of normalized counts + 1 (as pseudocount). Progression-free (PFS) and overall survival (OS) was analyzed for the subset of previously untreated symptomatic myeloma patients. For delineating “high” and “low” expression of target adhesion (n=101) and cell cycle (n=173) genes, thresholds per gene were calculated with maximally selected rank statistics by the maxstat package in R (Hothorn & Lausen, n.d.). PFS and OS were analyzed for high *vs*. low expression with the Kaplan-Meier method (Kaplan & Meier, 1958). Significant differences between the curves were analyzed with log-rank tests (Harrington & Fleming, 1982). P-values were corrected for multiple testing by the Benjamini-Hochberg method. Analyses were performed with R version 3.6.3 (R Core Team, 2018).

## References

Anders, S., Pyl, P. T., & Huber, W. (2015). HTSeq—A Python framework to work with high-throughput sequencing data. *Bioinformatics (Oxford, England)*, *31*(2), 166–169. https://doi.org/10.1093/bioinformatics/btu638

Andrews, S. (2010). *FASTQC. A quality control tool for high throughput sequence data*.

Burger, R., Guenther, A., Bakker, F., Schmalzing, M., Bernand, S., Baum, W., Duerr, B., Hocke, G. M., Steininger, H., Gebhart, E., & Gramatzki, M. (2001). Gp130 and ras mediated signaling in human plasma cell line INA-6: A cytokine-regulated tumor model for plasmacytoma. *The Hematology Journal: The Official Journal of the European Haematology Association*, *2*(1), 42–53. https://doi.org/10.1038/sj.thj.6200075

Carlson, M. (2016). Org.Hs.eg.db. *Bioconductor*. https://doi.org/10.18129/B9.bioc.org.Hs.eg.db

Chatterjee, M., Hönemann, D., Lentzsch, S., Bommert, K., Sers, C., Herrmann, P., Mathas, S., Dörken, B., & Bargou, R. C. (2002). In the presence of bone marrow stromal cells human multiple myeloma cells become independent of the IL-6/gp130/STAT3 pathway. *Blood*, *100*(9), 3311–3318. https://doi.org/10.1182/blood-2002-01-0102

Dobin, A., Davis, C. A., Schlesinger, F., Drenkow, J., Zaleski, C., Jha, S., Batut, P., Chaisson, M., & Gingeras, T. R. (2013). STAR: Ultrafast universal RNA-seq aligner. *Bioinformatics*, *29*(1), 15–21. https://doi.org/10.1093/bioinformatics/bts635

Ewels, P., Magnusson, M., Lundin, S., & Käller, M. (2016). MultiQC: Summarize analysis results for multiple tools and samples in a single report. *Bioinformatics*, *32*(19), 3047–3048. https://doi.org/10.1093/bioinformatics/btw354

Fernandez-Rebollo, E., Mentrup, B., Ebert, R., Franzen, J., Abagnale, G., Sieben, T., Ostrowska, A., Hoffmann, P., Roux, P.-F., Rath, B., Goodhardt, M., Lemaitre, J.-M., Bischof, O., Jakob, F., & Wagner, W. (2017). Human Platelet Lysate versus Fetal Calf Serum: These Supplements Do Not Select for Different Mesenchymal Stromal Cells. *Scientific Reports*, *7*, 5132. https://doi.org/10.1038/s41598-017-05207-1

Gentleman. (n.d.). *Bioconductor—BiocViews*. Retrieved June 9, 2023, from https://bioconductor.org/packages/3.17/BiocViews.html

Gramatzki, M., Burger, R., Trautman, U., Marschalek, R., Lorenz, H., Hansen-Hagge, T. E., Baum, W., Bartram, C. R., Gebhart, E., & Kalden, J. R. (1994). *Two new interleukin-6 dependent plasma cell lines carrying a chromosomal abnormality involving the IL-6 gene locus. 84 Suppl. 1*, 173a–173a.

Greenstein, S., Krett, N. L., Kurosawa, Y., Ma, C., Chauhan, D., Hideshima, T., Anderson, K. C., & Rosen, S. T. (2003). Characterization of the MM.1 human multiple myeloma (MM) cell lines: A model system to elucidate the characteristics, behavior, and signaling of steroid-sensitive and -resistant MM cells. *Experimental Hematology*, *31*(4), 271–282. https://doi.org/10.1016/s0301-472x(03)00023-7

Harrington, D. P., & Fleming, T. R. (1982). A Class of Rank Test Procedures for Censored Survival Data. *Biometrika*, *69*(3), 553–566. https://doi.org/10.2307/2335991

Harris, C. R., Millman, K. J., van der Walt, S. J., Gommers, R., Virtanen, P., Cournapeau, D., Wieser, E., Taylor, J., Berg, S., Smith, N. J., Kern, R., Picus, M., Hoyer, S., van Kerkwijk, M. H., Brett, M., Haldane, A., del Río, J. F., Wiebe, M., Peterson, P., … Oliphant, T. E. (2020). Array programming with NumPy. *Nature*, *585*(7825), Article 7825. https://doi.org/10.1038/s41586-020-2649-2

Hothorn, T., & Lausen, B. (n.d.). *Maximally Selected Rank Statistics in R* [Computer software]. http://cran.r-project.org/web/packages/maxstat/index.html.

Kaplan, E. L., & Meier, P. (1958). Nonparametric Estimation from Incomplete Observations. *Journal of the American Statistical Association*, *53*(282), 457–481. https://doi.org/10.1080/01621459.1958.10501452

Kuric M, Ebert R. plotastic: Bridging Plotting and Statistics in Python. Journal of Open Source Software. 2024;9(95):6304. https://doi.org/10.21105/joss.06304

Lai, T.-Y., Cao, J., Ou-Yang, P., Tsai, C.-Y., Lin, C.-W., Chen, C.-C., Tsai, M.-K., & Lee, C.-Y. (2022). Different methods of detaching adherent cells and their effects on the cell surface expression of Fas receptor and Fas ligand. *Scientific Reports*, *12*(1), Article 1. https://doi.org/10.1038/s41598-022-09605-y

Li, H., Handsaker, B., Wysoker, A., Fennell, T., Ruan, J., Homer, N., Marth, G., Abecasis, G., Durbin, R., & 1000 Genome Project Data Processing Subgroup. (2009). The Sequence Alignment/Map format and SAMtools. *Bioinformatics*, *25*(16), 2078–2079. https://doi.org/10.1093/bioinformatics/btp352

Newville, M., Stensitzki, T., Allen, D. B., & Ingargiola, A. (2014). *LMFIT: Non-Linear Least-Square Minimization and Curve-Fitting for Python* [Computer software]. Zenodo. https://doi.org/10.5281/zenodo.11813

Nilsson, K., Bennich, H., Johansson, S. G., & Pontén, J. (1970). Established immunoglobulin producing myeloma (IgE) and lymphoblastoid (IgG) cell lines from an IgE myeloma patient. *Clinical and Experimental Immunology*, *7*(4), 477–489.

R Core Team. (2018). *R: A language and environment for statistical computing* [Manual]. R Foundation for Statistical Computing. https://www.R-project.org/

Ramakers, C., Ruijter, J. M., Deprez, R. H. L., & Moorman, A. F. M. (2003). Assumption-free analysis of quantitative real-time polymerase chain reaction (PCR) data. *Neuroscience Letters*, *339*(1), 62–66. https://doi.org/10.1016/S0304-3940(02)01423-4

Robinson, M. D., McCarthy, D. J., & Smyth, G. K. (2010). EdgeR: a Bioconductor package for differential expression analysis of digital gene expression data. *Bioinformatics (Oxford, England)*, *26*(1), 139–140. https://doi.org/10.1093/bioinformatics/btp616

Seckinger, A., Delgado, J. A., Moser, S., Moreno, L., Neuber, B., Grab, A., Lipp, S., Merino, J., Prosper, F., Emde, M., Delon, C., Latzko, M., Gianotti, R., Lüoend, R., Murr, R., Hosse, R. J., Harnisch, L. J., Bacac, M., Fauti, T., … Vu, M. D. (2017). Target Expression, Generation, Preclinical Activity, and Pharmacokinetics of the BCMA-T Cell Bispecific Antibody EM801 for Multiple Myeloma Treatment. *Cancer Cell*, *31*(3), 396–410. https://doi.org/10.1016/j.ccell.2017.02.002

Seckinger, A., Hillengass, J., Emde, M., Beck, S., Kimmich, C., Dittrich, T., Hundemer, M., Jauch, A., Hegenbart, U., Raab, M.-S., Ho, A. D., Schönland, S., & Hose, D. (2018). CD38 as Immunotherapeutic Target in Light Chain Amyloidosis and Multiple Myeloma-Association With Molecular Entities, Risk, Survival, and Mechanisms of Upfront Resistance. *Frontiers in Immunology*, *9*, 1676. https://doi.org/10.3389/fimmu.2018.01676

Vallat, R. (2018). Pingouin: Statistics in Python. *Journal of Open Source Software*, *3*(31), 1026. https://doi.org/10.21105/joss.01026

Weetall, M., Hugo, R., Maida, S., West, S., Wattanasin, S., Bouhel, R., Weitz-Schmidt, G., Lake, P., & Friedman, C. (2001). A Homogeneous Fluorometric Assay for Measuring Cell Adhesion to Immobilized Ligand Using V-Well Microtiter Plates. *Analytical Biochemistry*, *293*(2), 277–287. https://doi.org/10.1006/abio.2001.5140

Zerbino, D. R., Achuthan, P., Akanni, W., Amode, M. R., Barrell, D., Bhai, J., Billis, K., Cummins, C., Gall, A., Girón, C. G., Gil, L., Gordon, L., Haggerty, L., Haskell, E., Hourlier, T., Izuogu, O. G., Janacek, S. H., Juettemann, T., To, J. K., … Flicek, P. (2018). Ensembl 2018. *Nucleic Acids Research*, *46*(D1), D754–D761. https://doi.org/10.1093/nar/gkx1098

Zhou, Y., Zhou, B., Pache, L., Chang, M., Khodabakhshi, A. H., Tanaseichuk, O., Benner, C., & Chanda, S. K. (2019). Metascape provides a biologist-oriented resource for the analysis of systems-level datasets. *Nature Communications*, *10*(1), Article 1. https://doi.org/10.1038/s41467-019-09234-6
