# Supplementary material for: Modeling Myeloma Dissemination In Vitro with hMSC-interacting Subpopulations of INA-6 Cells and Their Aggregation/Detachment Dynamics
Source: Cancer Res Commun. 2024 Apr 29;4(4):1150–64. doi: 10.1158/2767-9764.CRC-23-0411 (PMC11057410; doi:10.1158/2767-9764.CRC-23-0411)
Supplement: Supplementary Table 1 [file crc-23-0411-s02.docx]

**Supplementary Table 1:** List of hMSC donors, myeloma cell lines, and their mycoplasma test status. If no unique donors were available, hMSC donors were used twice for the same experiment at different passages. WPSC: Well plate sandwich centrifugation.

| **Cell Type** | **Donor / Line** | **Donor Ages** | **Donor Sex** | **Date of negative Mycoplasma test** | **Experiment(s)** | **Figures** |
| --- | --- | --- | --- | --- | --- | --- |
| **Myeloma**  **Cell Line** | **INA-6** | 80 | m | 09.02.22 | All | All |
|  | **U266** |  |  | 10.10.22 | - Validation of V-Well Adhesion Assay | S1E |
|  | **MM1.S** |  |  | 24.02.22 |  |  |
| **hMSC** | **1639** | 49 | m | not tested | - Validation of V-Well Adhesion Assay | S1E |
|  |  |  |  |  | - Time-lapse: INA-6 on dispersed hMSC | 1D; 2[A-E] |
|  | **1571** | 72 | m | not tested | - Saturation of hMSCs | 1[A-B] |
|  | **1573** | 47 | m | not tested |  |  |
|  | **1578** | 82 | m | not tested |  |  |
|  | **1842** | 63 | m | not tested | - INA-6 Viability dep. on time and hMSC adhesion surface (INA not washed off) | 1E right |
|  | **1843** | 60 | m | not tested |  |  |
|  | **1537** | 77 | f | not tested |  |  |
|  | **1794** | 82 | m | not tested | - INA-6 Viability dep. on time and hMSC adhesion surface (INA washed off) | 1[C, E left] |
|  | **1779** | 61 | m | not tested |  |  |
|  | **1849** | 69 | m | not tested |  |  |
|  | **1854** | 80 | f | not tested |  |  |
|  | **1605** | 71 | f | not tested | - Time-lapse: INA-6 on dispersed hMSC | 1D; 2[A-E] |
|  | **1650** | 57 | m | not tested |  |  |
|  | **1859** | 64 | f | not tested | - Time-lapse: INA-6 on confluent hMSC | 3[A-D] |
|  | **1863** | 79 | f | not tested |  |  |
|  | **1861** | 52 | f | not tested |  |  |
|  | **1818** | 81 | f | not tested | - Cell Cycle Profiling after V-well assay | 3G |
|  | **1824** | 82 | f | not tested | - V-well adhesion assay of mitotically blocked INA-6 followed by Cell Cycle Profiling after V-well assay (Donor measured twice, different passages) | 3[F,G] |
|  | **1827** | 56 | m | not tested | - V-well adhesion assay of mitotically blocked INA-6 followed by Cell Cycle Profiling after V-well assay |  |
|  | **1501** | 59 | m | not tested | - INA-6 AI-assisted count during WPSC (INA-6 stained with celltracker green) | 4B |
|  | **1643** | 75 | f | not tested |  |  |
|  | **1718** | 67 | m | not tested |  |  |
|  | **1720** | 58 | m | not tested |  |  |
|  | **1653** | 65 | m | not tested |  |  |
|  | **1591** | 78 | m | not tested | - WPSC (MACS) followed by RNAseq, Metascape analysis and qPCR validation | 4[A,C,D,E]; 5[A-C] |
|  |  |  |  |  | - WPSC (Wash) followed by qPCR-Validation and Luminescent Viability assays | 4[C-E], 4F |
|  | **1654** | 74 | m | not tested | - WPSC (MACS) followed by RNAseq, Metascape analysis and qPCR validation | 4[A,C,D,E]; 5[A-C] |
|  |  |  |  |  | - WPSC (Wash) followed by qPCR-Validation and Luminescent Viability assays | 4[C-E], 4F |
|  | **1655** | 78 | f | not tested | - WPSC (MACS) followed by RNAseq, Metascape analysis and qPCR validation | 4[A,C,D,E]; 5[A-C] |
|  | **1668** | 80 | f | not tested |  |  |
|  | **1670** | 66 | f | not tested |  |  |
|  | **1701** | 81 | m | not tested | - WPSC (Wash) followed by qPCR-Validation and Luminescent Viability assays | 4[C-E], 4F |
|  | **1702** | 79 | f | not tested |  |  |
|  | **1600** | 77 | m | not tested |  |  |
|  | **1681** | 56 | m | not tested | - WPSC (Wash) followed by Luminescent Viability assays | 4F |
|  | **1672** | 65 | m | not tested | - WPSC (Wash) followed by qPCR-Validation | 4[C-E] |
