# Supplementary material for: Modeling Myeloma Dissemination In Vitro with hMSC-interacting Subpopulations of INA-6 Cells and Their Aggregation/Detachment Dynamics
Source: Cancer Res Commun. 2024 Apr 29;4(4):1150–64. doi: 10.1158/2767-9764.CRC-23-0411 (PMC11057410; doi:10.1158/2767-9764.CRC-23-0411)
Supplement: Supplementary Table 2 [file crc-23-0411-s03.docx]

##### **Supplementary Table 2:** Adhesion genes (from Fig. 6A) categorized by a continuous downregulation across disease progression. Bone Marrow Plasma Cell (BMPC), Monoclonal Gammopathy of Undetermined Significance (MGUS), Smoldering Multiple Myeloma (sMM), Multiple Myeloma (MM), Multiple Myeloma Relapse (MMR). p-adj. = adjusted p-values (Benj.-Hoch.).

| **Regulation during disease progression** | **Gene** | **Ensemble ID** | **Progression Free / Overall Survival** | **Better Prognosis with high/low expression** | **Association of expression with survival** | |
| --- | --- | --- | --- | --- | --- | --- |
|  |  |  |  |  | **[p-unc]** | **[p-adj]** |
| **FALSE** | **ADAMTS1** | **ENSG00000154734** | **Prog. Free** | low | 0.031875 | 0.084719 |
|  |  |  | **Overall** | low | 0.048755 | 0.120104 |
|  | **ADAMTS2** | **ENSG00000087116** | **Prog. Free** | high | 0.63795 | 0.767059 |
|  |  |  | **Overall** | high | 0.811174 | 0.890528 |
|  | **BGN** | **ENSG00000182492** | **Prog. Free** | high | 0.38065 | 0.533967 |
|  |  |  | **Overall** | high | 0.279004 | 0.426961 |
|  | **CAVIN1** | **ENSG00000177469** | **Prog. Free** | high | 0.407479 | 0.548739 |
|  |  |  | **Overall** | high | 0.210903 | 0.3492 |
|  | **CCDC80** | **ENSG00000091986** | **Prog. Free** | high | 0.002038 | 0.015833 |
|  |  |  | **Overall** | high | 0.023743 | 0.077356 |
|  | **CCN1** | **ENSG00000142871** | **Prog. Free** | high | 0.285568 | 0.443729 |
|  |  |  | **Overall** | low | 0.931563 | 0.961309 |
|  | **CCN2** | **ENSG00000118523** | **Prog. Free** | high | 0.030562 | 0.083425 |
|  |  |  | **Overall** | high | 0.002889 | 0.024263 |
|  | **CCNE2** | **ENSG00000175305** | **Prog. Free** | low | 0.012138 | 0.046195 |
|  |  |  | **Overall** | low | 0.000534 | 0.008638 |
|  | **CDH11** | **ENSG00000140937** | **Prog. Free** | high | 0.413948 | 0.550115 |
|  |  |  | **Overall** | high | 0.044627 | 0.117163 |
|  | **CEMIP** | **ENSG00000103888** | **Prog. Free** | high | 0.798984 | 0.877146 |
|  |  |  | **Overall** | low | 0.287022 | 0.428378 |
|  | **COL12A1** | **ENSG00000111799** | **Prog. Free** | high | 0.340978 | 0.491983 |
|  |  |  | **Overall** | low | 0.829338 | 0.900679 |
|  | **COL16A1** | **ENSG00000084636** | **Prog. Free** | low | 0.281112 | 0.443629 |
|  |  |  | **Overall** | low | 0.162895 | 0.293792 |
|  | **COL4A1** | **ENSG00000187498** | **Prog. Free** | high | 0.040286 | 0.098969 |
|  |  |  | **Overall** | high | 0.009472 | 0.039863 |
|  | **COL4A2** | **ENSG00000134871** | **Prog. Free** | high | 0.0124 | 0.046195 |
|  |  |  | **Overall** | high | 0.175895 | 0.3063 |
|  | **COL5A1** | **ENSG00000130635** | **Prog. Free** | high | 0.368403 | 0.524066 |
|  |  |  | **Overall** | low | 0.860512 | 0.914414 |
|  | **COL6A3** | **ENSG00000163359** | **Prog. Free** | low | 0.103315 | 0.208697 |
|  |  |  | **Overall** | low | 0.197836 | 0.336625 |
|  | **COL8A1** | **ENSG00000144810** | **Prog. Free** | high | 0.680745 | 0.807636 |
|  |  |  | **Overall** | high | 0.289334 | 0.428378 |
|  | **CREB3L1** | **ENSG00000157613** | **Prog. Free** | low | 0.165978 | 0.310441 |
|  |  |  | **Overall** | low | 0.047989 | 0.120104 |
|  | **EDIL3** | **ENSG00000164176** | **Prog. Free** | high | 0.863476 | 0.899083 |
|  |  |  | **Overall** | low | 0.496663 | 0.611744 |
|  | **F3** | **ENSG00000117525** | **Prog. Free** | high | 0.091858 | 0.197397 |
|  |  |  | **Overall** | high | 0.009177 | 0.039863 |
|  | **FBN1** | **ENSG00000166147** | **Prog. Free** | high | 0.472247 | 0.603376 |
|  |  |  | **Overall** | low | 0.401546 | 0.533633 |
|  | **FLNC** | **ENSG00000128591** | **Prog. Free** | high | 0.18539 | 0.329735 |
|  |  |  | **Overall** | low | 0.474071 | 0.598515 |
|  | **FN1** | **ENSG00000115414** | **Prog. Free** | high | 0.843432 | 0.896701 |
|  |  |  | **Overall** | low | 0.421268 | 0.552573 |
|  | **FOSB** | **ENSG00000125740** | **Prog. Free** | low | 0.585138 | 0.712035 |
|  |  |  | **Overall** | high | 0.942273 | 0.961309 |
|  | **GJA1** | **ENSG00000152661** | **Prog. Free** | high | 0.333512 | 0.491983 |
|  |  |  | **Overall** | low | 0.34262 | 0.467631 |
|  | **GREM1** | **ENSG00000166923** | **Prog. Free** | high | 0.457976 | 0.59302 |
|  |  |  | **Overall** | low | 0.591104 | 0.685623 |
|  | **HBEGF** | **ENSG00000113070** | **Prog. Free** | low | 0.145103 | 0.281656 |
|  |  |  | **Overall** | low | 0.051592 | 0.124067 |
|  | **HTRA1** | **ENSG00000166033** | **Prog. Free** | high | 0.01203 | 0.046195 |
|  |  |  | **Overall** | high | 0.040407 | 0.116603 |
|  | **IGFBP3** | **ENSG00000146674** | **Prog. Free** | high | 0.248011 | 0.410641 |
|  |  |  | **Overall** | low | 0.841566 | 0.904236 |
|  | **IGFBP7** | **ENSG00000163453** | **Prog. Free** | low | 0.009533 | 0.043766 |
|  |  |  | **Overall** | low | 0.024942 | 0.078722 |
|  | **ITGA11** | **ENSG00000137809** | **Prog. Free** | high | 0.97438 | 0.97438 |
|  |  |  | **Overall** | low | 0.966513 | 0.976178 |
|  | **KLF11** | **ENSG00000172059** | **Prog. Free** | low | 0.229416 | 0.39273 |
|  |  |  | **Overall** | low | 0.060892 | 0.133697 |
|  | **LAMB1** | **ENSG00000091136** | **Prog. Free** | high | 0.477921 | 0.603376 |
|  |  |  | **Overall** | high | 0.604163 | 0.685623 |
|  | **LOX** | **ENSG00000113083** | **Prog. Free** | low | 0.748901 | 0.840433 |
|  |  |  | **Overall** | low | 0.035028 | 0.104055 |
|  | **MMP2** | **ENSG00000087245** | **Prog. Free** | high | 2.29E-05 | 0.002316 |
|  |  |  | **Overall** | high | 0.044615 | 0.117163 |
|  | **NFKBIZ** | **ENSG00000144802** | **Prog. Free** | high | 0.725256 | 0.832396 |
|  |  |  | **Overall** | high | 0.310216 | 0.441293 |
|  | **NR4A1** | **ENSG00000123358** | **Prog. Free** | high | 0.0214 | 0.065497 |
|  |  |  | **Overall** | high | 0.060042 | 0.133697 |
|  | **NR4A2** | **ENSG00000153234** | **Prog. Free** | high | 0.275313 | 0.441375 |
|  |  |  | **Overall** | high | 0.11176 | 0.217072 |
|  | **OSMR** | **ENSG00000145623** | **Prog. Free** | high | 0.000567 | 0.007153 |
|  |  |  | **Overall** | high | 0.01287 | 0.046422 |
|  | **PDGFRB** | **ENSG00000113721** | **Prog. Free** | high | 0.691005 | 0.807636 |
|  |  |  | **Overall** | high | 0.599357 | 0.685623 |
|  | **POSTN** | **ENSG00000133110** | **Prog. Free** | low | 0.858041 | 0.899083 |
|  |  |  | **Overall** | low | 0.496348 | 0.611744 |
|  | **PTX3** | **ENSG00000163661** | **Prog. Free** | high | 0.020943 | 0.065497 |
|  |  |  | **Overall** | high | 0.045241 | 0.117163 |
|  | **PXDN** | **ENSG00000130508** | **Prog. Free** | low | 0.403966 | 0.548739 |
|  |  |  | **Overall** | low | 0.172495 | 0.305648 |
|  | **SERPINE1** | **ENSG00000106366** | **Prog. Free** | low | 0.543711 | 0.669693 |
|  |  |  | **Overall** | high | 0.869146 | 0.914414 |
|  | **SERPINH1** | **ENSG00000149257** | **Prog. Free** | low | 0.001825 | 0.015833 |
|  |  |  | **Overall** | low | 0.004399 | 0.026138 |
|  | **SIX1** | **ENSG00000126778** | **Prog. Free** | high | 0.784446 | 0.870649 |
|  |  |  | **Overall** | high | 0.592089 | 0.685623 |
|  | **SMAD3** | **ENSG00000166949** | **Prog. Free** | low | 0.027411 | 0.0791 |
|  |  |  | **Overall** | low | 0.016437 | 0.055338 |
|  | **SPARC** | **ENSG00000113140** | **Prog. Free** | high | 0.073989 | 0.162455 |
|  |  |  | **Overall** | high | 0.244069 | 0.391285 |
|  | **SPOCK1** | **ENSG00000152377** | **Prog. Free** | low | 0.531524 | 0.662765 |
|  |  |  | **Overall** | low | 0.303273 | 0.437579 |
|  | **SULF1** | **ENSG00000137573** | **Prog. Free** | high | 0.190403 | 0.331564 |
|  |  |  | **Overall** | high | 0.388706 | 0.523458 |
|  | **THBS2** | **ENSG00000186340** | **Prog. Free** | low | 0.318676 | 0.480392 |
|  |  |  | **Overall** | high | 0.292654 | 0.428378 |
|  | **VPS37B** | **ENSG00000139722** | **Prog. Free** | low | 0.1478 | 0.281656 |
|  |  |  | **Overall** | low | 0.199975 | 0.336625 |
| **TRUE** | **ACTN1** | **ENSG00000072110** | **Prog. Free** | high | 0.170661 | 0.313396 |
|  |  |  | **Overall** | high | 0.007728 | 0.035478 |
|  | **ADAM12** | **ENSG00000148848** | **Prog. Free** | high | 0.019179 | 0.062487 |
|  |  |  | **Overall** | high | 0.081847 | 0.168704 |
|  | **AEBP1** | **ENSG00000106624** | **Prog. Free** | high | 0.010829 | 0.046195 |
|  |  |  | **Overall** | high | 0.057228 | 0.133697 |
|  | **AXL** | **ENSG00000167601** | **Prog. Free** | high | 0.001496 | 0.015105 |
|  |  |  | **Overall** | high | 3.64E-05 | 0.00184 |
|  | **CD99** | **ENSG00000002586** | **Prog. Free** | low | 0.916833 | 0.941333 |
|  |  |  | **Overall** | low | 0.083964 | 0.169607 |
|  | **COL1A1** | **ENSG00000108821** | **Prog. Free** | high | 0.000303 | 0.004367 |
|  |  |  | **Overall** | high | 0.000593 | 0.008638 |
|  | **COL1A2** | **ENSG00000164692** | **Prog. Free** | high | 0.298023 | 0.456066 |
|  |  |  | **Overall** | high | 0.566636 | 0.673297 |
|  | **COL3A1** | **ENSG00000168542** | **Prog. Free** | high | 0.025985 | 0.07719 |
|  |  |  | **Overall** | high | 0.010794 | 0.042917 |
|  | **COL5A2** | **ENSG00000204262** | **Prog. Free** | low | 0.74501 | 0.840433 |
|  |  |  | **Overall** | low | 0.99967 | 0.99967 |
|  | **COL6A1** | **ENSG00000142156** | **Prog. Free** | high | 0.011972 | 0.046195 |
|  |  |  | **Overall** | high | 0.011048 | 0.042917 |
|  | **COL6A2** | **ENSG00000142173** | **Prog. Free** | high | 0.261528 | 0.426037 |
|  |  |  | **Overall** | high | 0.3235 | 0.447582 |
|  | **CXCL12** | **ENSG00000107562** | **Prog. Free** | high | 0.000116 | 0.002927 |
|  |  |  | **Overall** | high | 0.000648 | 0.008638 |
|  | **CXCL8** | **ENSG00000169429** | **Prog. Free** | low | 0.839416 | 0.896701 |
|  |  |  | **Overall** | high | 0.224913 | 0.366391 |
|  | **CYP1B1** | **ENSG00000138061** | **Prog. Free** | high | 0.008641 | 0.041735 |
|  |  |  | **Overall** | high | 0.000684 | 0.008638 |
|  | **DCN** | **ENSG00000011465** | **Prog. Free** | high | 0.004827 | 0.030473 |
|  |  |  | **Overall** | high | 0.000247 | 0.008327 |
|  | **DUSP1** | **ENSG00000120129** | **Prog. Free** | high | 0.695686 | 0.807636 |
|  |  |  | **Overall** | high | 0.454061 | 0.583718 |
|  | **FBLN1** | **ENSG00000077942** | **Prog. Free** | high | 0.002676 | 0.019305 |
|  |  |  | **Overall** | high | 0.003734 | 0.026138 |
|  | **GNB3** | **ENSG00000111664** | **Prog. Free** | high | 0.003748 | 0.025234 |
|  |  |  | **Overall** | high | 0.005734 | 0.03048 |
|  | **GSTP1** | **ENSG00000084207** | **Prog. Free** | high | 0.972219 | 0.97438 |
|  |  |  | **Overall** | low | 0.668091 | 0.749746 |
|  | **IGFBP4** | **ENSG00000141753** | **Prog. Free** | high | 0.008677 | 0.041735 |
|  |  |  | **Overall** | high | 0.007089 | 0.034093 |
|  | **IL1R1** | **ENSG00000115594** | **Prog. Free** | high | 0.126318 | 0.250159 |
|  |  |  | **Overall** | high | 0.256501 | 0.398563 |
|  | **ITGA5** | **ENSG00000161638** | **Prog. Free** | high | 0.094893 | 0.19967 |
|  |  |  | **Overall** | high | 0.159113 | 0.29219 |
|  | **ITGAX** | **ENSG00000140678** | **Prog. Free** | high | 0.006717 | 0.036021 |
|  |  |  | **Overall** | high | 0.003123 | 0.024263 |
|  | **ITGB5** | **ENSG00000082781** | **Prog. Free** | low | 0.436018 | 0.57192 |
|  |  |  | **Overall** | high | 0.539497 | 0.648681 |
|  | **LAMA4** | **ENSG00000112769** | **Prog. Free** | high | 0.018518 | 0.062345 |
|  |  |  | **Overall** | high | 0.104178 | 0.206314 |
|  | **LAMB2** | **ENSG00000172037** | **Prog. Free** | high | 0.015472 | 0.053885 |
|  |  |  | **Overall** | high | 0.001354 | 0.013865 |
|  | **LOXL2** | **ENSG00000134013** | **Prog. Free** | high | 0.808671 | 0.878235 |
|  |  |  | **Overall** | low | 0.933264 | 0.961309 |
|  | **LRP1** | **ENSG00000123384** | **Prog. Free** | high | 0.006458 | 0.036021 |
|  |  |  | **Overall** | high | 0.000434 | 0.008638 |
|  | **LTBP2** | **ENSG00000119681** | **Prog. Free** | high | 9.03E-05 | 0.002927 |
|  |  |  | **Overall** | high | 0.011656 | 0.043603 |
|  | **LUM** | **ENSG00000139329** | **Prog. Free** | high | 0.05158 | 0.118399 |
|  |  |  | **Overall** | high | 0.065084 | 0.139862 |
|  | **MAP3K8** | **ENSG00000107968** | **Prog. Free** | high | 0.000958 | 0.010755 |
|  |  |  | **Overall** | high | 0.01617 | 0.055338 |
|  | **MAP4K4** | **ENSG00000071054** | **Prog. Free** | high | 0.041155 | 0.098969 |
|  |  |  | **Overall** | high | 0.31743 | 0.445284 |
|  | **MFAP5** | **ENSG00000197614** | **Prog. Free** | high | 0.000243 | 0.004094 |
|  |  |  | **Overall** | high | 0.004269 | 0.026138 |
|  | **MMP14** | **ENSG00000157227** | **Prog. Free** | high | 6.93E-05 | 0.002927 |
|  |  |  | **Overall** | high | 0.006691 | 0.033787 |
|  | **MXRA5** | **ENSG00000101825** | **Prog. Free** | high | 0.034865 | 0.088035 |
|  |  |  | **Overall** | high | 0.033819 | 0.103505 |
|  | **MYL9** | **ENSG00000101335** | **Prog. Free** | high | 0.000146 | 0.00295 |
|  |  |  | **Overall** | high | 1.56E-05 | 0.001572 |
|  | **NRP1** | **ENSG00000099250** | **Prog. Free** | high | 0.001888 | 0.015833 |
|  |  |  | **Overall** | high | 0.002212 | 0.020312 |
|  | **PAPLN** | **ENSG00000100767** | **Prog. Free** | high | 0.034256 | 0.088035 |
|  |  |  | **Overall** | high | 0.159113 | 0.29219 |
|  | **TEX14** | **ENSG00000121101** | **Prog. Free** | high | 0.237488 | 0.399771 |
|  |  |  | **Overall** | low | 0.518581 | 0.631044 |
|  | **TGFBI** | **ENSG00000120708** | **Prog. Free** | high | 0.102621 | 0.208697 |
|  |  |  | **Overall** | high | 0.004299 | 0.026138 |
|  | **TGM2** | **ENSG00000198959** | **Prog. Free** | high | 0.058634 | 0.131601 |
|  |  |  | **Overall** | high | 0.119621 | 0.227958 |
|  | **THBS1** | **ENSG00000137801** | **Prog. Free** | high | 0.39286 | 0.543545 |
|  |  |  | **Overall** | high | 0.456572 | 0.583718 |
|  | **TNC** | **ENSG00000041982** | **Prog. Free** | high | 0.012806 | 0.046195 |
|  |  |  | **Overall** | high | 0.004752 | 0.026663 |
|  | **TNS1** | **ENSG00000079308** | **Prog. Free** | high | 0.338737 | 0.491983 |
|  |  |  | **Overall** | high | 0.757617 | 0.840872 |
|  | **TPM1** | **ENSG00000140416** | **Prog. Free** | high | 0.029263 | 0.0821 |
|  |  |  | **Overall** | high | 0.001373 | 0.013865 |
|  | **TUBA1A** | **ENSG00000167552** | **Prog. Free** | low | 0.006776 | 0.036021 |
|  |  |  | **Overall** | low | 0.042929 | 0.117163 |
|  | **TUBB6** | **ENSG00000176014** | **Prog. Free** | low | 0.186088 | 0.329735 |
|  |  |  | **Overall** | low | 0.060071 | 0.133697 |
|  | **VCAN** | **ENSG00000038427** | **Prog. Free** | high | 0.042782 | 0.100487 |
|  |  |  | **Overall** | high | 0.080757 | 0.168704 |
|  | **ZFP36L1** | **ENSG00000185650** | **Prog. Free** | high | 0.922693 | 0.941333 |
|  |  |  | **Overall** | high | 0.24957 | 0.393852 |
