# Supplementary material for: Modeling Myeloma Dissemination In Vitro with hMSC-interacting Subpopulations of INA-6 Cells and Their Aggregation/Detachment Dynamics
Source: Cancer Res Commun. 2024 Apr 29;4(4):1150–64. doi: 10.1158/2767-9764.CRC-23-0411 (PMC11057410; doi:10.1158/2767-9764.CRC-23-0411)
Supplement: Supplementary Table 3 [file crc-23-0411-s04.docx]

**Supplementary Table 3:** List of primers. Some primers required a melting step to be performed before fluorescent readout to remove byproducts.

| **Primer** | **Sequence 5' - 3'** | **base pairs [bp]** | **annealing temp. [°C]** |
| --- | --- | --- | --- |
| *36B4_s* | tgcatcagtaccccattctatcat | 122 | 60 |
| *36B4_as* | aggcagatggatcagccaaga |  |  |
| *BCL6_s* | tagagcccataaaacggtcctcat | 221 | 55 + Melting Step at 77 °C |
| *BCL6_as* | cgcaaattgagccgagatgtgt |  |  |
| *BMP4_s* | tacatgcgggatctttaccg | 132 | 58 |
| *BMP4_as* | atgttcttcgtggtggaagc |  |  |
| *BTG2_s* | gtattcttgtagggccgacactaa | 264 | 60 + Melting Step at 78 °C |
| *BTG2_as* | tcttaaggtgattcggtttgggaa |  |  |
| *CXCL8_s* | actgagagtgattgagagtggacc | 251 | 55 + Melting Step at 77 °C |
| *CXCL8_as* | ccctacaacagacccacacaatac |  |  |
| *CXCL12_s* | gattcttcgaaagccatgttgcca | 119 | 56 |
| *CXCL12_as* | caatgcacacttgtctgttgttgt |  |  |
| *DCN_s* | caacaacaagcttaccagagtacct | 160 | 57 |
| *DCN_as* | tgaaaagactcacacccgaataaga |  |  |
| *DKK1_s* | gcactgatgagtactgcgctag | 129 | 56 |
| *DKK1_as* | ttttgcagtaattcccggggc |  |  |
| *IL10RB_s* | gagtgagcctgtctgtgagcaa | 139 | 55 |
| *IL10RB_as* | cttgtaaacgcaccacagcaag |  |  |
| *IL24_s* | caaacagttggacgtagaagcagc | 149 | 55 |
| *IL24_as* | tgaaatgacacagggaacaaacca |  |  |
| *LOX_s* | ctgctcagatttccccaaag | 125 | 57 |
| *LOX_as* | tggcatcaagcaggtcatag |  |  |
| *MMP2_s* | ttgtatttgatggcatcgctcaga | 155 | 56 |
| *MMP2_as* | cgtataccgcatcaatcttttccg |  |  |
| *MMP14_s* | cgacaagattgatgctgctc | 140 | 57 |
| *MMP14_as* | tcccttcccagactttgatg |  |  |
| *MUC1_s* | gcagcctctcgatataacctg | 200 | 58 |
| *MUC1_as* | gtaggtggggtactcgctca |  |  |
| *NOTCH2_s* | gtgcttgttgaacacttgtgcc | 185 | 55 |
| *NOTCH2_as* | cactcgcatctgtatccaccaatg |  |  |
| *OPG (TNFRSF11B)* | no sequence available  (Proprietary primers from Qiagen: QT00014294 TNFRSF11B_1_SG) |  | 60 |
| *PRICKLE1_s* | cagaggtatatcatgaaggacggc | 102 | 56 |
| *PRICKLE1_as* | gtccacaccaatatgttccccac |  |  |
| *TGM2_s* | caaccttctcatcgagtacttccg | 100 | 58 |
| *TGM2_as* | tcatccacgactccacccag |  |  |
| *TNFRSF1A_s* | ctccttcaccgcttcagaaaacc | 153 | 55 |
| *TNFRSF1A_as* | ttcactccaataatgccggtactg |  |  |
| *TRAF5_s* | tgccctgtagataaagaggtcatca | 177 | 56 |
| *TRAF5_as* | aacactgcacaggttgaaataagc |  |  |
