# Supplementary material for: Modeling Myeloma Dissemination In Vitro with hMSC-interacting Subpopulations of INA-6 Cells and Their Aggregation/Detachment Dynamics
Source: Cancer Res Commun. 2024 Apr 29;4(4):1150–64. doi: 10.1158/2767-9764.CRC-23-0411 (PMC11057410; doi:10.1158/2767-9764.CRC-23-0411)
Supplement: Supplementary Figures 1-8 — Supplementary Figure 1. Principle and quantification of the V-well adhesion assay of fluorescently labeled myeloma cells adapted by Weetall et al. 2001. Supplementary Figure 2. Validation of image cytometric analysis of cell cycle in four INA-6 cultures. Supplementary Figure 3. Cell cycle analysis of INA-6 pellets gained from V-Well Adhesion assay (Fig. 3). Supplementary Figure 4. Representative (one of the four independent sample sets as seen in Supplementary Figure 3) curve fitting analysis of cell cycle profiles generated by Image Cytometry. Supplementary Figure 5. Correlation of RNAseq with qPCR Left: Validation of RNAseq results (Fig. 4) with qPCR showing the log2(foldchange expression) of 18 genes. Supplementary Figure 6. Functional enrichment analysis by Metascape using genes that are differentially expressed between MSC-interacting subpopulations. Supplementary Figure 7. Expression levels of adhesion genes that are downregulated and associated with survival (p < 0.01). Bone Marrow Plasma Cell (BMPC), Monoclonal Gammopathy of Undetermined Significance (MGUS), Smoldering Multiple Myeloma (sMM), Multiple Myeloma (MM), Multiple Myeloma Relapse (MMR). Supplementary Figure 8. Expression levels of adhesion genes that are not downregulated and associated with survival (p < 0.01). [file crc-23-0411-s09.pdf]

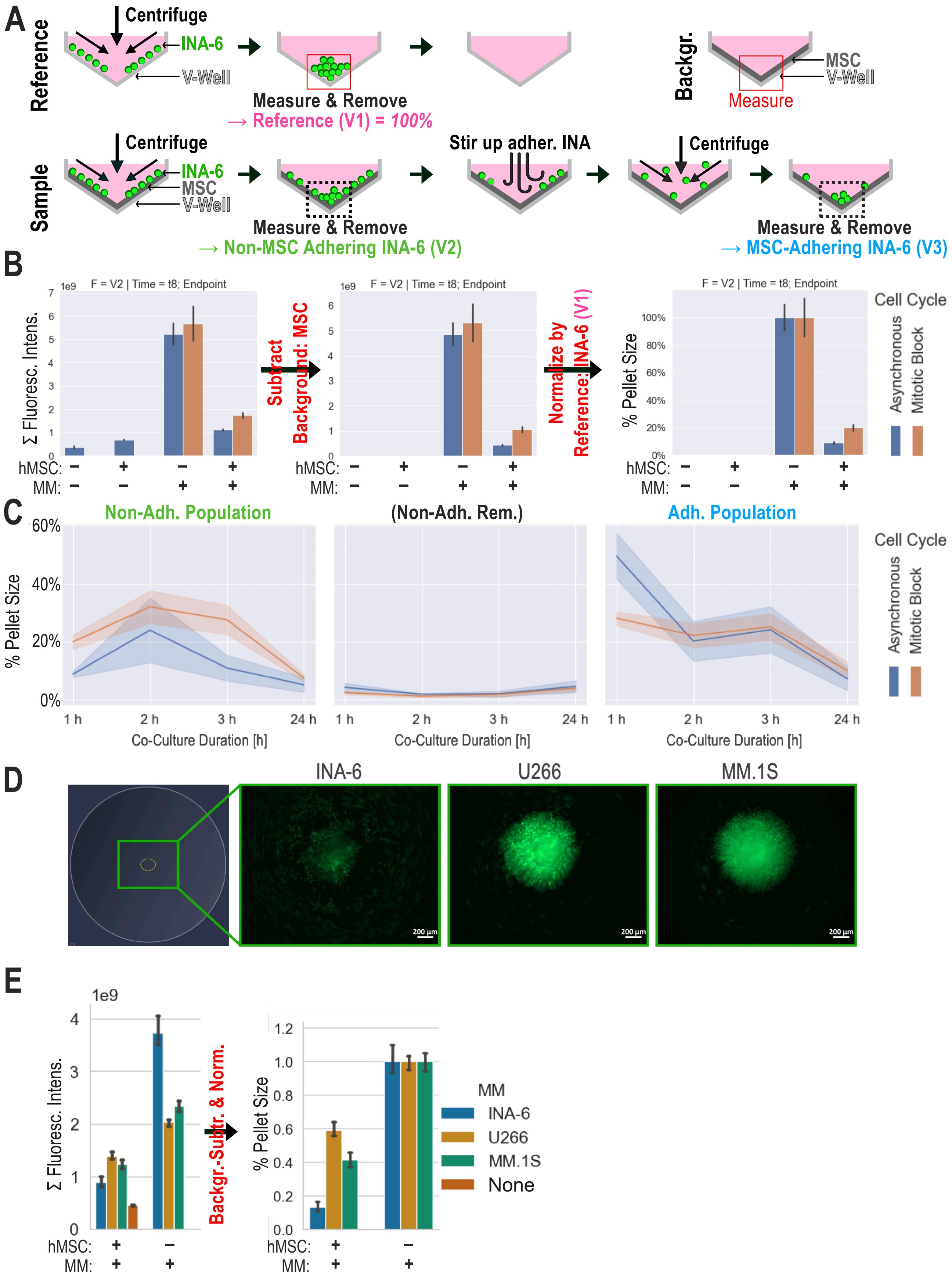
A
Reference
Centrifuge
INA-6
V-Well
Measure & Remove
→ Reference (V1) = 100%
Backgr.
MSC
V-Well
Measure
Sample
Centrifuge
INA-6
MSC
V-Well
Measure & Remove
→ Non-MSC Adhering INA-6 (V2)
Stir up adher. INA
Centrifuge
Measure & Remove
→ MSC-Adhering INA-6 (V3)
B
F = V2 | Time = t8; Endpoint
Σ Fluoresc. Intens.
Subtract Background: MSC
Normalize by Reference: INA-6 (V1)
% Pellet Size
Cell Cycle
Asynchronous
Mitotic Block
hMSC:
MM:
C
Non-Adh. Population
(Non-Adh. Rem.)
Adh. Population
% Pellet Size
Co-Culture Duration [h]
Cell Cycle
Asynchronous
Mitotic Block
D
INA-6
U266
MM.1S
E
Σ Fluoresc. Intens.
Backgr.-Subtr. & Norm.
% Pellet Size
MM
INA-6
U266
MM.1S
None
hMSC:
MM:

Supplementary Figure 1: Principle and quantification of the V-well adhesion assay of fluorescently labeled myeloma cells adapted by Weetall et al. 2001. **A:** Sample: Subsequent rounds of centrifugation and removal of cell pellet yielded the size of adhesive subpopulations. Fluorescently stained INA-6 cells were added to an hMSC monolayer. Non-adherent INA-6 cells (V2) were pelleted in the well-tip. Pellets were quantified by fluorescence brightness and isolated by pipetting. Immobile INA-6 cells (V3) were manually detached by forceful pipetting. Reference: Omitting adhesive hMSC-layer yielded ~100% non-adherent cells (V1) after the first centrifugation step; Background: hMSC monolayer was used as background signal. **B:** Calculation of the population size relative to total cells starting with pellet intensity. The shown example is the pellet gained by centrifuging mobile subpopulation (V2) after 1 h of co-culture. (see Fig. 3 for context): Intensity values from pellet images were summarized. After subtracting the unlabeled hMSC signal and normalization by a full-size pellet (reference), the resulting values represented the fraction of the adhesive subpopulation. **C:** One of three biological replicates summarized in Fig. 3. Line range shows the standard deviation of four technical replicates. Non.Adh. Rem.: Fluorescence signal after removal of V2. **D:** Example images of myeloma cell lines (INA-6, U266, MM.1S) pelleted in the tip of V-wells. The leftmost image shows the recorded area in a complete V-well. Scale bar = 200 µm. **E:** Results from (D) comparing adhesion strength of three myeloma cell lines to hMSC. Error bars represent technical deviation. MM=Multiple Myeloma.

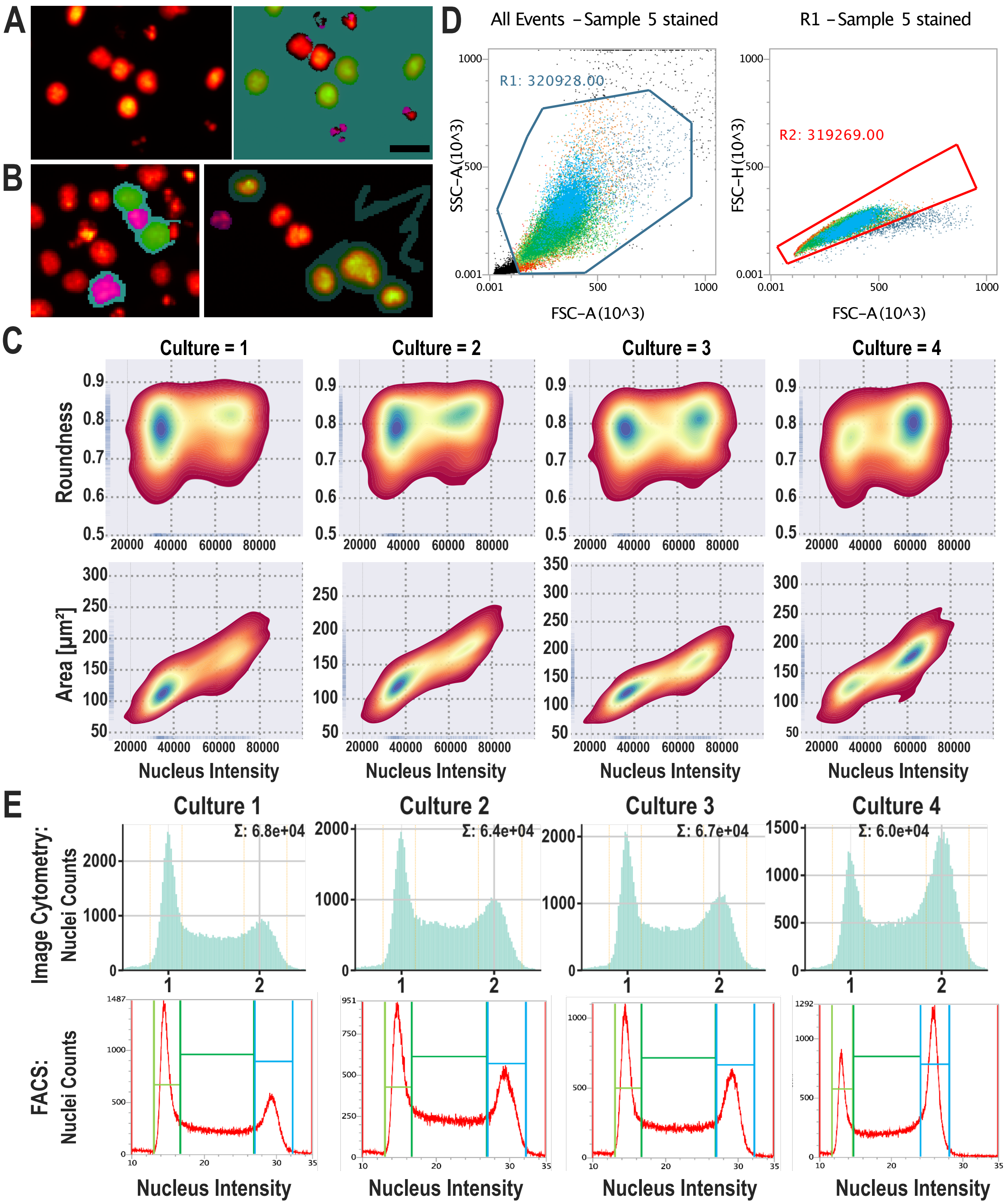

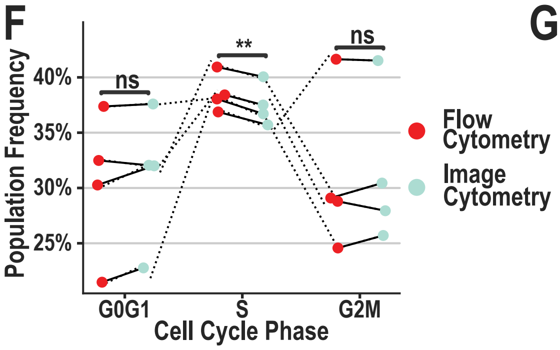

G

		G0G1		S		G2M		Total	
Cult.		Flow C.	Image C.	Flow C.	Image C.	Flow C.	Image C.	Flow C.	Image C.
4	Abs.	49067	13153	84234	20620	95120	23978	228421	57751
	Rel.	21.5%	22.8%	36.9%	35.7%	41.6%	41.5%	100%	100%
	Diff.		1.29%		1.17%		0.12%	mean:	0.86%
3	Abs.	78702	20833	93066	24376	70480	19787	2E+05	64996
	Rel.	32.5%	32.1%	38.4%	37.5%	29.1%	30.4%	100%	100%
	Diff.		0.44%		0.91%		1.35%	mean:	0.90%
2	Abs.	73746	19839	99737	24829	70147	17321	2E+05	61989
	Rel.	30.3%	32.0%	40.9%	40.1%	28.8%	27.9%	100%	100%
	Diff.		1.73%		0.88%		0.85%	mean:	1.16%
1	Abs.	98171	24709	99938	24123	64552	16895	3E+05	65727
	Rel.	37.4%	37.6%	38.0%	36.7%	24.6%	25.7%	100%	100%
	Diff.		0.22%		1.35%		1.13%	mean:	0.90%

Supplementary Figure 2: Validation of image cytometric analysis of cell cycle in four INA-6 cultures **A:** Left: Example image cytometric scan: INA-6 cells were stained with Hoechst33342 and scanned by automated fluorescence microscopy. Right: The image was segmented using a convolutional neural network (ZEISS ZEN intellesis) trained to discern healthy nuclei (green) from fragmented ones (magenta). Doublets are excluded by setting an area- and roundness threshold. Scale bar: 20 µm. **B:** Two example images from the training set. **C:** Quality of image cytometric data was ensured by plotting the distribution of nuclei brightnesses vs. the distribution of both nuclei-roundnesses and nuclei-areas. Nuclei with double fluorescence intensity have the same roundness while their area increases, as expected from a cell in G2 phase. **D:** The same samples from (C) were also measured with flow cytometry. Representative example of gating strategy: Left: Dead cells were excluded by setting a minimum threshold for side-scattering (SSC-A). Right: Doublets were excluded by setting a maximum threshold for forward scatter area (FSC-A) (sample “5” represents culture “4” in this figure). **E:** Cell cycle profiles of four independent INA-6 cultures were measured by both image cytometry (top) and flow cytometry (bottom). For both methods, frequencies of G0/G1, S, and G2M were summed up by setting fluorescence intensity thresholds. **F:** Image cytometry yields the same frequencies for G0/G1, S, and G2M when compared to flow cytometry. RM-ANOVA showed that the method has no significant effect on the frequencies of cell cycle populations [F(1,3)=1.421, p-unc=.32]. **G:** Results from (F) in tabular form. On average, frequencies for G0/G1, S, and G2M measured by Image cytometry differ by 0.95 percent points compared to flow cytometry measurement. Cult.: Culture; C.: Image cytometry; Abs.: Absolute cell count; Rel.: Relative cell count; Diff.: Difference between relative cell counts determined by flow cytometry and image cytometry.

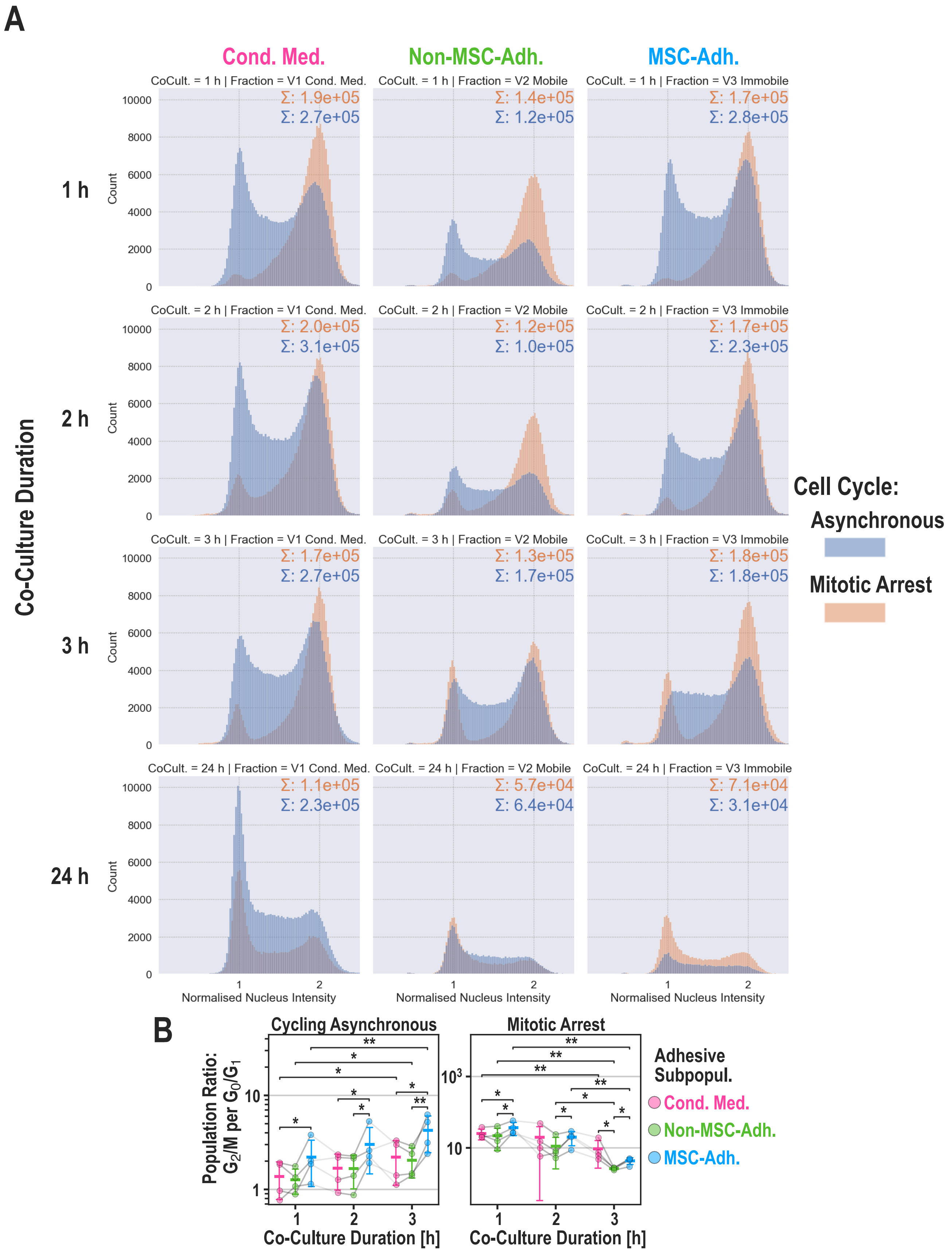
A
Cond. Med.
Non-MSC-Adh.
MSC-Adh.
Co-Culture Duration
1 h
2 h
3 h
24 h
Count
CoCult. = 1 h | Fraction = V1 Cond. Med.
Σ: 1.9e+05
Σ: 2.7e+05
CoCult. = 1 h | Fraction = V2 Mobile
Σ: 1.4e+05
Σ: 1.2e+05
CoCult. = 1 h | Fraction = V3 Immobile
Σ: 1.7e+05
Σ: 2.8e+05
CoCult. = 2 h | Fraction = V1 Cond. Med.
Σ: 2.0e+05
Σ: 3.1e+05
CoCult. = 2 h | Fraction = V2 Mobile
Σ: 1.2e+05
Σ: 1.0e+05
CoCult. = 2 h | Fraction = V3 Immobile
Σ: 1.7e+05
Σ: 2.3e+05
CoCult. = 3 h | Fraction = V1 Cond. Med.
Σ: 1.7e+05
Σ: 2.7e+05
CoCult. = 3 h | Fraction = V2 Mobile
Σ: 1.3e+05
Σ: 1.7e+05
CoCult. = 3 h | Fraction = V3 Immobile
Σ: 1.8e+05
Σ: 1.8e+05
CoCult. = 24 h | Fraction = V1 Cond. Med.
Σ: 1.1e+05
Σ: 2.3e+05
CoCult. = 24 h | Fraction = V2 Mobile
Σ: 5.7e+04
Σ: 6.4e+04
CoCult. = 24 h | Fraction = V3 Immobile
Σ: 7.1e+04
Σ: 3.1e+04
Normalised Nucleus Intensity
Cell Cycle:
Asynchronous
Mitotic Arrest
B
Cycling Asynchronous
Mitotic Arrest
Population Ratio: G2/M per G0/G1
Co-Culture Duration [h]
Adhesive Subpopul.
Cond. Med.
Non-MSC-Adh.
MSC-Adh.

Supplementary Figure 3: Cell cycle analysis of INA-6 pellets gained from V-Well Adhesion assay (Fig. 3). **A:** Cell cycle profiles of MSC-adhering subpopulations. INA-6 cells were synchronized by double thymidine block followed by nocodazole. Cell cycle was released directly before addition to hMSCs. Histograms were normalized and summed up across all biological replicates (n=4). Technical replicates (3) were pooled prior to cell cycle profiling. CoCult. = Co-culture duration. Fraction = Adhesion subpopulations. **B:** Similar figure to Fig. 3G displaying ratio of INA-6 populations (G2/M to G0/G1). **Statistics:** Paired t-test (B).

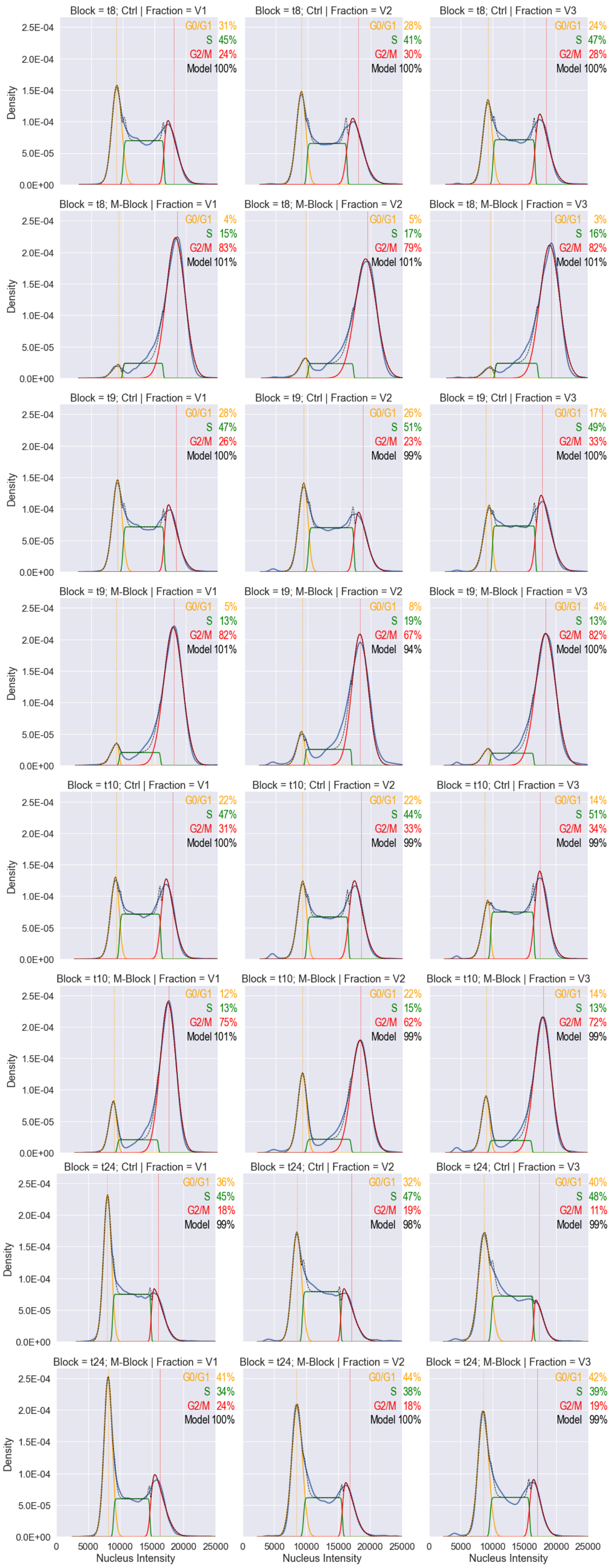

Block = t8; Ctrl | Fraction = V1
G0/G1 31%
S 45%
G2/M 24%
Model 100%
Block = t8; Ctrl | Fraction = V2
G0/G1 28%
S 41%
G2/M 30%
Model 100%
Block = t8; Ctrl | Fraction = V3
G0/G1 24%
S 47%
G2/M 28%
Model 100%
Block = t8; M-Block | Fraction = V1
G0/G1 4%
S 15%
G2/M 83%
Model 101%
Block = t8; M-Block | Fraction = V2
G0/G1 5%
S 17%
G2/M 79%
Model 101%
Block = t8; M-Block | Fraction = V3
G0/G1 3%
S 16%
G2/M 82%
Model 101%
Block = t9; Ctrl | Fraction = V1
G0/G1 28%
S 47%
G2/M 26%
Model 100%
Block = t9; Ctrl | Fraction = V2
G0/G1 26%
S 51%
G2/M 23%
Model 99%
Block = t9; Ctrl | Fraction = V3
G0/G1 17%
S 49%
G2/M 33%
Model 100%
Block = t9; M-Block | Fraction = V1
G0/G1 5%
S 13%
G2/M 82%
Model 101%
Block = t9; M-Block | Fraction = V2
G0/G1 8%
S 19%
G2/M 67%
Model 94%
Block = t9; M-Block | Fraction = V3
G0/G1 4%
S 13%
G2/M 82%
Model 100%
Block = t10; Ctrl | Fraction = V1
G0/G1 22%
S 47%
G2/M 31%
Model 100%
Block = t10; Ctrl | Fraction = V2
G0/G1 22%
S 44%
G2/M 33%
Model 99%
Block = t10; Ctrl | Fraction = V3
G0/G1 14%
S 51%
G2/M 34%
Model 99%
Block = t10; M-Block | Fraction = V1
G0/G1 12%
S 13%
G2/M 75%
Model 101%
Block = t10; M-Block | Fraction = V2
G0/G1 22%
S 15%
G2/M 62%
Model 99%
Block = t10; M-Block | Fraction = V3
G0/G1 14%
S 13%
G2/M 72%
Model 99%
Block = t24; Ctrl | Fraction = V1
G0/G1 36%
S 45%
G2/M 18%
Model 99%
Block = t24; Ctrl | Fraction = V2
G0/G1 32%
S 47%
G2/M 19%
Model 98%
Block = t24; Ctrl | Fraction = V3
G0/G1 40%
S 48%
G2/M 11%
Model 99%
Block = t24; M-Block | Fraction = V1
G0/G1 41%
S 34%
G2/M 24%
Model 100%
Block = t24; M-Block | Fraction = V2
G0/G1 44%
S 38%
G2/M 18%
Model 100%
Block = t24; M-Block | Fraction = V3
G0/G1 42%
S 39%
G2/M 19%
Model 99%
Density
2.5E-04
2.0E-04
1.5E-04
1.0E-04
5.0E-05
0.0E+00
0 5000 10000 15000 20000 25000
Nucleus Intensity

Supplementary Figure 4: Representative (one of the four independent sample sets as seen in Supplementary Figure 3) curve fitting analysis of cell cycle profiles generated by Image Cytometry. t8, t9, t10, and t24 refer to 1, 2, 3, and 24 hours after the addition of INA-6 cells to hMSCs.

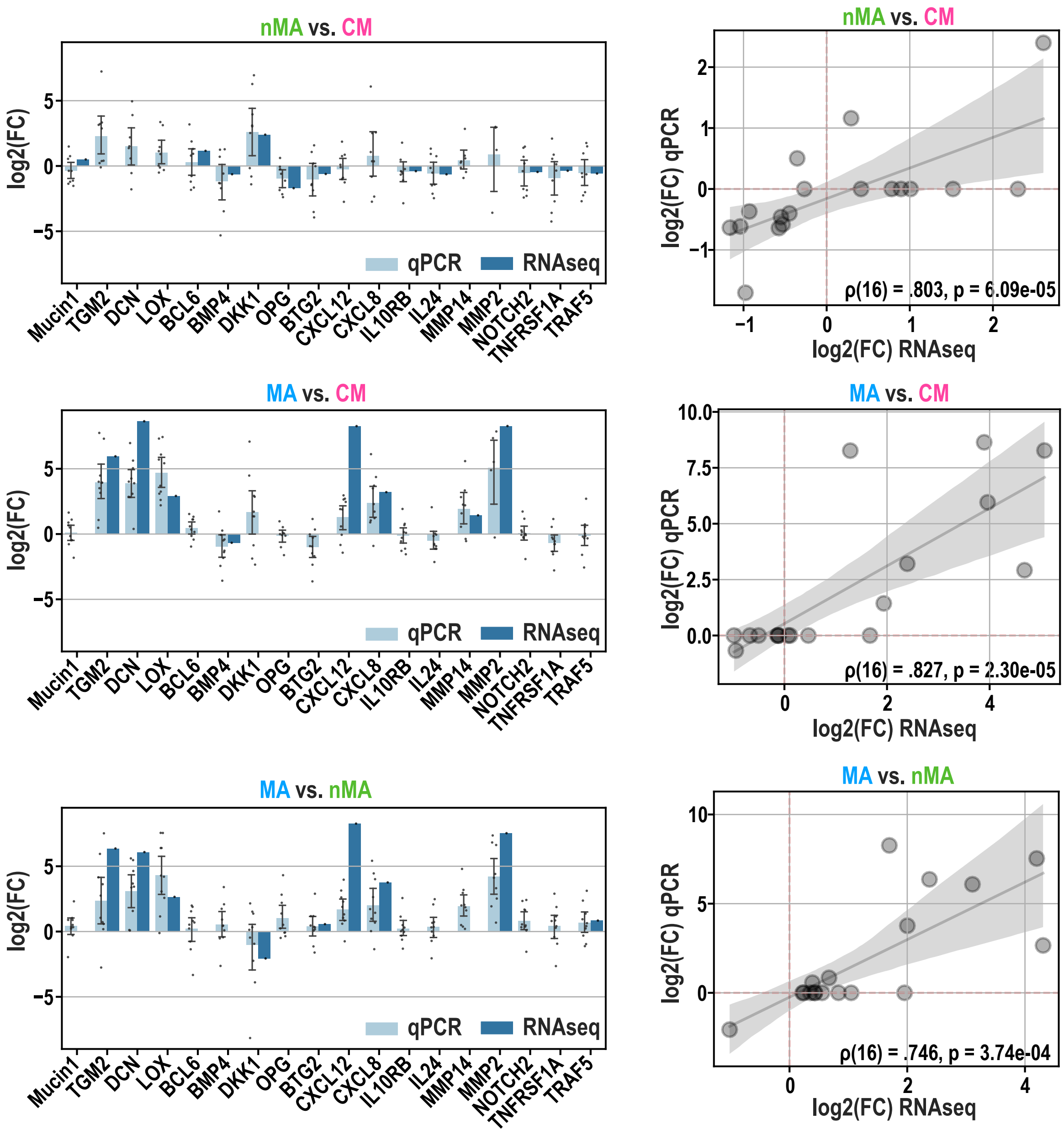

Supplementary Figure 5: Correlation of RNAseq with qPCR **Left:** Validation of RNAseq results (Fig. 4) with qPCR showing the log2(foldchange expression) of 18 genes. For qPCR, Datapoints each represent one biological replicate (n=10), which is the mean of technical replicates (n=3). Bar height represents mean of biological replicates, error bars show standard deviation of biological replicates. **Right:** Correlation between qPCR and RNAseq in terms of log2(mean foldchange expression per gene). Each dot represents one gene shown in the barplot to the left. Genes measured with qPCR that showed no differential expression in RNAseq were set to have a log2(FC) = 0. Shaded area shows the confidence interval of linear regression. Correlation coefficient (ρ) was calculated using Spearman’s rank. N = 18 genes. FC = fold change expression.

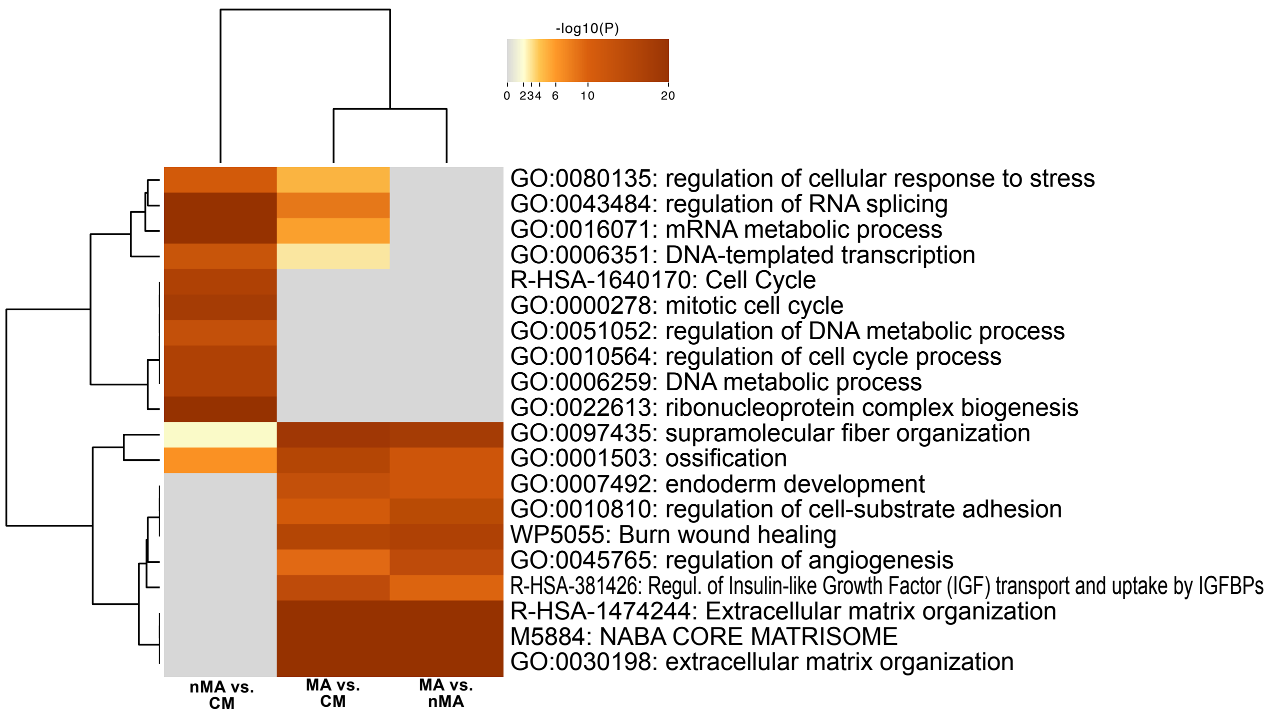

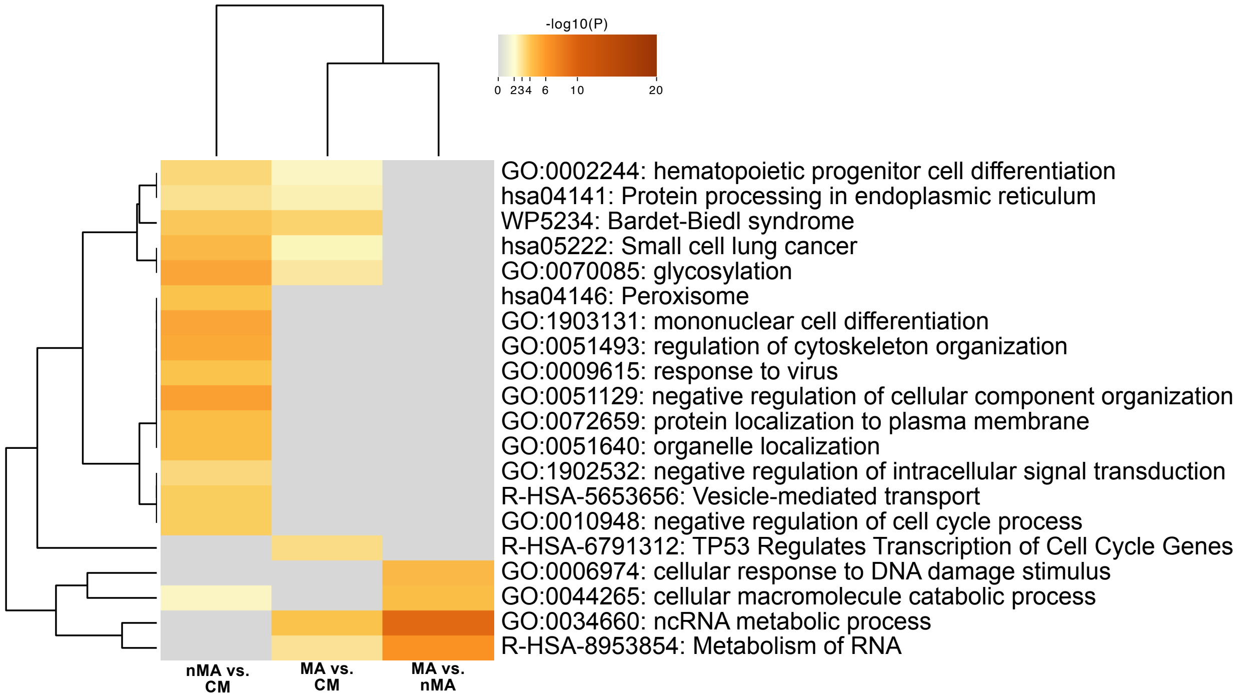

Supplementary Figure 6: Functional enrichment analysis by Metascape using genes that are differentially expressed between MSC-interacting subpopulations. **Top:** Upregulated genes. **Bottom**: Downregulated genes.

log2 RNA–Seq CYP1B1 (ENSG00000138061)

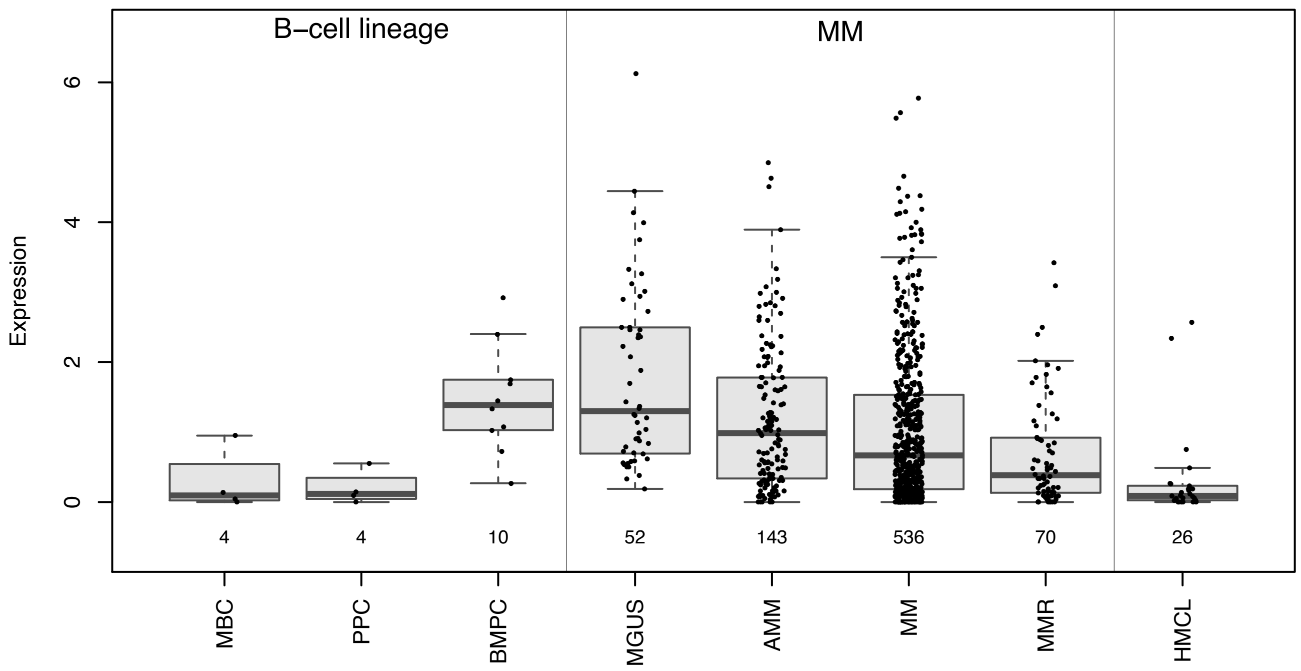

log2 RNA–Seq COL1A1 (ENSG00000108821)

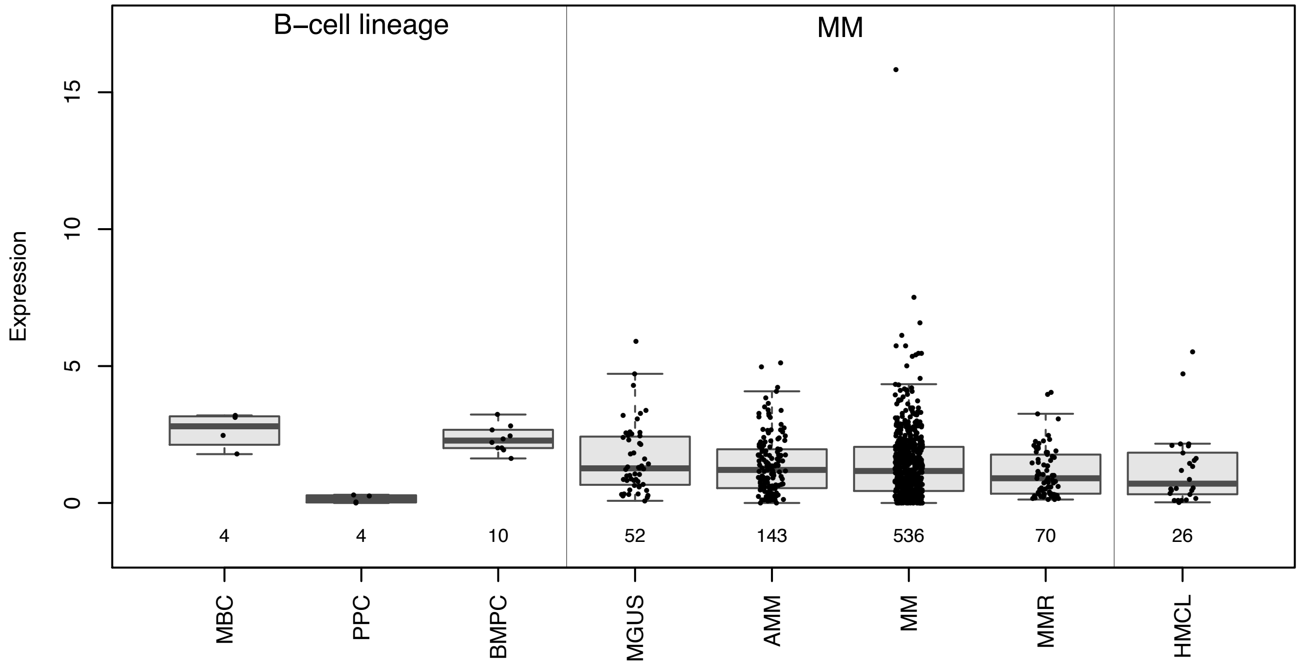

Supplementary Figure 7: Expression levels of adhesion genes that are downregulated and associated with survival ($p < 0.01$). Bone Marrow Plasma Cell (BMPC), Monoclonal Gammopathy of Undetermined Significance (MGUS), Smoldering Multiple Myeloma (sMM), Multiple Myeloma (MM), Multiple Myeloma Relapse (MMR).

log2 RNA–Seq LRP1 (ENSG00000123384)

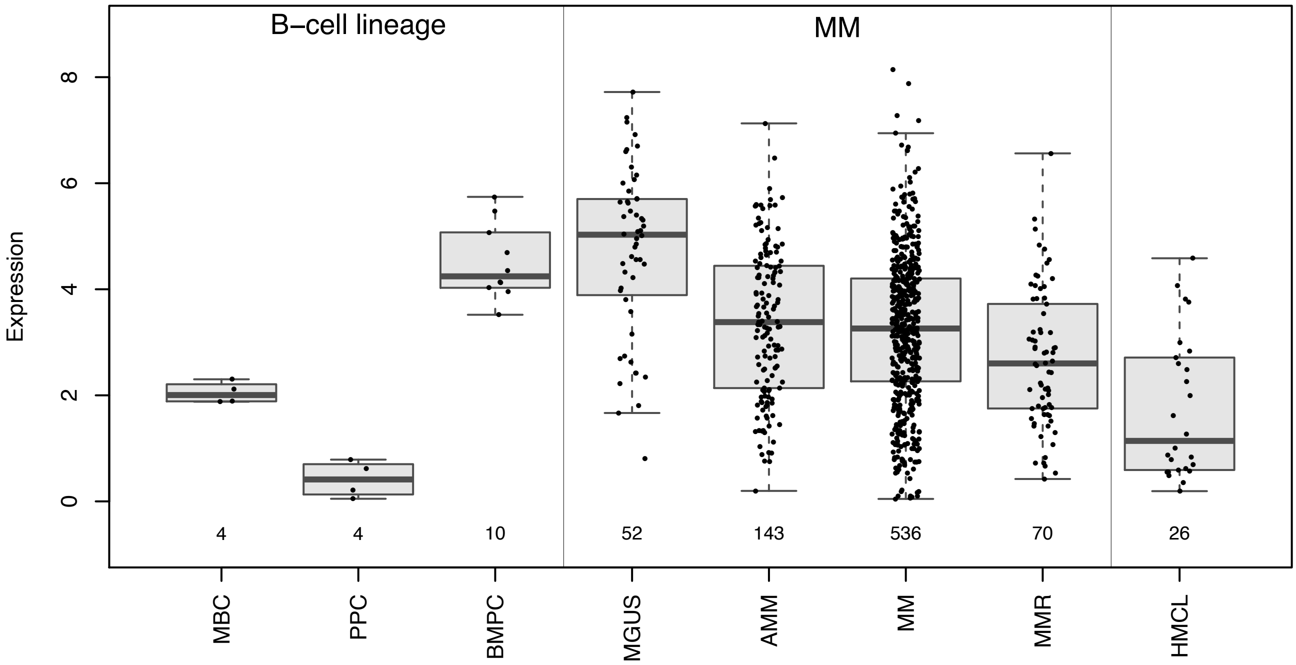

log2 RNA–Seq DCN (ENSG00000011465)

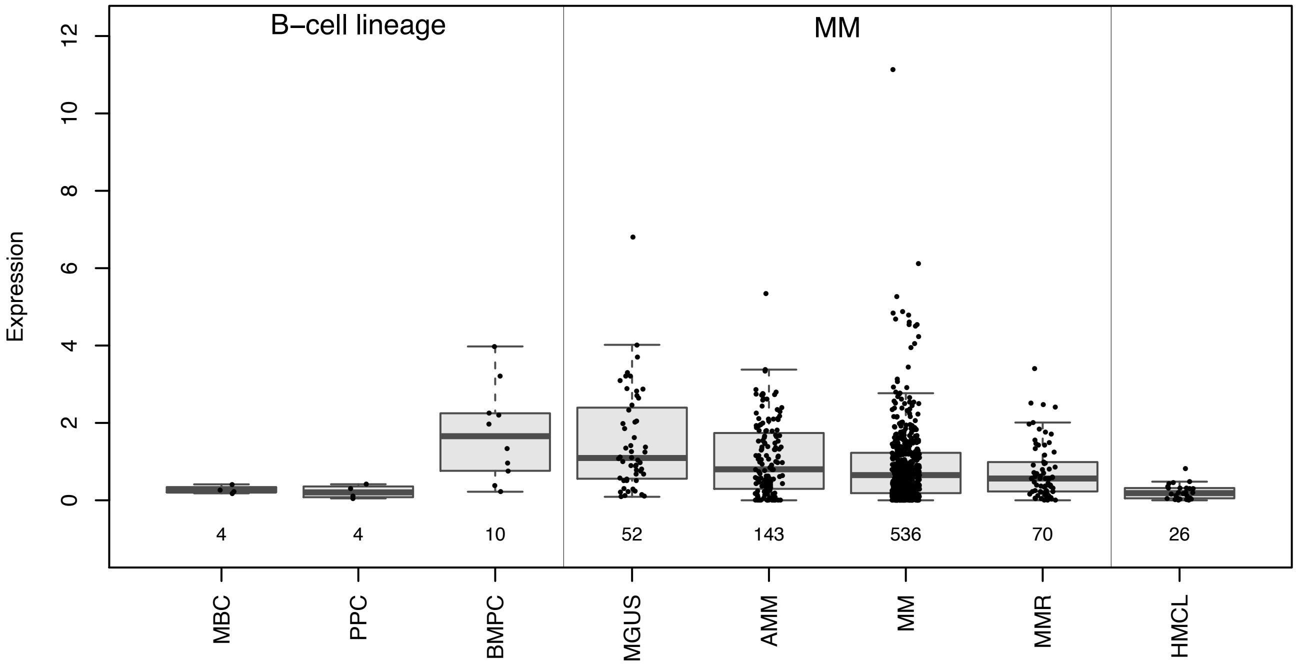

Supplementary Figure 7 (continued)

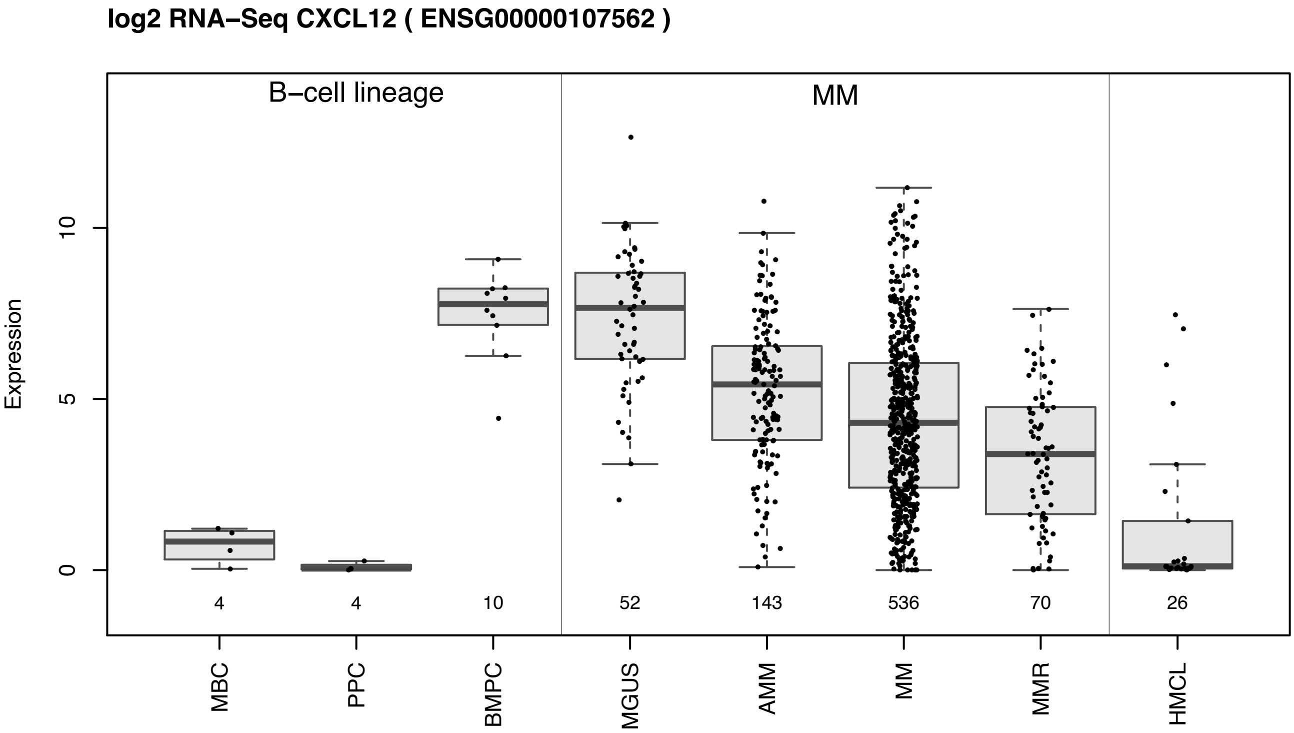

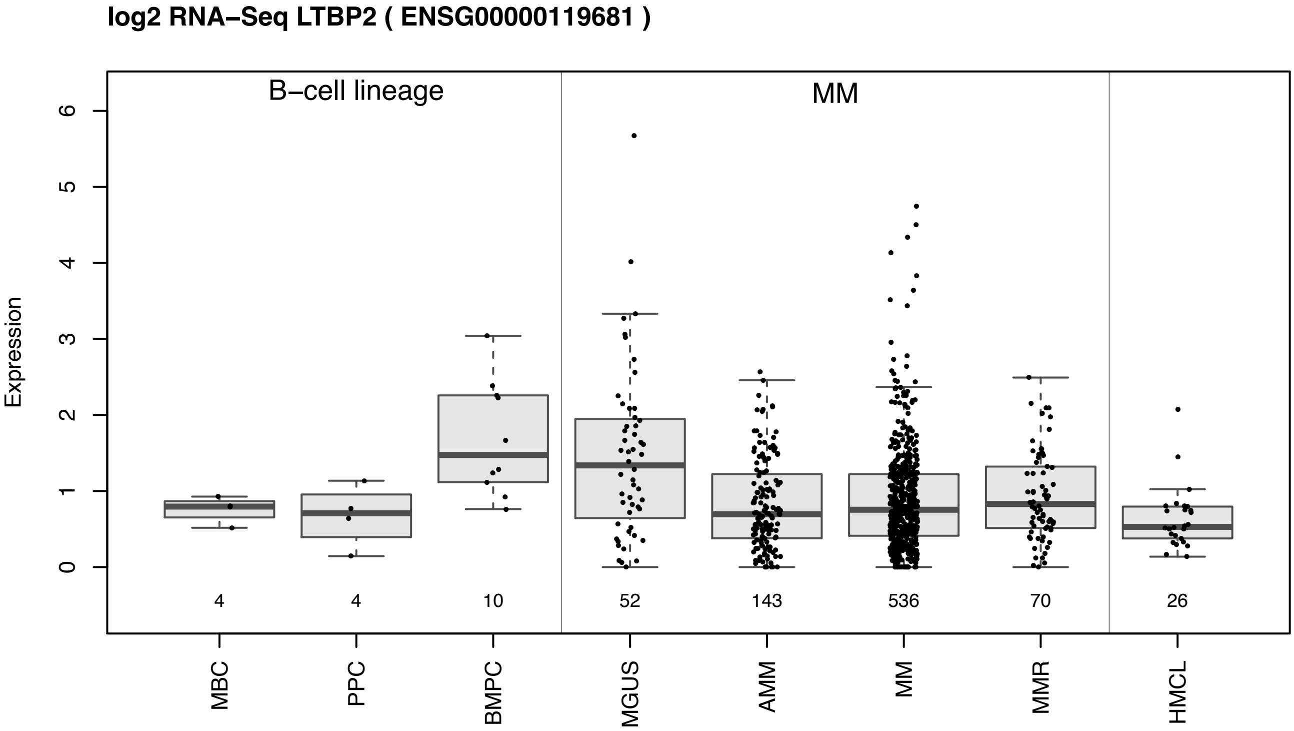

Supplementary Figure 7 (continued)

log2 RNA−Seq MYL9 (ENSG00000101335)

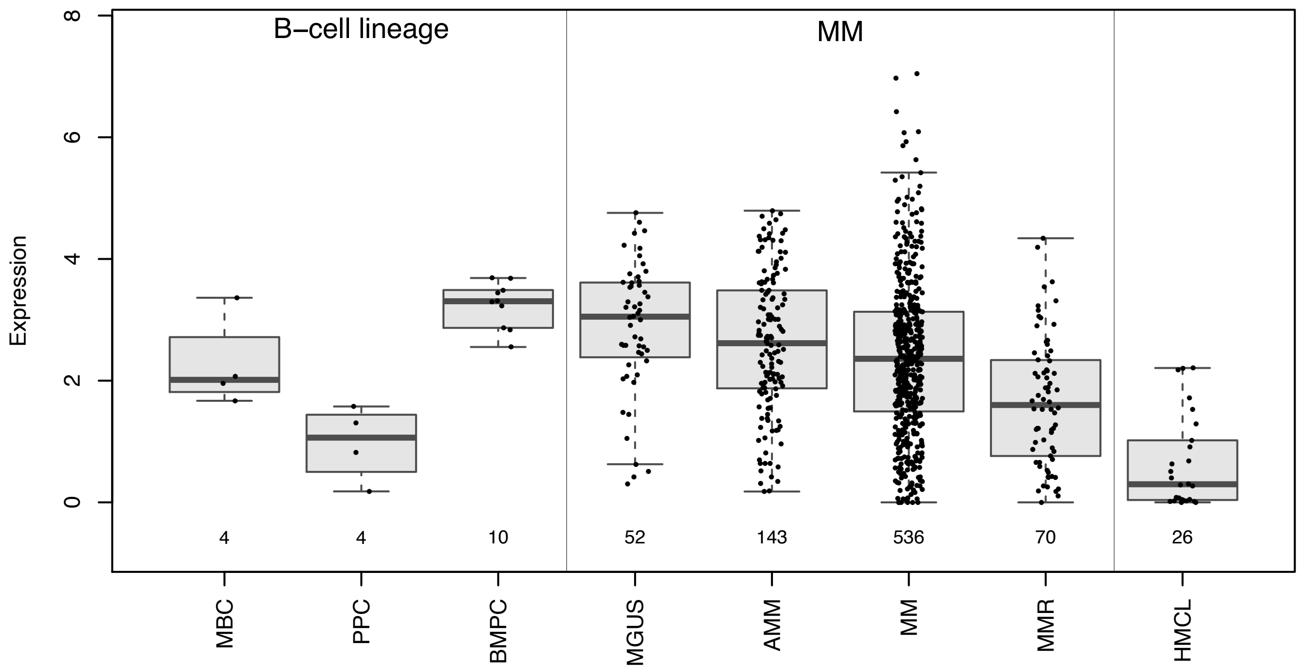

log2 RNA−Seq MFAP5 (ENSG00000197614)

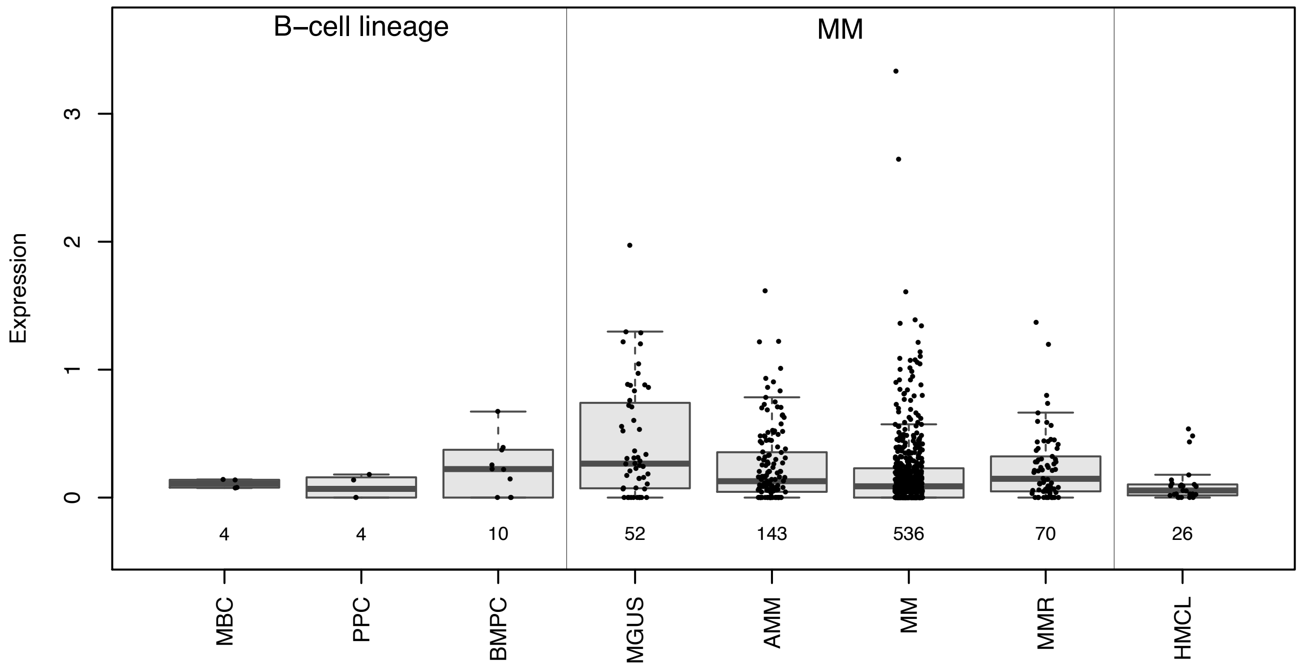

Supplementary Figure 7 (continued)

log2 RNA−Seq AXL (ENSG00000167601)

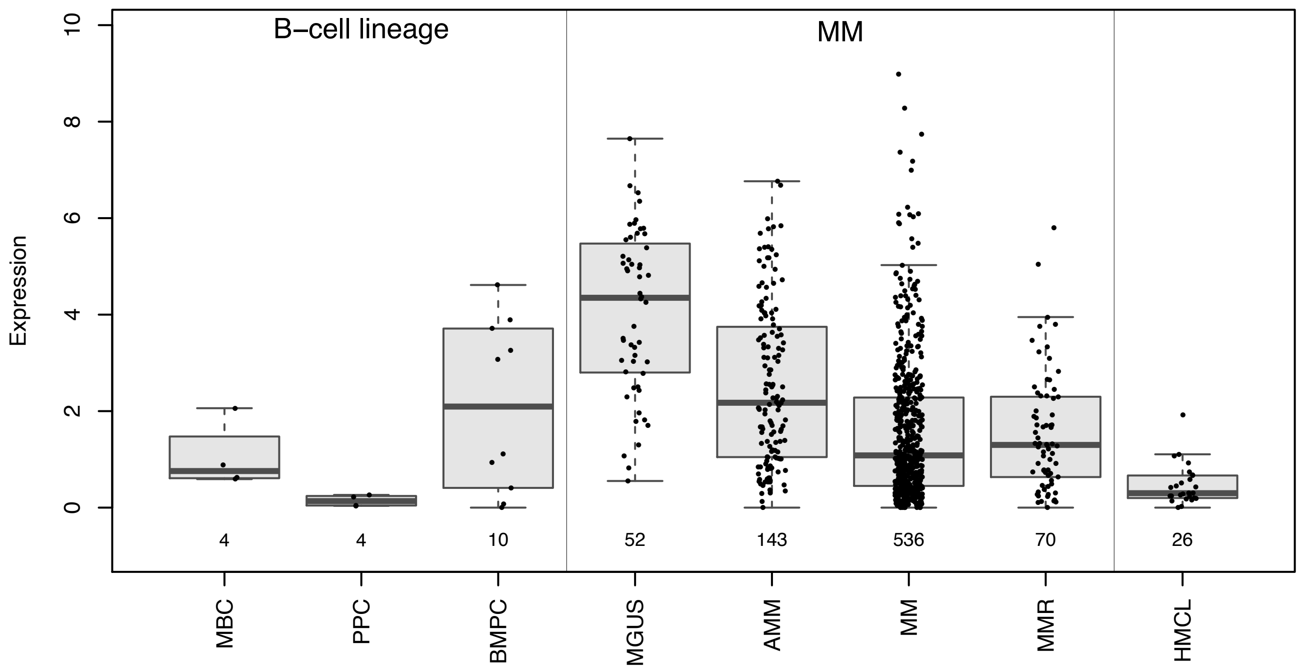

log2 RNA−Seq MMP14 (ENSG00000157227)

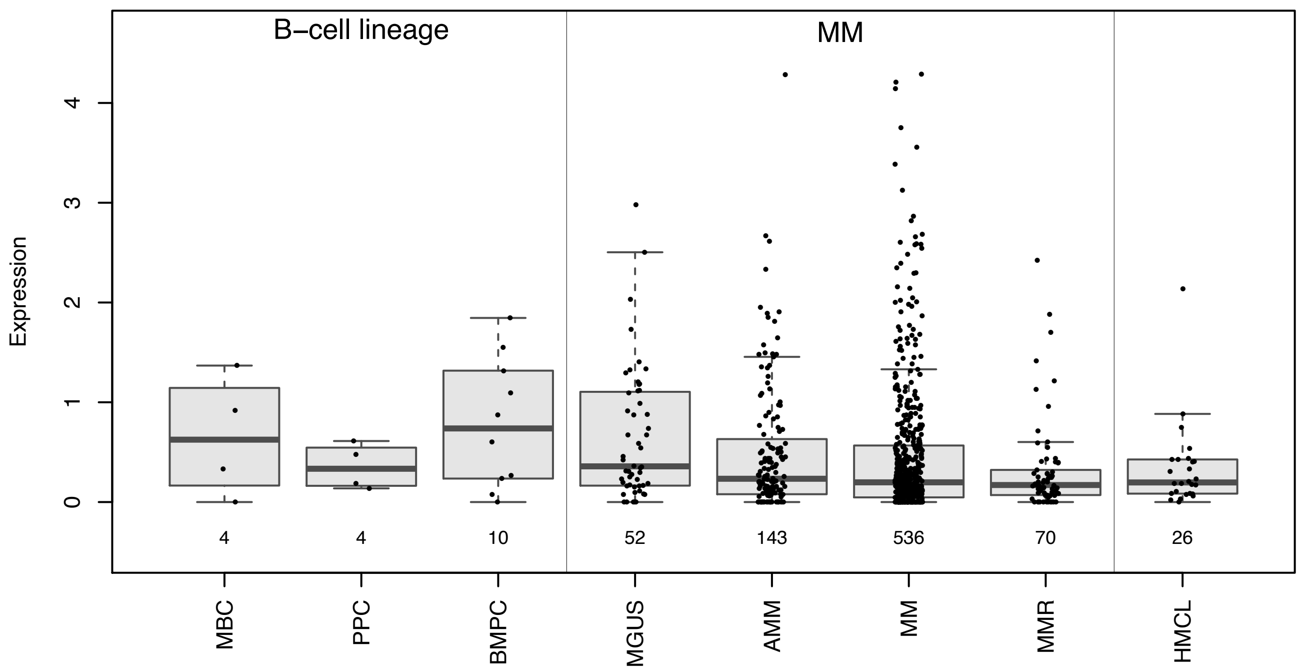

Supplementary Figure 7 (continued)

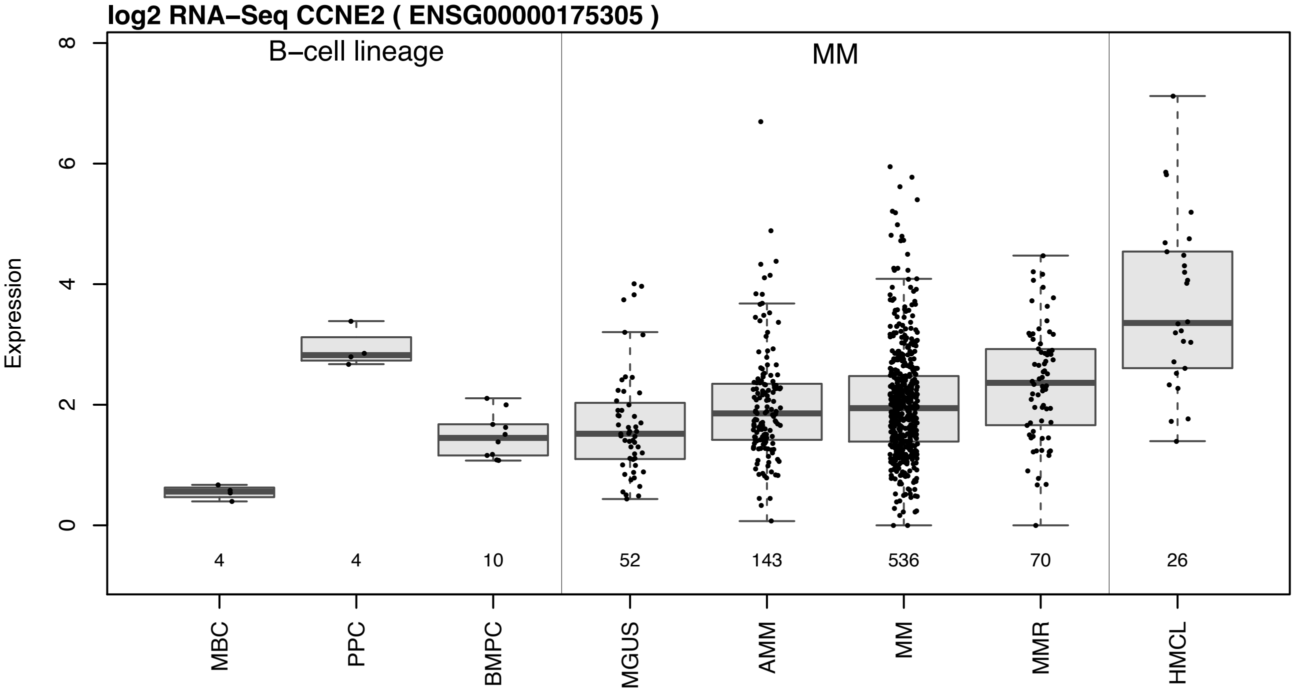
log2 RNA−Seq CCNE2 (ENSG00000175305)
B−cell lineage
MM
Expression
0
2
4
6
8
4
4
10
52
143
536
70
26
MBC
PPC
BMPC
MGUS
AMM
MM
MMR
HMCL

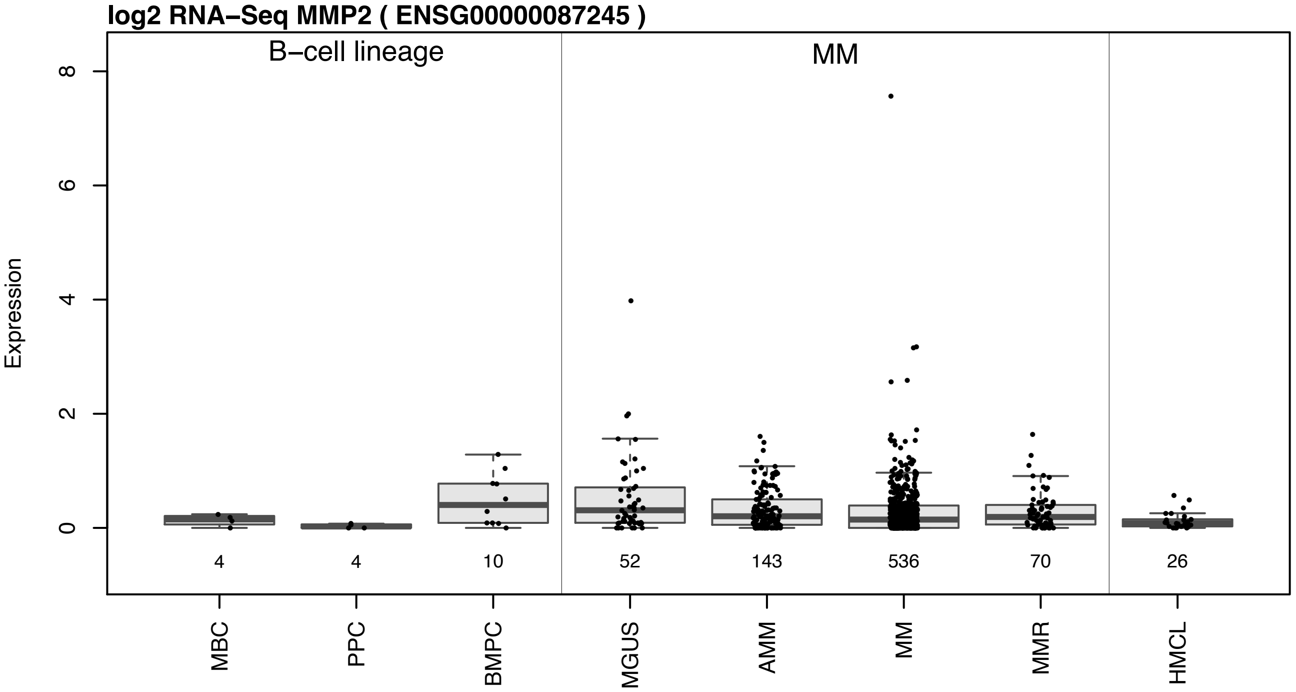
log2 RNA−Seq MMP2 (ENSG00000087245)
B−cell lineage
MM
Expression
0
2
4
6
8
4
4
10
52
143
536
70
26
MBC
PPC
BMPC
MGUS
AMM
MM
MMR
HMCL

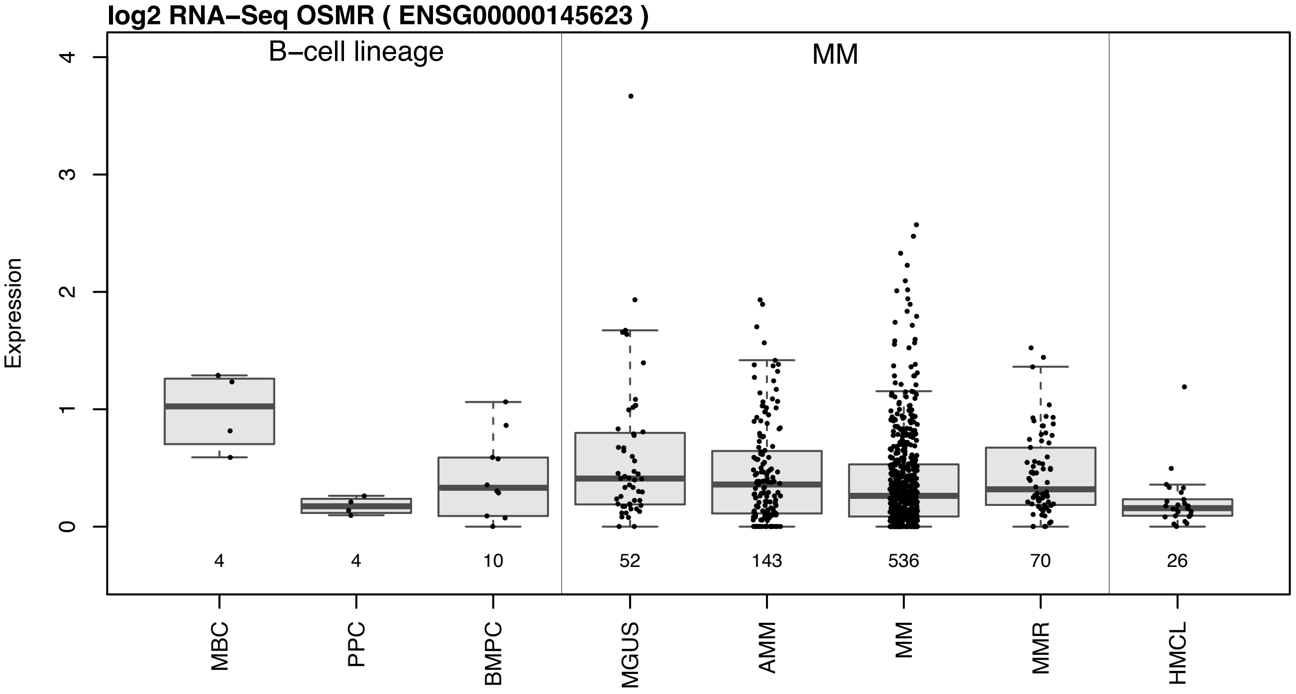
log2 RNA−Seq OSMR (ENSG00000145623)
B−cell lineage
MM
Expression
0
1
2
3
4
4
4
10
52
143
536
70
26
MBC
PPC
BMPC
MGUS
AMM
MM
MMR
HMCL

Supplementary Figure 8: Expression levels of adhesion genes that are not downregulated and associated with survival ($p < 0.01$). Bone Marrow Plasma Cell (BMPC), Monoclonal Gammopathy of Undetermined Significance (MGUS), Smoldering Multiple Myeloma (sMM), Multiple Myeloma (MM), Multiple Myeloma Relapse (MMR).
